# Biocatalytic Cascades toward Iminosugar Scaffolds Reveal Promiscuous Activity of Shikimate Dehydrogenases

**DOI:** 10.1021/acscentsci.2c01169

**Published:** 2023-01-11

**Authors:** Christopher R. B. Swanson, Grayson J. Ford, Ashley P. Mattey, Léa Gourbeyre, Sabine L. Flitsch

**Affiliations:** Manchester Institute of Biotechnology, School of Chemistry, The University of Manchester, 131 Princess Street, M1 7DNManchester, United Kingdom

## Abstract

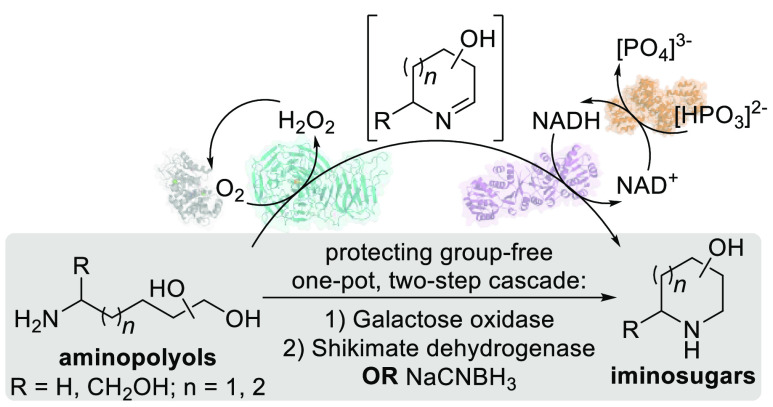

Iminosugar scaffolds are highly sought-after pharmaceutical targets, but their chemical synthesis is lengthy and can suffer from poor scalability and purification. Here we report protecting-group-free chemoenzymatic and biocatalytic cascades to synthesize iminosugars from sugar-derived aminopolyols in two steps. Using galactose oxidase variant F_2_ followed by a chemical or enzymatic reduction provided an efficient one-pot route to these targets, with product formation >70%. Key to success of this strategy was the application of genome mining, which identified bacterial shikimate dehydrogenases as promiscuous iminosugar reductases. The cell-free protocols allowed for isolation of highly polar iminosugar products from biotransformations in a single step through development of a gradient-elution cation exchange purification. The two-step pathway provides a short synthetic route that can be used as a cell-free platform for broader iminosugar synthesis.

## Abstract 2

We present a highly economic two-step one-pot enzyme cascade for synthesis of iminosugars, a class of bioactive clinically important compounds, exploiting promiscuous activity of metabolic enzymes.

## Introduction

Polyhydroxylated *N*-heterocycles, termed iminosugars, are an interesting target for chemical and biocatalytic synthesis. Such compounds occupy a unique and difficult to access chemical space due to their polarity, stereochemical density, and carbohydrate mimicry. Iminosugars possess a plethora of potent bioactivity as exemplified by their use as glycosidase inhibitors, antivirals, or pharmaceutical chaperones.^1−5^ One main challenge in the production of iminosugar therapeutics is their hydrophilicity, which complicates their discovery and isolation from natural sources.^1−4,6^

Although the chemical synthesis of iminosugars is well established,^2,7,8^ industrial scale-up can be problematic due to lengthy synthetic procedures, multiple protection–deprotection steps, expensive or toxic reagents, and poor scalability.^2,8,9^ Combined, these factors give rise to processes that are abjectly atom economic and difficult to implement. Furthermore, the extreme polarity of iminosugars causes many difficulties with analysis and isolation, often necessitating protection strategies to facilitate product purification.^10−12^ Ideally, routes toward iminosugars should be versatile, allowing a range of derivatives to be synthesized and isolated with ease and without any protecting groups.^13−16^

Biocatalytic routes toward these compounds are an attractive alternative as enzymes can be used in water at ambient temperatures and offer great control over chemo- and enantioselectivity. The chemoenzymatic cascade synthesis of many iminosugars has been demonstrated using D-fructose-6-phosphate aldolase (FSA) and palladium mediated hydrogenation.^13−16^ Other aldolases have also been applied to the synthesis of iminosugars, with the biocatalytic preparation of D-fagomine from glycerol and *N*-Cbz-3-aminopropanal via a multistep cascade in a flow reactor.^17^ More recently, the chemoenzymatic synthesis of several iminosugars was achieved in an *in vitro–in vivo* sequence using transaminases and *Gluconobacter oxydans* whole cells, followed by Pd mediated Cbz-deprotection and concomitant imine reduction.^18^ These routes provide a reduced number of synthetic steps and minimize the need for intermediate isolation. However, the use of *N*-protecting groups still limits the green credentials of these routes.

**Figure 1:**
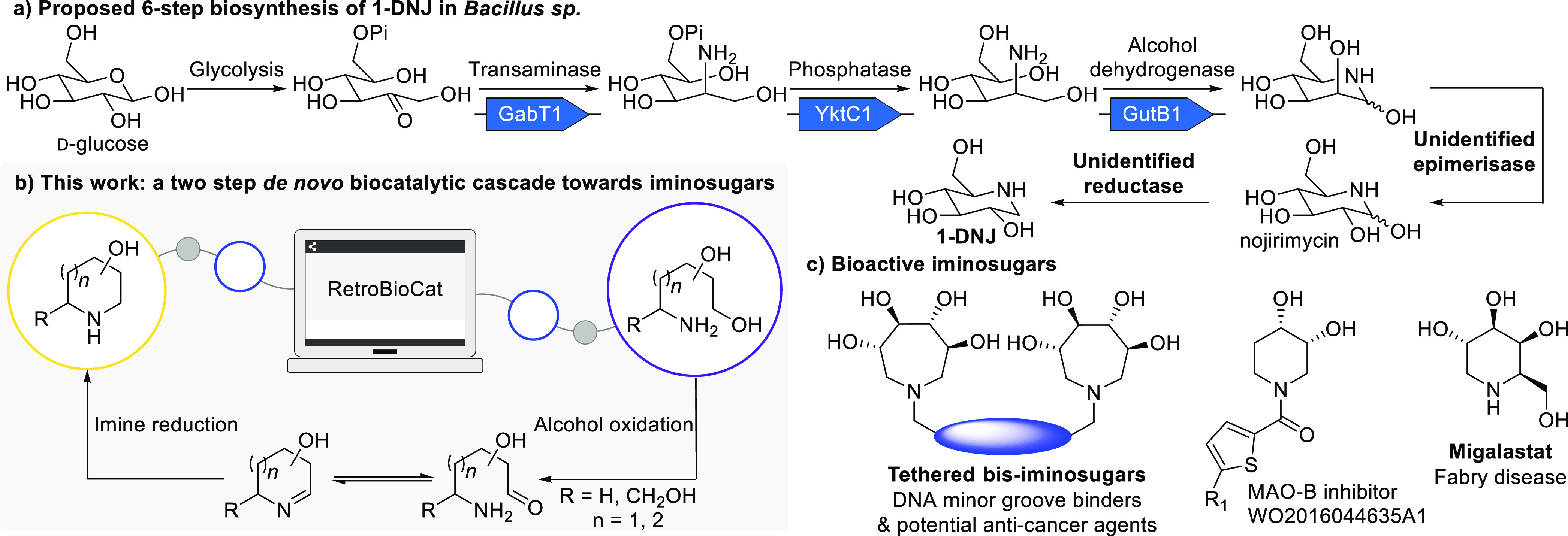
Overview of the biosynthetic origin of iminosugars in bacteria (a), the *de novo* biocatalytic cascade developed in this work (b), and example bioactive iminosugars (c).

Natural biosynthetic gene clusters that initiate iminosugar biosynthesis have been characterized, providing insight into the biosynthesis of 1-deoxynojirimycin (1-DNJ) and 1,4-dideoxy-1,4-imino-D-arabinitol (DAB-1) in bacteria.^19,20^ In both examples, the route incorporates a transamination, dephosphorylation, and alcohol dehydrogenation of glycolysis products such as D-fructose-6-phosphate, followed by a hypothesized epimerization and imine reduction (Figure 1a). Surprisingly, no enzyme has so far been identified for the proposed final steps of the biosynthesis in both cases. Even more surprisingly, heterologous expression of the 1-DNJ gene cluster in *Escherichia coli*, which does not naturally produce iminosugars, accomplishes 1-DNJ formation. This suggests that endogenous enzymes with broad promiscuous activity could be responsible for the final epimerization and reduction steps.^21,22^ Elucidation of these gene clusters paves the way for development of metabolic engineering approaches to iminosugar synthesis; however, until the final enzymes of the biosynthetic sequences toward these important bioactive compounds are identified, the impact of microbial fermentation approaches for synthesis of a range of iminosugar analogs is limited.^21,22^

There are also limitations with production of iminosugars via whole cell fermentation. These include competing metabolism pathways for sugar substrates, lengthy fermentation times (days), and complex downstream processing.^23^ Furthermore, the intracellular biosynthesis includes phosphorylation steps that are chemically nonessential for synthesis of the iminosugar scaffold. Their inclusion is likely to influence substrate recognition or transport through the cell. Such steps could be avoided to improve efficiency and brevity in a *de novo* biocatalytic cell-free cascade to iminosugars.

Applying biocatalytic retrosynthesis using RetroBioCat,^24,25^ we proposed a two-step *de novo* enzymatic cascade for the conversion of readily available sugar aminopolyols^26−28^ into iminosugars via primary alcohol oxidation and imine reduction (Figure 1b). With no protecting groups or phosphorylation steps this proposed route would be more concise than the biosynthetic sequence (Figure 1a,b). The required enzyme families had already been used in our group to access 3-aminopiperidines and azepanes.^29^ However, we were aware that identifying a suitable reductase for the final step, reducing the hydroxylated cyclic imine to the iminosugar product, would be a major challenge.

## Results and Discussion

### Screening for Oxidase Activity toward Free Aminopolyols

Galactose oxidase (GOase) is a copper radical alcohol oxidase which naturally oxidizes the C-6 alcohol of galactose and has been the subject of various evolution campaigns.^30,31^ As a result, a range of versatile GOase biocatalysts with broad and complementary substrate scope have been developed and subsequently applied in enzymatic cascades,^29,32−35^ making them an ideal candidate oxidase for the first step proposed here. Accordingly, purified GOase variants M_1_, M_3–5_, and F_2_ were screened against a panel of chemically synthesized sugar-derived aminopolyols using an HRP-ABTS colorimetric assay (Table 1).^30−32,36^ Pleasingly, activity was observed across most of the unprotected aminopolyol substrates tested, showing that GOase is tolerant of free amine containing substrates.^31−33^

**Table 1:**
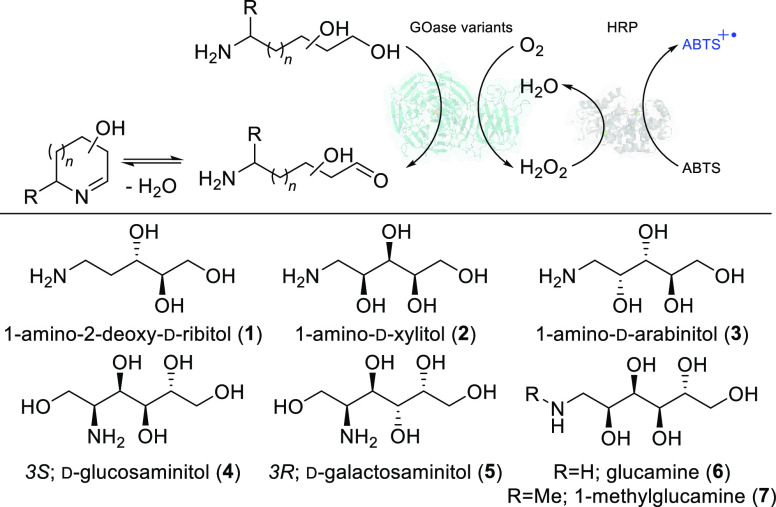
Specific Activity of GOase Variants toward Aminopolyol Substrates as Determined by an HRP-ABTS Colorimetric Assay^a^

	specific activity/μmol min^–1^ mg^–1^						
substrate	1	2	3	4	5	6	7
GOase M_1_				0.017	0.22		
GOase F_2_	0.14		0.066	0.41	0.16	0.25	0.17

a Assay conditions: purified GOase variants (0.03 mg mL^–1^), 25 mM substrate, 0.1 mg mL^–1^ HRP, 0.18 mg mL^–1^ ABTS in 100 mM NaPi buffer pH 7.4, 30 °C. Blank cells indicate no activity. No activity observed for GOase M_3–5_.

To verify this activity, GOase mediated biotransformations were performed and followed with sodium cyanoborohydride (NaCNBH_3_) to reduce any resulting imine species. Reaction mixtures were derivatized with a 6-aminoquinolylcarbonyl (AQC) tag followed by chromatographic analysis with UPLC-QDa.^37^ Iminosugar product (**1a**–**6a**) formation was monitored by comparison of newly appearing peaks with product *m*/*z* with the starting material peak. Products were compared with synthesized or commercial chemical standards where available. Formation of product **7a** was monitored by ^1^H NMR.

**Figure 2:**
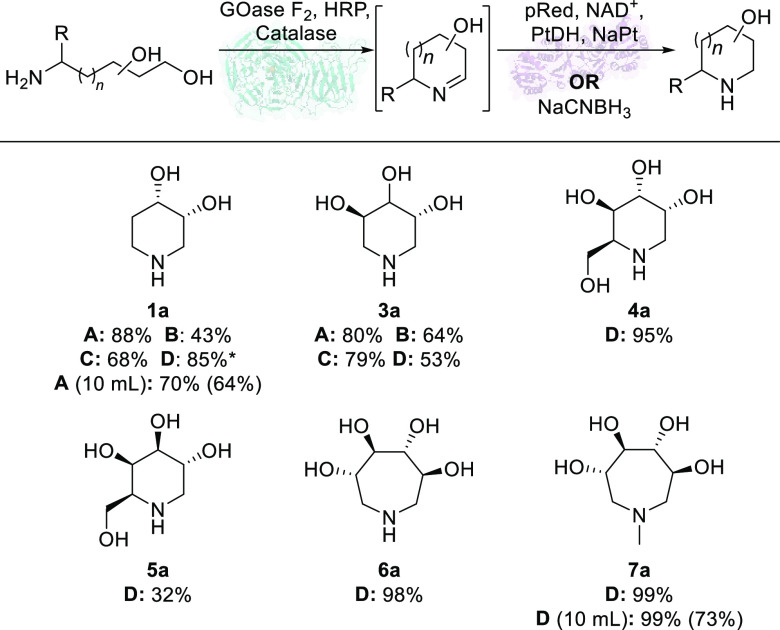
Substrate scope of the chemoenzymatic and biocatalytic conversion of amino alcohols **1**–**7** into iminosugars **1a**–**7a**. Conditions: substrate (10 mM), purified GOase F_2_ (1 mg mL^–1^), HRP (0.1 mg mL^–1^), catalase (0.1 mg mL^–1^), incubated at 25 °C, 5 h. Then, biotransformations were supplemented with either (A) purified pRed-14 (0.5 mg mL^–1^); (B) purified YdiB (0.5 mg mL^–1^); (C) purified AroE (0.5 mg mL^–1^) and PtDH cell-free extract (0.25 mg mL^–1^), NAD^+^ (0.5 mM), and NaPt (50 mM) for cofactor recycling; or (D) NaCNBH_3_ (50 mM). The total reaction volume was 1 mL. No product formation of **4a**–**7a** seen under conditions A, B, or C. Product formation determined by UPLC-QDa or ^1^H NMR. Reactions at 10 mL scale were performed as above but with immobilized GOase F_2_ (100 mg, 10 w% enzyme loading). Isolated yield of 10 mL reactions shown in brackets. * Byproduct formed only with chemical reducing agent in reaction **1aD**.

In this manner, GOase F_2_ activity toward 1-amino-2-deoxy-D-ribitol **1** and subsequent imine formation and reduction were confirmed, forming *cis*-3,4-dihydroxypiperidine **1a** (Figure 2). These reactions also showed that incubation of the GOase reaction mixture for long periods of time (>16 h) before addition of the chemical reducing agent led to side product formation, potentially due to oligomerization or tautomerization of the amino aldehyde or imine intermediates (Supporting Information, Figure S27). With the feasibility of the cascade established via this chemoenzymatic route, attention turned to the more challenging task of discovering an enzyme capable of performing the required imine reduction.

### Identification of Putative Iminosugar Reductases via Genome Mining

An in-house panel of 384 metagenomic imine reductases (IRED) had previously been screened in the oxidative direction against amine targets using a high-throughput colorimetric assay.^38,39^ However, establishing the activity of these enzymes against our targets is challenging due to the lack of available or isolable iminosugar imine intermediates for forward direction validation. A colorimetric screen performed with an iminosugar target returned only false positive hits, possibly due to oxidation of alcohols present in these substrates by endogenous enzymes present in lyophilized cell-free extract (Supporting Information, Figure S3).^39^

As no enzyme has yet been identified to act upon iminosugar imines, either biosynthetically or *in vitro*, we decided to conduct a MultiGeneBlast search on the biosynthetic gene clusters that produce DNJ and DAB-1 to identify any NAD(P)H dependent putative reductases that could carry out imine reduction to form iminosugars.^40^ A sample set of 24 putative enzymes (pRed-1–24) with broad classification and sequence homology were chosen. Multiple sequence alignments showed many of the “reductases” identified did not share conserved residues with the in-house 384 IRED panel, demonstrating the broad scope of the enzymes identified. Generating a diverse panel was our intention as many enzymes with imine reductase activity are often mislabeled or part of other superfamilies.^38^

### Development of Two-Step Enzymatic and Chemoenzymatic Cascades

As our target iminosugars are not commercially available, the identified reductases (pReds) were screened for initial activity in the one-pot cascade shown in Figure 2. Using 1-amino-2-deoxy-D-ribitol **1** as model substrate, biotransformations were set up using purified GOase F_2_ and reductase lysate. UPLC-QDa analysis of these biotransformations revealed two potential hits, pRed-14 and pRed-15, albeit with very low amounts of desired product **1a** formed (<5%). Several unidentified byproduct peaks were also present, likely due to interference of endogenous enzymes in the cell lysate used or cross reactions of GOase with the glucose employed in cofactor recycling. As such, pRed-14 and pRed-15 were purified for further testing.

Biotransformations using purified reductases were carried out in two ways: one as a one-pot telescopic reaction and the other as a sequential addition reaction where the reductase components were added 5 h after the GOase mediated reaction was initiated. We also envisioned that the recycling system commonly employed for nicotinamide cofactor regeneration, glucose/GDH, could interfere with the GOase, and so initial reactions were performed with stoichiometric NAD(P)H. Interestingly, the sequential addition reactions showed higher formation of **1a** from **1** than the telescopic reactions (Supporting Information, Figure S10). pRed-14 was more active in the cascade than pRed-15 and more efficient when NADH was used as a cofactor rather than NADPH. Notably, biotransformations of the model substrate **1** with purified pRed-14 eliminated the byproduct peaks seen in the chemical route using NaCNBH_3_, highlighting that the enzymatic reduction is more selective (Supporting Information, Figures S24–S27).

For scale-up, reaction optimizations with GOase F_2_ and pRed-14 shikimate dehydrogenase were carried out using **1** as a model substrate. It was observed that the cascade was more efficient at pH 8.0 due to enhanced stability of the imine intermediate, and that decreasing the reaction temperature to 25 °C yielded a small improvement in formation of **1a** (Supporting Information, Figures S6 and S7). The effect of substrate loading was also investigated with both free and immobilized GOase F_2_ (Supporting Information, Figures S8 and S9). Using purified GOase, only a slight reduction in product formation was seen from 5 mM substrate to 25 mM, although a significant decrease in formation of **1a** was seen with 50 mM substrate. A similar trend was observed with GOase F_2_ immobilized on Purolite ECR8285, a butyl methacrylate resin that had been applied previously with GOase.^41^ Decreasing the loading of pRed-14 from 1 to 0.25 mg mL^–1^ did not reduce product formation appreciably, although a steep decrease in product formation was observed upon decreasing GOase concentration (Supporting Information, Figures S14 and S15). Pleasingly, reintroduction of the glucose/GDH cofactor recycling system in sequential addition biotransformations improved product formation compared to those with stoichiometric NADH. GOase F_2_ has a high specific activity for glucose in comparison to the substrates of this cascade, and so cofactor recycling with phosphite dehydrogenase cell-free extract (PtDH) was also tested.^42^ A small increase in formation of **1a** was seen in sequential addition one-pot reactions, but a large improvement was observed in the telescopic one-pot reactions as the GOase substrate competition was eliminated (Supporting Information, Figure S10).

Expanding the reaction system to the whole substrate panel revealed that pRed-14 and pRde-15 only provided access to di- and trihydroxypiperidines **1a** and **3a**. However, the corresponding iminosugar products (**4a**–**7a**) from aminopolyols **4**–**7** were observed in biotransformations using the GOase-NaCNBH_3_ chemoenzymatic cascade. For the tetrahydroxylated azepane iminosugars **6a** and **7a**, biotransformations reached near quantitative formation of products with little optimization. This was particularly promising given this structural class of iminosugars was shown to be useful motifs in DNA minor groove binders and may possess important anticancer activity.^12^

### Scale-Up and One-Step Purification of Iminosugar Products

We then endeavored to perform preparative scale biotransformations of the developed cascades to characterize the resulting iminosugar products. Unfortunately, chemoenzymatic and biocatalytic one-pot conversion of substrates **1**, **4**, and **6** in batch reactions with purified, soluble GOase F_2_ were unsuccessful. Such transformations provided low (<20%) or no product formation due to protein precipitation, a phenomenon not observed in the previous analytical scale reactions. Hoping to overcome these issues with protein stability and enable enzyme reuse, we elected to run reactions using immobilized GOase. Pleasingly, batch reaction of substrate **1** with immobilized GOase and purified pRed-14 consistently provided over 70% formation of **1a** at 10 mL scale. The isolation of highly polar iminosugars from biotransformation mixtures has typically been challenging, and so we investigated several purification procedures. Pleasingly, a gradient elution cation-exchange approach with Dowex 50WX8 NH_4_^+^ form was very successful and allowed one-step isolation of pure product **1a** from the biotransformation mixture in 64% yield with comparable spectral data to the commercial standard (Supporting Information, Figures S37–S39). Using the same purification methodology with Dowex 50WX8 H^+^ form, compound **7a** was isolated in 73% yield from the chemoenzymatic transformation of **7** (Figure 2).

### Identification of Promiscuous Iminosugar Reductase Activity in Other Shikimate Dehydrogenases

To identify whether iminosugar reductase activity was a one-off promiscuous feature of pRed-14, or a more general activity of shikimate dehydrogenase enzymes, seven homologues of pRed-14 were further screened in this cascade. When screened as cell-free extracts, activity was again seen toward iminosugars **1a** and **3a** including good product formation using the well-known AroE and YdiB shikimate dehydrogenases (Figure 2, conditions B and C).^43^ As with pRed-14, use of purified AroE reduced formation of byproducts. This result supports the hypothesis that endogenous enzymes might be responsible for the final steps in iminosugar biosynthesis and could explain the lack of potential genes for these reductases in reported biosynthetic gene clusters. It would also explain iminosugar (1-DNJ) formation in heterologous expression systems of only the GabT1, YktC1, and GutB1 genes in *E. coli*, without any exogenous iminosugar reductase.^21^ We would hypothesize that the endogenous *E. coli* shikimate dehydrogenases can catalyze this transformation, thus providing a basis for further protein and metabolic engineering studies, in particular toward unnatural iminosugars.

**Figure 3:**
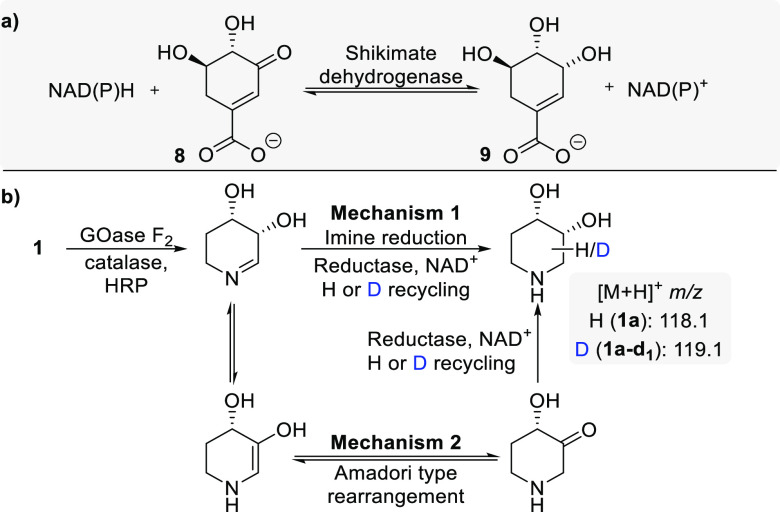
Natural reactivity of shikimate dehydrogenases (a) and labeling studies to confirm enzymatic hydride transfer by two plausible mechanisms (b).

To gain further insight into the developed iminosugar producing cascade, isotopic labels were introduced using a deuterium-based recycling system (Figure 3b), confirming that the reductase was indeed involved in hydride transfer to the substrate. Using GDH and D-glucose-1-*d*_1_ as the sacrificial substrate, deuterated NADD cofactor would be formed *in situ* and the deuteride transferred to the iminosugar during the SDH catalyzed step. Analysis of these reactions by UPLC-QDa confirmed that the SDH enzyme is involved in hydride transfer to the reaction intermediate as product peaks with *m*/*z* corresponding to **1a-*d***_**1**_ [M + H]^+^ were observed (Supporting Information, Figure S34).

It was envisaged that two mechanisms were plausible for this transformation and, indeed, in the biogenesis of iminosugars, as proposed by Horenstein et al.^19^ The enzymatic reduction may proceed either through IRED activity on the intermediate imine or via an Amadori rearrangement, forming an iminosugar aminoketone which is then reduced enzymatically (Figure 3b). The latter, being reduction of a ketone, would more closely relate to the natural reaction of shikimate dehydrogenases—the reversible ketone reduction of 3-dehydro-shikimate **8** to shikimate **9** (Figure 3a).

### Safety Statement

No unexpected or unusually high safety hazards were encountered.

## Conclusion

The *de novo* chemoenzymatic and biocatalytic cascades developed herein have allowed access to several interesting iminosugar scaffolds in two steps from easily obtainable or commercial starting materials. This route is considerably more economical in steps when compared to reported chemical methods and does not require N- or O-protection. Key to success was identification of promiscuous reductase activity of shikimate dehydrogenases for iminosugar synthesis which provides a basis for further evolution of a broad panel of previously unprecedented iminosugar reductases. Furthermore, the promiscuous activity of metabolic shikimate dehydrogenases discovered in this work provides support for the hypothesis that enzymes from primary metabolism act as endogenous catalysts for the final steps of biosynthesis in iminosugar producing organisms and may finally answer these fundamental questions about the biosynthesis of iminosugars.

## Supporting Information Available

Materials and methods, detailed experimental procedures, protein sequences, expression and purification procedures, full UPLC analysis and optimization data, UPLC-QDa methods and traces, synthetic procedures, and NMR characterization data (PDF)
Transparent Peer Review report available (PDF)
