# Supplementary material for: Biocatalytic Cascades toward Iminosugar Scaffolds Reveal Promiscuous Activity of Shikimate Dehydrogenases
Source: ACS Cent Sci. 2023 Jan 11;9(1):103–8. doi: 10.1021/acscentsci.2c01169 (PMC9881201; doi:10.1021/acscentsci.2c01169)
Supplement: Supplementary file 1 — oc2c01169_si_001.pdf [file oc2c01169_si_001.pdf]

Supporting information for

Biocatalytic cascades towards iminosugar scaffolds reveal promiscuous activity of shikimate dehydrogenases

Christopher R. B. Swanson, Grayson J. Ford, Ashley P. Mattey, Léa Gourbeyre, Sabine L. Flitsch*

Manchester Institute of Biotechnology, School of Chemistry, The University of Manchester, 131 Princess Street, M1 7DN, Manchester, United Kingdom

*Email: Sabine.flitsch@manchester.ac.uk

Contents

Materials and methods 2
Analytical methods....3
AQC derivatisation....3
Enzyme immobilisation 3
Biocatalyst Production 3
Galactose oxidase (GOase) production 4
Reductase production 4
Reductase purification 5
SDS-PAGE analysis....5
Protein sequences....5
Reductase identification 8
Identification of new putative reductases....8
Colourimetric reductase screening....8
Initial activity screening 9
Galactose oxidase liquid phase assay....9
Assay to determine inhibition of GOase by iminosugar products 10
Biotransformation Methods 11
Chemoenzymatic GOase-$NaCNBH_3$ cascade biotransformations....11
Screening of proposed GOase-Reductase cascade 11
Optimisation of GOase-pRed cascade with 1-amino-2-deoxy-D-ribitol....12
Substrate scope of the biocatalytic and chemoenzymatic cascades....18
General procedure for biotransformations & reaction purification 18
Mechanistic insight into SDH catalysed reduction....20

Possible tautomerisation and amadori rearrangement pathways 20
Deuteration experiments used to investigate reduction mechanism 20
UPLC-QDa chromatograms 21
Substrates and standards 21
Biotransformations 23
Determination of product formation by NMR 28
Characterisation of biocatalytically synthesised products 29
3,4-dihydroxypiperidine **1a** 29
N-methyl-3,4,5,6-tetrahydroxyazepane **7a** 31
Chemical synthesis 33
1-amino-2-deoxy-D-ribose.HCl (**1**) 33
1-amino-D-xylitol.HCl (**2**) 33
1-amino-D-arabinitol.HCl (**3**) 33
Glucosaminitol.HCl (**4**) 34
Galactosaminitol.HCl (**5**) 34
NMR spectra for chemically synthesised compounds 35
References 40

Materials and methods

All commercial reagents and solvents used in this work were purchased from Sigma-Aldrich (Poole, Dorset,UK), Alfa-Aesar (Heysham, Lancashire, UK), Acros Organics (Loughborough, UK), Santa Cruz Biotechnology (Dallas, Texas, U.S.A.) or Fluorochem (Hadfield, Derbyshire, UK) and used without further purification. Isopropyl β-D1-thiogalactopyranoside (IPTG), kanamycin, Terrific broth (TB) and LB (Luria-Bertani) Agar Miller was purchased from Formedium (Hunstanton, England). Co-factors were obtained from Prozomix Ltd (Haltwhistle, Northumberland, UK). Unless stated otherwise, a Bruker Avance 400 MHz spectrometer was used to record NMR spectra with chemical shifts reported in ppm relative to tetramethylsilane (TMS). Coupling constants (J) are reported in Hz. Reverse phase UPLC-QDa analysis was carried out on a Waters Acquity H-class system with sample manager (model J15SD1368G), column heater (model H15CHA), FLR detector (Model G15UPF045G), TUV detector (model E16TUV487A), QDa detector (model KAB1525). A HSS C18 1.8 µm, 2.1 x 100 mm column was used as stationary phase. Normal phase HILIC UPLC-QDa was carried out on the same instrument using a Glycan BEH amide 130 Å, 1.7 µm, 2.1 x 150 mm column as stationary phase. Thompson UPLC grade nano filter vials with PVDF membrane, 0.45 µm pore size were used for all samples and standards. Reverse phase UPLC-QDa was applied for all biotransformations of 1-amino-2-deoxy-D-ribitol **1** and related standards. Normal phase HILIC UPLC-QDa was used for standards and biotransformations of all other substrates.

Analytical methods

Table S1: HILIC-UPLC-QDa method used in this work. Solvent A = 50 mM NH_4HCO_2 pH 4.4, solvent B = Acetonitrile. This method was applied with a Glycan BEH amide 130 Å, 1.7 µm, 2.1 x 150 mm column as stationary phase.

Time / min	Flow rate / mL min^{-1}	% solvent A	% solvent B
0.00	0.25	10	90
7.00	0.25	30	70
9.00	0.25	90	10
12.00	0.25	90	10
12.10	0.25	10	90
15.00	0.25	10	90

Table S2: RP-UPLC-QDa method used in this work. Solvent A = dH_2O + 0.1% DFA, solvent B = Acetonitrile + 0.1% DFA. This method was applied with a HSS C18 1.8 µm, 2.1 x 100 mm column as stationary phase.

Time / min	Flow rate / mL min^{-1}	% solvent A	% solvent B
0.00	0.5	95	5
3.00	0.5	93.5	6.5
3.10	0.5	0	100
4.10	0.5	0	100
4.20	0.5	95	5
6.00	0.5	95	5

AQC derivatisation

Stocks of the AQC-tag reagent (6-Aminoquinolyl-N-hydroxysuccinimidyl carbamate) were made up at 10 mM in dry acetonitrile and stored at -80 ^{o}C as single use aliquots.

For analysis by reverse phase UPLC-QDa, derivatisation was accomplished by adding amine sample (5 mM, 10 µL) to borate buffer (100 mM, pH 8.8, 80 µL) followed by AQC-tag reagent (10 mM in ACN, 10 µL). The mixture was vortexed then incubated at 55 ^{o}C, 300 rpm for 15 minutes. Samples were centrifuged (13k rpm, 10 minutes) and decanted into UPLC grade filter vials.

For analysis by normal phase HILIC UPLC, the above procedure was followed except borate buffer was replaced by an equal volume of 1:1 acetonitrile/borate buffer (100 mM, pH 8.8).

Enzyme immobilisation

Immobilised GOase F_2 was prepared as described in the literature.[1] Purolite butylmethacrylate ECR8285 resin (10–20 mg) was washed with NaPi/NaCl buffer (25 mM NaPi, 100 mM NaCl, 3 x 1 mL). GOase F_2 (10 % w/w, 1–2 mg) was added and the solution made up to 1 mL with NaPi/NaCl buffer. The suspension was gently agitated at ambient temperature for 16 h then allowed to stand for a further 24 h. The beads were washed with reaction buffer (typically 100 mM NaPi pH 7.4) and stored at 4 ^{o}C until use. Immobilisation yields were monitored by Nanodrop absorbance or BSA assay, and were comparable to previous reports.

Biocatalyst Production

Chemically competent E. coli cells were transformed with DNA plasmid vectors that contain genes for the desired protein (biocatalyst) and grown on LB agar plates containing 30 μg mL^{-1} antibiotic (kanamycin) as shown in Table S1.

Table S3: Biocatalysts vectors and cell strains used in this work. BL–21 cells were purchased from New England Biolabs and BL–21* cells were purchased from Qiagen.

Protein	Organism	Plasmid	Cell strain
GOase M_1	*Fusarium graminearum*	pET30	BL–21*
GOase M_{3-5}	*Fusarium graminearum*	pET30	BL–21*
GOase F_2	*Fusarium graminearum*	pET30	BL–21*
pRed-14	*Paenibacillus sp. FSL H7-0737*	pET28a	BL–21
pRed-15	*Bacillus subtilis*	pET28a	BL–21
YdiB	*E. coli*	pET28a	BL–21
AroE	*E. coli*	pET28a	BL–21
PtDH	*Pseudomonas stutzeri*	pET28a	BL–21

Galactose oxidase (GOase) production

A single colony harbouring a GOase gene was selected and used to inoculate 5 mL of lysogeny broth (LB) medium containing kanamycin (final concentration 30–50 μg mL^{-1}) in a 15 mL falcon tube and incubated overnight at 37 °C shaking at 250 rpm. 500 μL of the preculture was used to inoculate a 250 mL of auto induction medium (8ZY-4LAC) supplemented with 250 μL of kanamycin (30 mg mL^{-1}) in a 2 L baffled flask and further incubated at 250 rpm at 26 °C for 60 h. The cell cultures were harvested by centrifugation at 4000 rpm for 30 min at 4 °C and the supernatant was discarded. The cell pellets were washed with NaPi buffer (100 mM, pH 7.4) and centrifuged again using the same conditions and the supernatant discarded. The wet cell pellets were stored at -20 °C. To purify the protein the thawed cell pellets of one 400 mL culture were first resuspended in NP buffer (50 mM Na_2PO_4, 100mM NaCl, pH 8.0, 25 mL) with Triton X 100 lysis buffer (250 uL) and lysozyme from egg white (1 mg mL^{-1}) and incubated at 4 °C for 20 min with gradual shaking. The suspension was lysed by ultra-sonification (30 sec ON, 90 sec OFF, 5x cycles) while submerged in an ice bath. The lysate was clarified by centrifugation at 20,000 rpm for 40 min at 4 °C. The supernatant was then loaded onto a Strep-TagII® column which had been equilibrated with 50 mL NP Buffer and the column was subsequently washed with 30 mL NP buffer to remove any non-tagged proteins. The GOase was eluted with 40 mL NPD buffer (50 mM Na2PO4, 300 mM NaCl, 5 mM desthiobiotin, pH 8) and concentrated by centrifugation in 30,000 MWCO PES vivaspin columns. The protein was dialysed overnight at 4 °C against 50 mM NaPi (pH 7.4) supplemented with copper sulphate. After, the protein was dialysed again at 4 °C against NaPi buffer to remove excess Cu. The protein concentration was then determined using a NanoDrop™ spectrometer (typically 3–5 mg ml^{-1}). The samples were divided into 1.5 mL Eppendorf tubes, flash-frozen and then stored at -80 °C until they were used in the biotransformation reactions. Typical expression yields for GOase variants were 200 – 250 mg/L culture broth, in line with previous reports.[2]

Reductase production

A single colony harbouring a reductase gene was added to 20 mL LB medium supplemented with 20 µL kanamycin (30 mg mL^{-1}) and incubated overnight at 250 rpm and 30 °C. The full volume of this preculture was used to inoculate a 400 mL of TB media, supplemented with 400 µL of kanamycin, in a 2 L baffled flask and further incubated at 37 °C, 250 rpm until optical cell density (OD600) of 0.6 was reached. At this point, protein expression was induced by inoculating the flask with 400 µL of isopropyl-beta-D-1-thiogalactopyranoside (IPTG, 0.1 M). The flask was then incubated at 22 °C at 200 rpm for 20 h. The cells were harvested by centrifugation at 4000 rpm for 30 min at 4 °C. The cell pellets were washed with NaPi buffer (100 mM, pH 7.4) and centrifuged again using the same conditions and the supernatant discarded. The cell pellets were stored at -20 °C in 50 mL falcon tubes until further use. For preparation of cell free extracts, the thawed cell pellets were resuspended in 4–5 mL (per g of cell pellet) in NaPi buffer (100 mM, pH 7.4) and lysed by ultra-sonication (60 sec ON, 120 sec OFF, 40 AMP, 4 cycles) while samples were submerged in an ice bath. The lysed cells were clarified by ultra-centrifugation at 18,000 rpm at 4 °C for 60 min. The clarified supernatant was filtered through a cellulose membrane (0.45 µm), frozen at -80 °C then lyophilised on a Buchi Lyovapor-200. Lyophilised cell free extract was stored at -20 °C until further use.

Reductase purification

Thawed cell pellets were resuspended in 4–5 mL (per g of cell pellet) in 10 % Buffer B (100 mM NaPi buffer, 300 mM NaCl, 30 mM imidazole, pH 7.5) and lysed by ultra-sonication (60 sec ON, 120 sec OFF, 40 AMP, 4 cycles) while samples were submerged in an ice bath. The lysed cells were clarified by ultra-centrifugation at 18,000 rpm at 4 °C for 60 min. The clarified supernatant was filtered through a cellulose membrane (0.45 µm) and loaded onto a His-Trap Crude FF column (GE Healthcare) charged with 0.1 M nickel sulphate equilibrated with 10% buffer B. The column was washed with 15 mL 10% buffer B and then 15 mL 20% buffer B. His-tagged protein was then eluted with 100% buffer B and 1–2 mL fractions were collected. The protein concentration of each fraction was monitored by a thermofisher NanoDrop™ microvolume spectrophotometer. Fractions containing pure protein were combined and then concentrated using a membrane 30,000 MWCO PES vivaspin columns to remove excess imidazole and exchange into NaPi buffer until the desired concentration was achieved (typically 10 mg mL^{-1}). The pure protein was divided into 1 mL aliquots, flash frozen and stored at -80 °C until further use. Expression yields of reductase proteins were between 100–150 mg/L of culture broth.

SDS-PAGE analysis

SDS PAGE was carried out on BioRad premade gel in 1X TGS running buffer. Samples were loaded with 50% Lameli buffer purchased from Sigma Aldrich, Molecular weight marker was purchased from New England Biolabs.

Ladder 1 2 3 4 5 6 7 Ladder 8

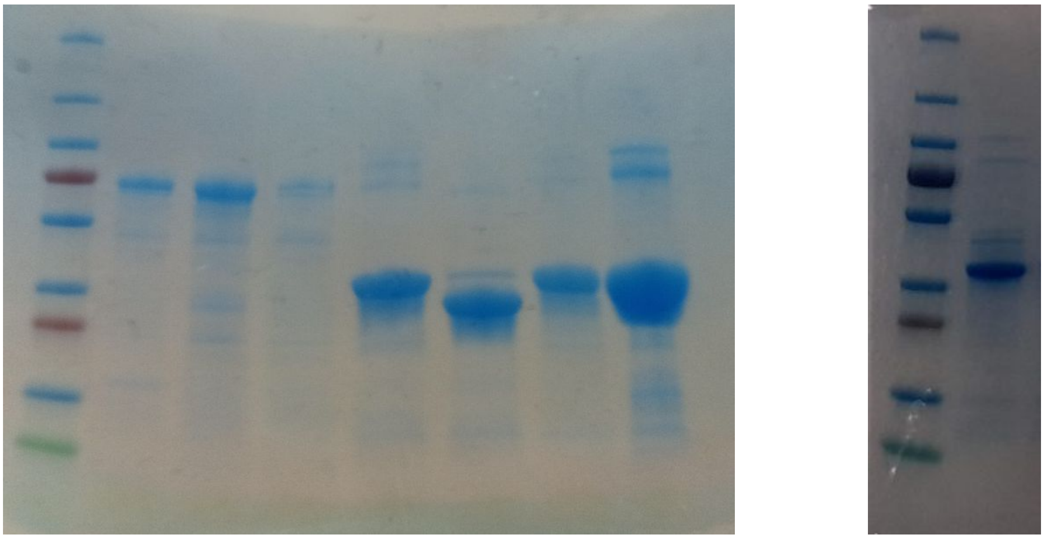

Figure S1: SDS-PAGE analysis of proteins used in this work. Left to right: GOase F_2, GOase M_{3-5}, GOase M_1, pRed-14, AroE, YdiB, pRed-15, PtDH lysate.

Protein sequences

GOase M_1

MASAPISGAISRNNWAVTCDPAQSGNECNKAIDGNKDTFWHTFYGANGDPKPPHTYTIDMKT
TQNVNGLSVLPRQDGNQNGWIGRHEVYLSSDGTNWGSPVASGSWFADSTTKYSNFETRPARY
VRLVAITEANGQPWTSIAEINVFQASSYTAPQPGLGRWGPTIDLPIVPAAAAIEPTSGRVLMWSS
YRNDAFEGSPGGITLTSSWDPSTGIVSDRTVTVTKHDMFCPGISMDGNGQIVVTGGNDAKKTSL
YDSSSDSWIPGPDMQVARGYQSSATMSDGRVFTIGGSWSGGVFEKNGEVYSPSSKTWTSLPNA
KVNPMLTADKQGLYRSDNHAWLFGWKKGSVFQAGPSTAMNWYYTSGSGDVKSAGKRQSNR
GVAPDAMCGNAVMYDAVKGKILTFGGSPDYQDSDATTNAHIITLGEPGTSPNTVFASNGLYFA
RTFHTSVVLPDGSTFITGGQRRGIPFEDSTPVFTPEIYVPEQDTFYKQNPNSIVRAYHSISLLLPDG
RVFNGGGGLCGDCTTNHFDAQIFTPNYLYDSNGNLATRPKITRTSTQSVKVGGRITISTDSSISKA
SLIRYGTATHTVNTDQRRIPLTLTNNGGNSYSFQVPSDSGVALPGYWMLFVMNSAGVPSVASTI
RVTQGGGGSWSHPQFEK

GOase F_2

MASAPISGAISRNNWAVTCDPAQSGNECNKAIDGNKDTFWHTFYGANGDPKPPHTYTIDMKT
TQNVNGLSVLPRQDGNQNGWIGRHEVYLSSDGTNWGSPVASGSWFADSTTKYSNFETRPARY
VRLVAITEANGQPWTSIAEINVFQASSYTAPQPGLGRWGPTIDLPIVPAAAAIEPTSGRVLMWSS
YRNDAFEGSPGGITLTSSWDPSTGIVSDRTVTVTKHDMFCPGISMDGNGQIVVTGGNDAKKTSL
YDSSSDSWIPGPDMQVARGYQSSATMSDGRVFTIGGSFSGGVFEKNGEVYSPSSKTWTSLPNAK
VNPMLTADKQGLYKSDNHAWLFGWKKGSVFQAGPSTAMNWYYTSGSGDVKSAGKRQSNRG
VAPDAMCGNAVMYDAVKGKILTFGGSPDFEDSDATTNAHIITLGEPGTSPNTVFASNGLYFART
FHTSVVLPDGSTFITGGQRRGIPFEDSTPVFTPEIYVPEQDTFYKQNPNSIVRAYHSISLLLPDGRV
FNGGGGLCGDCTTNHFDAQIFTPNYLYDSNGNLATRPKITRTSTQSVKVGGRITISTDSSISKASL
IRYGTATHTVNTDQRRIPLTLTNNGGNSYSFQVPSDSGVALPGYWMLFVMNSAGVPSVASTIRV
TQGGGGSWSHPQFEK

GOase M_{3-5}

MASAPISGAISRNNWAVTCDPAQSGNECNKAIDGNKDTFWHTFYGANGDPKPPHTYTIDMKT
TQNVNGLSVLPRQDGNQNGWIGRHEVYLSSDGTNWGSPVASGSWFADSTTKYSNFETRPARY
VRLVAITEANGQPWTSIAEINVFQASSYTAPQPGLGRWGPTIDLPIVPAAAAIEPTSGRVLMWSS
YRNDAFEGSPGGITLTSSWDPSTGIVSDRTVTVTKHDMFCPGISMDGNGQIVVTGGNDAKKTSL
YDSSSDSWIPGPDMQVARGYQSSATMSDGRVFTIGGSFSGGVFEKNGEVYSPSSKTWTSLPNAK

VNPMLTADKQGLYMSDNHAWLFGWKKGSVFQAGPSTAMNWYYTSGSGDVKSAGKRQSNRG
VAPDAMCGNAVMYDAVKGKILTFGGSPDYTDSDATTNAHIITLGEPGTSPNTVFASNGLYFART
FHTSVVLPDGSTFITGGQRRGIPFEDSTPVFTPEIYVPEQDTFYKQNPNSIVRAYHSISLLLPDGRV
FNGGGGLCGDCTTNHFDAQIFTPNYLYDSNGNLATRPKITRTSTQSVKVGGRITISTDSSISKASL
IRYGTATHTVNTDQRRIPLTLTNNGGNSYSFQVPSDSGVALPGYWMLFVMNSAGVPSVASTIRV
TQGGGGSWSHPQFEK

pRed-14

MKKELGRINGKTQLIGLFATPIGHSLSPAMHNLAFKKLGLNYAYLAFEVGNEQLEDVVTGMRAL
NVRGFNVSMPNKMNILPYLDELADSAKFTGAVNTVVNENGRLIGHSTDGMGYVRNLKEHGVD
ITGKKMTLVGSGGAATPIAIQSALEGLGEISIFARNDAFFEKAEENVRIINEEMKGSSCKAKVFPLE
DQDALRAEIASSDIFTNGTGVGMKPLEGLSVIEDTSMFRPDLIVTDVVYNPVTSKLLEQAQAAG
CNTINGLGMMLWQGAMAFEYWTGREMPVQYIKEQMFE

pRed-15

MKIGFIGTGVMGKSMAGHLQKAGHQLFLYTRTKEKAEELLNKGAVWCKTPAEVARQTEVVFTI
VGEPHDVEEVYLGAEGILAGSSEGQMVIDMTTSQPSLARNIYEKAREKGVEALDAPVSGGDTGA
KNGTLAIMVGGSKAAFEKALPLFETIGSNIVYQGEAGAGQHTKMSNQIAIATNMIGVCEALLYA
QKAGLDLDNVLKSISTGAAGSWSLSNLAPRMIQGDFAPGFYVKHFIKDMKIAIEEAEKMGLELP
GLSLAKRMYEELAARGEEKSGTQALFNYWN

YdiB

MDVTAKYELIGLMAYPIRHSLSPEMQNKALEKAGLPFTYMAFEVDNDSFPGAIEGLKALKMRGT
GVSMPNKQLACEYVDELTPAAKLVGAINTIVNDDGYLRGYNTDGTGHIRAIKESGFDIKGKTMV
LLGAGGASTAIGAQGAIEGLKEIKLFNRRDEFFDKALAFAQRVNENTDCVVTVTDLADQQAFAE
ALASADILTNGTKVGMKPLENESLVNDISLLHPGLLVTECVYNPHMTKLLQQAQQAGCKTIDG
YGMLLWQGAEQFTLWTGKDFPLEYVKQVMGFGA

AroE

METYAVFGNPIAHSKSPFIHQQFAQQLNIEHPYGRVLAPINDFINTLNAFFSAGGKGANVTVPFK
EEAFARADELTERAALAGAVNTLMRLEDGRLLGDNTDGVGLLSDLERLSFIRPGLRILLIGAGGA
SRGVLLPLLSLDCAVTITNRTVSRAEELAKLFAHTGSIQALSMDELEGHEFDLIINATSSGISGDIP
AIPSSLIHPGIYCYDMFYQKGKTPFLAWCEQRGSKRNADGLGMLVAQAAHAFLLWHGVLPDV
EPVIKQLQEELSA

PtDH

MLPKLVITHRVHDEILQLLAPHCELMTNQTDSTLTREEILRRCRDAQAMMAFMPDRVDADFL
QACPELRVIGCALKGFDNFDVDACTARGVWLTFVPDLLTVPTAELAIGLAVGLGRHLRAADAF
VRSGKFRGWQPRFYGTGLDNATVGFLGMGAIGLAMADRLQGWGATLQYHEAKALDTQTEQR
LGLRQVACSELFASSDFILLALPLNADTLHLVNAELLALVRPGALLVNPCRGSVVDEAAVLAALE
RGQLGGYAADVFEMEDWARADRPQQIDPALLAHPNTLFTPHIGSAVRAVRLEIERCAAQNLIQ
ALAGERPINAVNRLPKAEPAAC

Reductase identification

Identification of new putative reductases

Multigeneblast searches were performed on two gene clusters that have been identified to produce iminosugars.[3–5] Four searches were carried out, two using the reported biosynthetic gene cluster sequences and two using those sequences followed by the sequence of a putative IRED from *B. Amyloli* and *C. pinensis*. In total, 45 potential reductases were identified, which were trimmed down to a final 15. A multiple sequence alignment was carried out and a phylogenetic tree was generated, demonstrating the broad scope of the enzymes mined. It was our intention to generate a very diverse panel of possible reductases, as many enzymes discovered that carry out imine reductions or reductive animations are often mislabelled or part of other superfamilies.[6] One of these 15 enzymes had previously been reported to catalyse the endocyclic imine reduction of a 2-methylpiperideine.[7] The sequence of 7 homologs were also disclosed as putative IREDs and so were also investigated for our cascade.

Once pRed-14 shikimate dehydrogenase had been identified as a positive hit in our cascade, a further 7 homologs of this protein were also identified and ordered for further testing.

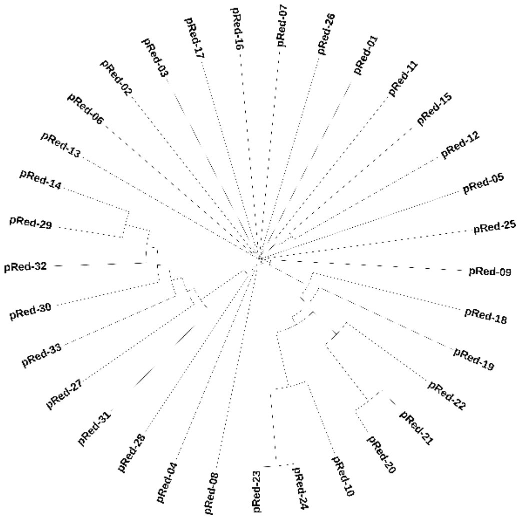

Figure S2: Phylogenetic tree of identified putative reductases through initial genome mining and subsequent homologs of pRed-14.

Colourimetric reductase screening

A colourimetric IRED screen was carried out in the oxidative direction as described in the literature using cis-3,4-dihydroxypiperidine as the substrate.[6] Assay plates were prepared using cell free extracts of the putative reductases identified in this work. Each well contained 0.25 mg mL^{-1} INT, 0.5 mM NAD(P)$^{+}$, 0.75 % v/v diaphorase, 4 mg mL^{-1} reductase lysate and 10 mM substrate, made up to a total volume of 200 µL with 100 mM Tris.HCl pH 9.0. Absorbance at 490 nm was measured before and after incubation at ambient temperature for 24h. After incubation, a deep red colour was observed in almost all wells including blanks and no meaningful conclusions could be drawn. Due to this and the lack of availability of the cascade intermediates, the reductases were screened in the context of the full cascade. When later validated in forward direction biotransformations, almost all of these results were shown to be false positives, i.e. not indicative of activity towards the desired imine reduction. Similar results have been observed with other iminosugar scaffolds and this colourimetric assay (data not shown here), possibly due to alcohol dehydrogenase activity present in endogenous enzymes within the lysate, or of the putative reductases.

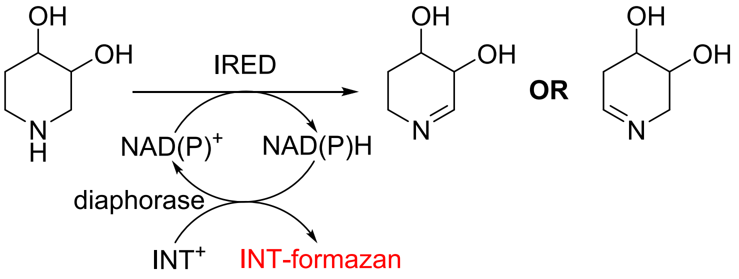

Scheme S1: Intended oxidative direction reaction of cis-3,4-dihydroxypiperidine used in the colourimetric screen of 23 putative reductases.

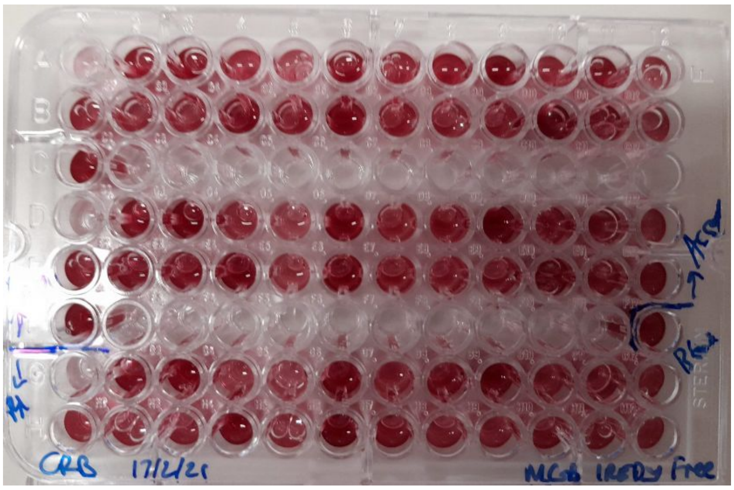

Figure S3: Colourimetric screen of 23 putative reductases in the oxidative direction, using cis-3,4-dihydroxypiperidine as the substrate.

Initial activity screening

Galactose oxidase liquid phase assay

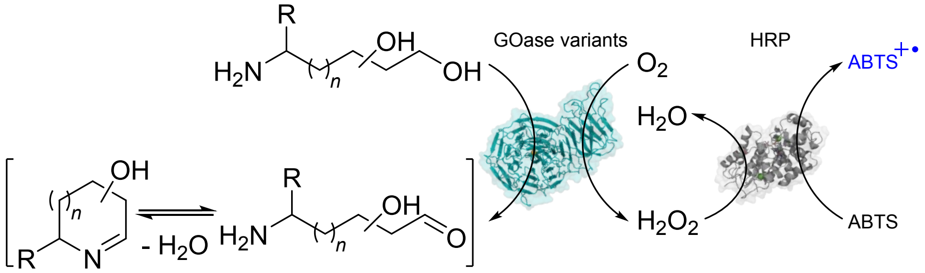

Scheme S2: Colourimetric HRP-ABTS assay used to determine activity of GOase variants.

GOase activity was initially determined by the well-known HRP-ABTS assay, where ABTS is reduced by HRP and H_2O_2 generated during oxidation of the substrate. [1,8] In triplicate, purified GOase enzyme (10 µL, 0.4–0.5 mg mL^{-1} stock) was supplemented with 90 µL of a reaction mix containing HRP (0.23 mg mL-1) and ABTS (0.4 mg mL^{-1}) in NaPi buffer (100 mM) in a 96 well plate. The assay reaction was initiated by addition of a substrate solution (100 µL, 50 mM in NaPi buffer) and absorbance was measured at 420 nm for 10 minutes and specific activities were calculated in µmol $min^{-1}mg^{-1}$ using the following formula.

$$specific\ activity = \frac{\Delta E \cdot v_{total}}{z \cdot \varepsilon \cdot v_{enzyme} \cdot c_{enzyme} \cdot d}$$

Where $v_{total} = 200\ \mu L$, $\varepsilon_{420\ nm} = 36\ mM^{-1}cm^{-1}$, $v_{enzyme} = 10\ \mu L$, $d = 0.55\ cm$ (depth of liquid in the well), $c_{enzyme} =$ final enzyme concentration, and ΔE is the initial rate of change in absorbance at 420 nm. The oxidation of 1 mol alcohol produces 1 mol of H_2O_2 but releases 2 mol e^- which reduce 2 mol ABTS, therefore $z = 2$.

Table S4: Specific activity of GOase variants against the aminopolyol substrate panel, measured using the above procedure. n.t. = not tested, blank entries where no activity was observed.

Substrate	GOase variant specific activity / µmol $min^{-1}mg^{-1}$		
	M_1	M_{3-5}	F_2
1-amino-2-deoxy-D-ribitol (**1**)			0.14
1-amino-D-xylitol (**2**)			
1-amino-D-arabinitol (**3**)			0.066
D-glucosaminitol (**4**)	0.017		0.41
D-galactosaminitol (**5**)	0.22	0.023	0.16
1-amino-D-glucitol (**6**)			0.25
1-methylamino-D-glucitol (**7**)		0.023	0.17
D-Glucose	n.t.	n.t.	1.01
D-Xylose	n.t.	n.t.	0.069

Assay to determine inhibition of GOase by iminosugar products

The HRP-ABTS assay was also used to determine if the iminosugar products formed in our cascade would inhibit GOase F_2. The assay was carried out as above, using 1,2,4-butanetriol and glycerol as model substrates and adding increasing concentrations of cis-3,4-dihydroxy piperidine to the reaction mix. Calculation of specific activities showed no significant reduction in activity in the presence of cis-3,4-dihydroxy piperidine. Using the same assay, it was determined that GOase F_2 exhibits no activity for cis-3,4-dihydroxy piperidine.

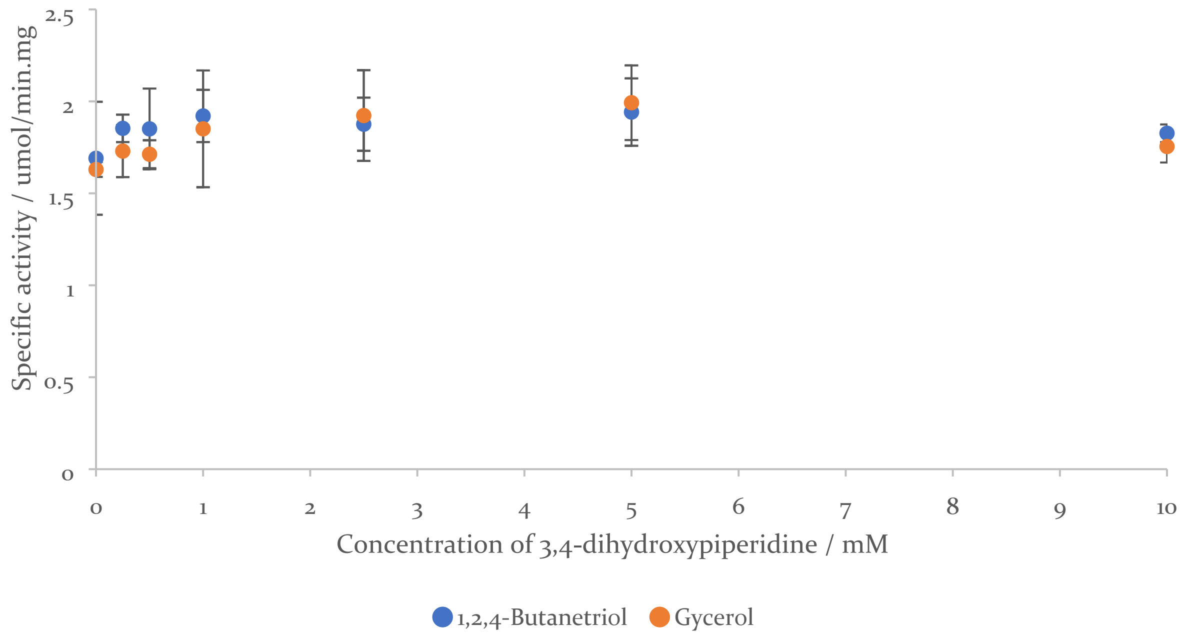

Figure S4: Specific activities of GOase F_2 against 1,2,4-butane triol and glycerol with increasing concentration of cis-3,4-dihydroxy piperidine.

Biotransformation Methods

Chemoenzymatic GOase-$NaCNBH_3$ cascade biotransformations

Reactions were carried out in 2 mL Eppendorf tubes with a total reaction volume of 250 µL. Each reaction contained components diluted from stock solutions, with final concentrations of 5 mM amino alcohol substrate, 1 mg ml^{-1} purified GOase, 0.1 mg ml^{-1} catalase, 0.1 mg ml^{-1} HRP, in 100 mM NaPi pH 7.4. Reactions were incubated at 30 °C, 200 rpm for 6 h followed by addition of $NaCNBH_3$ (25 mM, 5 equiv.) and incubation at 30 °C, 200 rpm for 16 h. After incubation, the biotransformations were quenched with an equal volume of MeOH and centrifuged (13k rpm, 10 minutes). The supernatant was decanted and derivatised using the AQC-tag procedure outlined above for analysis by reverse phase UPLC-QDA.

Screening of proposed GOase-Reductase cascade

1-amino-2-deoxy-D-ribose was used as a model substrate for investigations into the proposed GOase-reductase cascade.

Screening of 23 putative reductases as cell-free extracts in full cascade

Initial activity screening was carried out as one-pot simultaneous addition biotransformations. Reaction mixes were prepared in NaPi buffer (100 mM, pH 7.4) at a total volume of 250 µL and contained; purified GOase F_2 (0.5 mg mL^{-1}), HRP (0.1 mg mL^{-1}), catalase (0.1 mg mL^{-1}), putative reductase lysate (7.5 mg mL^{-1}), GDH (0.2 mg mL^{-1}), Glucose (25 mM), $NAD(P)^+$ (0.5 mM) and 1-amino-2-deoxy-D-ribose (5 mM). Biotransformations were incubated at 30 °C for 24 h, then quenched with methanol (50 µL) and centrifuged (13k rpm, 10 min) before derivatisation with the AQC-tag.

UPLC-QDA analysis of the tagged reaction mixtures revealed low levels of product formation (< 5%) with two of the enzymes tested. Several side product peaks were also observed and could be due to GOase catalysed oxidation of the glucose present in the reaction mix or side reactions of endogenous proteins remaining in the cell free extract. As such, further screening reactions were carried out using purified protein and stoichiometric NAD(P)H instead of $NAD(P)^+$ recycling.

Screening reactions with purified putative reductase

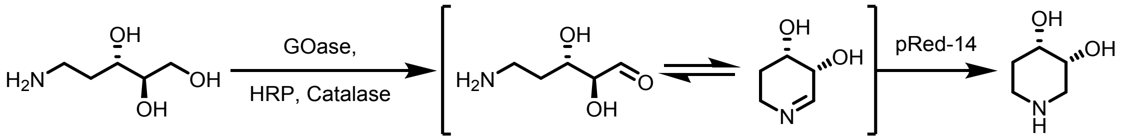

Scheme S3: Biocatalytic GOase-pRed cascade with 1-amino-2-deoxy-D-ribitol.

One-pot biotransformations were carried out with either simultaneous or sequential addition of GOase and Reductase components. Reaction mixes were prepared in NaPi buffer (100 mM, pH 7.4) at a total volume of 250 µL and typically contained; purified GOase F_2 (1 mg mL^{-1}), HRP (0.1 mg mL^{-1}), catalase (0.1 mg mL^{-1}), and 1-amino-2-deoxy-D-ribose (5 mM). Simultaneous addition biotransformations were supplemented with purified pRed enzyme (1 mg mL^{-1} final) and NAD(P)H (1 equiv., 5 mM) before incubation at 25–30 °C for 16 hours. In sequential addition biotransformations, the GOase reaction was incubated for 3–6 hours before addition of purified pRed enzyme (1 mg mL^{-1} final) and NADH (1 equiv., 5 mM) and incubation at 25–30 °C for 16

hours. After incubation, biotransformations were quenched with methanol (250 µL) and centrifuged (13k rpm, 10 min) before derivatisation with the AQC-tag and UPLC-QDA analysis.

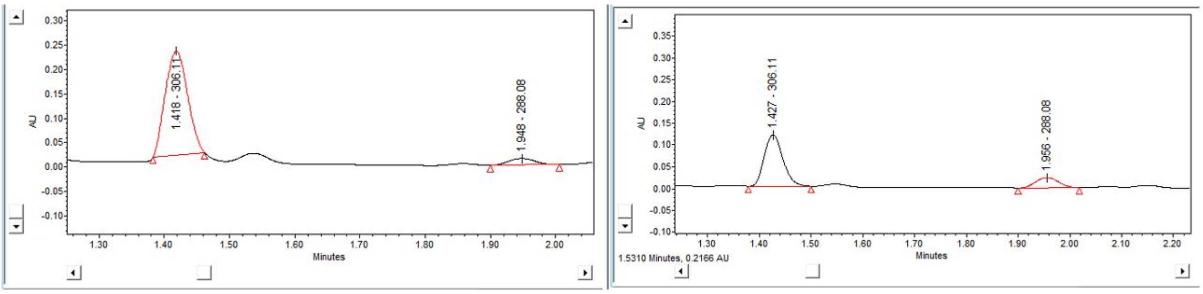

Figure S5: Reverse phase UPLC-QDA traces of biotransformations with purified pRed-14 and 1-amino-2-deoxy-ribitol. Left: simultaneous addition, 7% product formation. Right: sequential addition of pRed/co-factor after 3 h,19% product formation.

Reactions were also performed with GOase F_2 immobilised on purolite ECR 8285 epoxy resin, prepared as described in the literature.[1] Biotransformations with immobilised GOase were carried out with sequential addition of GOase and Reductase components. Reaction mixes were prepared in NaPi buffer (100 mM, pH 7.4) at a total volume of 500 µL and typically contained; Immobilised GOase F_2 (10–20 mg, 10 % w/w enzyme loading), HRP (0.1 mg mL^{-1}), catalase (0.1 mg mL^{-1}), and 1-amino-2-deoxy-D-ribose **1** (5 mM). The reaction mixture was incubated for 3–6 hours before addition of purified pRed enzyme (1 mg mL^{-1} final) and NAD(P)H (1 equiv., 5 mM) and incubation at 25–30 °C for 16 hours. After incubation, the supernatant was decanted and quenched with methanol (500 µL) and centrifuged (13k rpm, 10 min) before derivatisation with the AQC-tag and UPLC-QDA analysis.

Optimisation of GOase-pRed cascade with 1-amino-2-deoxy-D-ribitol

Biotransformations were performed in one-pot sequential addition mode and reaction parameters were varied as follows. Reaction mixes were prepared in NaPi buffer (100 mM, pH 7–8) at a total volume of 250 µL and typically contained; purified GOase F_2 (1 mg mL^{-1}), HRP (0.1 mg mL^{-1}), catalase (0.1 mg mL^{-1}), and 1-amino-2-deoxy-D-ribose **1** (5–50 mM). The reaction was incubated for 4 hours before addition of purified pRed enzyme (1 mg mL^{-1} final) and NADH (1 equiv., 5 mM) and incubation at 25–30 °C for 16 hours. After incubation, biotransformations were quenched with methanol (250 µL) and centrifuged (13k rpm, 10 min) before derivatisation with the AQC-tag and UPLC-QDA analysis.

Investigations into reaction pH

Reactions were performed as above in various pH of NaPi buffer either from start to finish or by beginning the reaction at one pH and adjusting upon addition of the reductase components. Reactions were incubated at 30 °C. The following data shows that the one-pot cascade was most efficient when pH was maintained at 8.0 throughout.

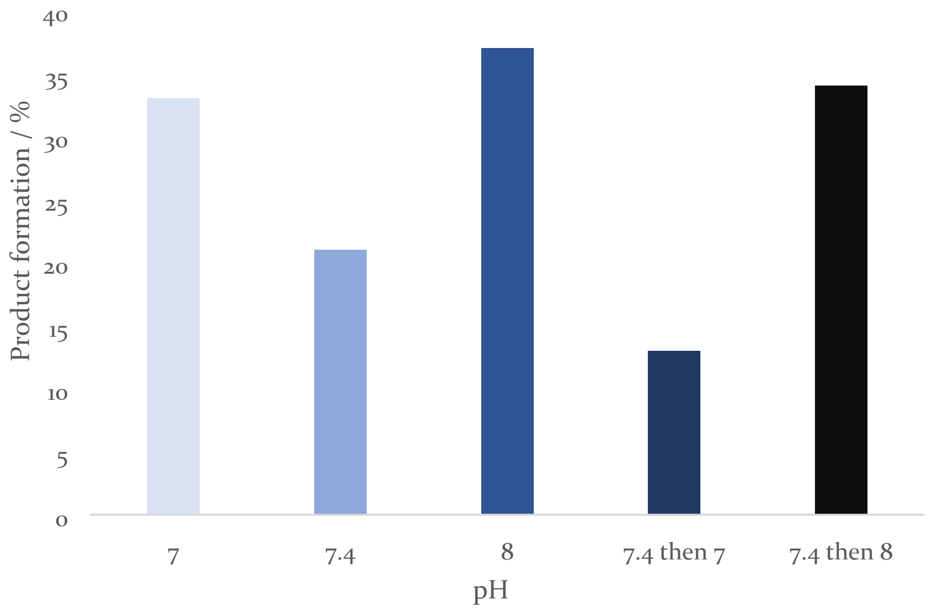

Figure S6: Influence of buffer pH on formation of product 3,4-dihydroxypiperidine **1a** from 1-amino-2-deoxy-D-ribitol **1** (5 mM final) in the GOase-pRed-14 cascade as determined by RP-UPLC-QDA.

Investigations into reaction temperature

Reactions were performed as above (in NaPi buffer pH 7.4) with either 5 or 10mM substrate and incubated at 25 or 30 °C. It was observed that the lower reaction temperature improved overall product formation, likely due to an improvement in enzyme stability. A small drop in formation of product was observed from 5 to 10 mM substrate at both temperatures.

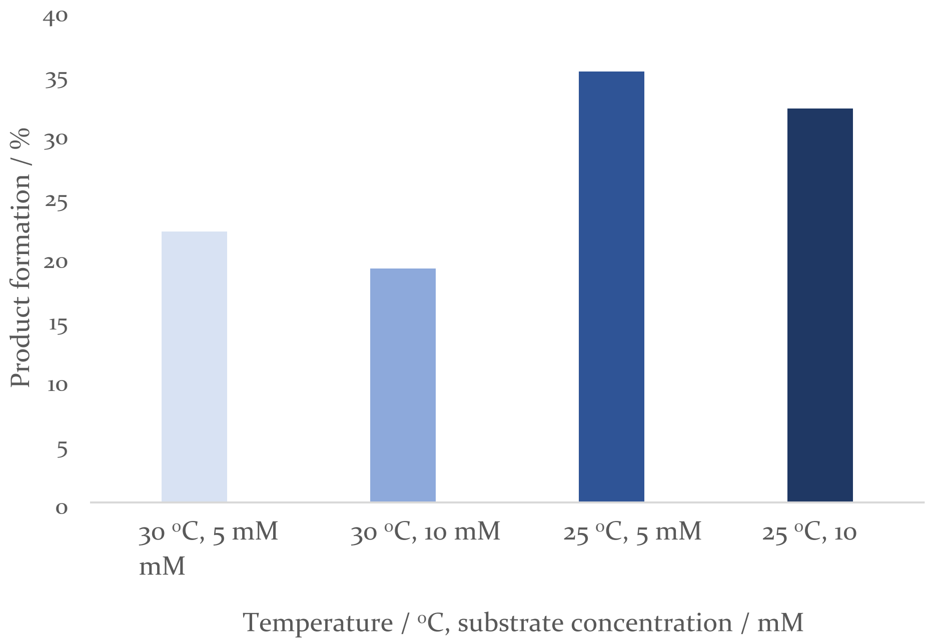

Figure S7: Influenc[illegible]ypiperidine **1a** from 1-amino-2-deoxy-D-ribitol **1** (5 mM final) in the GOase-pRed-14 cascade at two different substrate concentrations as determined by RP-UPLC-QDA

Investigations into substrate loading

Reactions were performed as above using a range of substrate concentrations with both free and immobilised GOase F_2 in NaPi (100 mM, pH 8.0), incubated at 25 °C. Generally, as substrate concentrations increased the formation of product decreased, although for immobilised GOase a higher product formation was seen for 25 mM substrate than with 10 mM. A large decrease in product formation was observed upon increasing substrate concentration to 50 mM with both free and immobilised GOase (Figure S7 & S8, respectively).

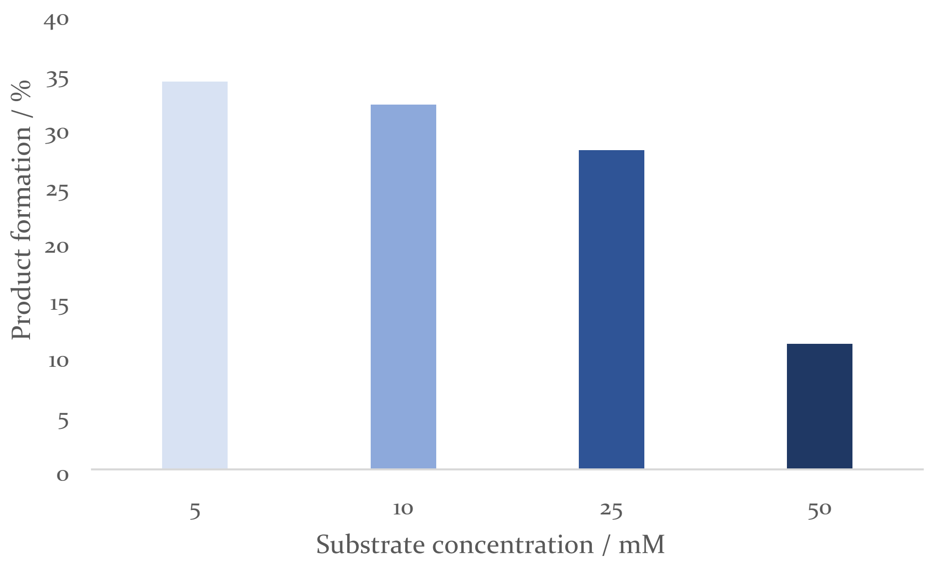

Figure S8: Influence of substrate concentration on formation of product 3,4-dihydroxypiperidine **1a** from 1-amino-2-deoxy-D-ribitol **1** in the GOase-pRed-14 cascade with free GOase as determined by RP-UPLC-QDA

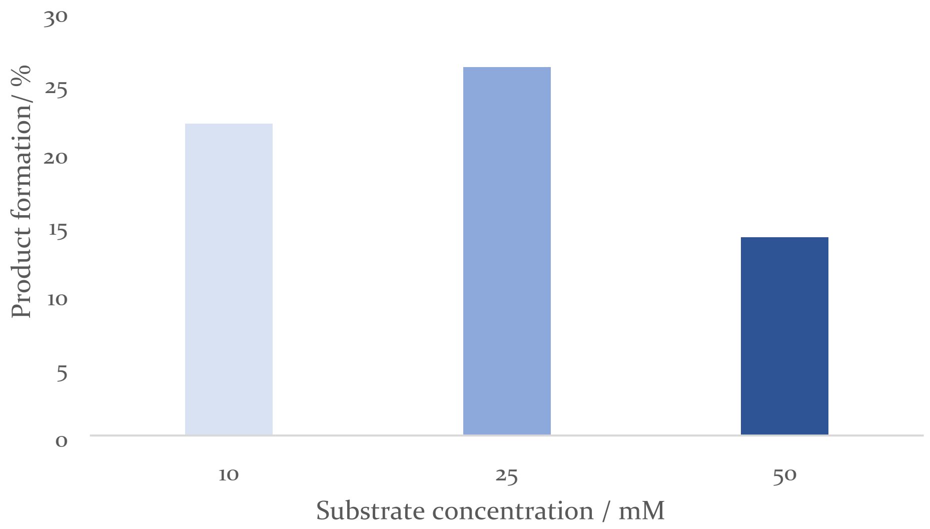

Figure S9: Influence of substrate concentration on formation of product 3,4-dihydroxypiperidine **1a** from 1-amino-2-deoxy-D-ribitol **1** in the GOase-pRed-14 cascade with immobilised GOase as determined by RP-UPLC-QDA

Implementation of NAD$^+$ co-factor recycling

Reactions were performed as above using free GOase F_2 and 10 mM substrate in NaPi buffer pH 8.0, incubated at 25 °C. Biotransformations were performed in one-pot sequential or simultaneous addition mode and reaction parameters were varied as follows. Reaction mixes were prepared in NaPi buffer (100 mM, pH 8) at a total volume of 250 µL and typically contained; purified GOase F_2 (1 mg mL^{-1}), HRP (0.1 mg mL^{-1}), catalase (0.1 mg mL^{-1}), and 1-amino-2-deoxy-D-ribose (10 mM). The reaction was incubated for 4 hours before addition of purified pRed enzyme (1 mg mL^{-1} final) and either **a)** NADH (10 mM); **b)** NAD$^+$ (0.5 mM), PtDH lysate (0.2 mg mL^{-1}) and NaPt (50 mM final); or **c)** NAD$^+$ (0.5 mM), GDH lysate (0.2 mg mL^{-1}) and D-glucose (50 mM final). Biotransformations were incubated at 25 °C for 16 hours, quenched with methanol (250 µL) and centrifuged (13k rpm, 10 min) before derivatisation with the AQC-tag and UPLC-QDA analysis.

Pleasingly, implementation of either GDH or PtDH recycling systems improved product formation when compared to stoichiometric NADH. The slightly better performance of PtDH recycling system may relate to elimination of glucose in the reaction mixture acting as a competing substrate for GOase F_2, thus reducing side reactions and GOase deactivation. Negative control reactions containing no pRed-14 were also performed and showed that the GDH/ PtDH recycling enzymes did not provide any product formation.

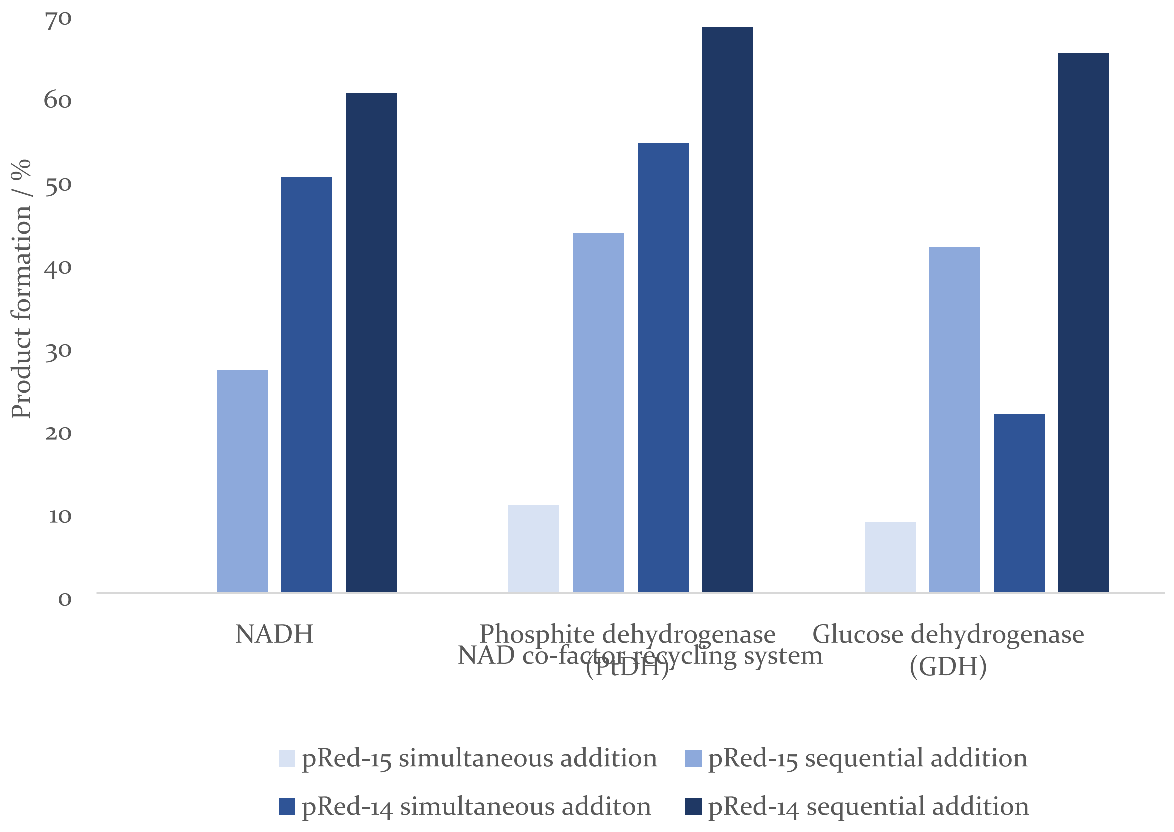

Figure S10: Influence of recycling system on formation of product 3,4-dihydroxypiperidine **1a** from 1-amino-2-deoxy-D-ribitol **1** in the GOase-pRed-14 cascade as determined by RP-UPLC-QDA

Optimisation of NAD^+ co-factor recycling

Reactions were performed as above using free GOase F_2 and 10 mM substrate in NaPi buffer pH 8.0, incubated at 25 °C. Biotransformations were performed in one-pot sequential addition mode and reaction parameters were varied as follows. Reaction mixes were prepared in NaPi buffer (10–100 mM, pH 8) at a total volume of 250 µL and typically contained; purified GOase F_2 (1 mg mL^{-1}), HRP (0.1 mg mL^{-1}), catalase (0.1 mg mL^{-1}), and 1-amino-2-deoxy-D-ribose (10 mM). The reaction was incubated for 4 hours before addition of purified pRed enzyme (0.25–1 mg mL^{-1} final), NAD (0.5 mM), PtDH lysate (0.05–0.3 mg mL^{-1}) and NaPt (10–75 mM final) and incubation at 25 °C for 16 hours. After incubation, biotransformations were quenched with methanol (250 µL) and centrifuged (13k rpm, 10 min) before derivatisation with the AQC-tag and UPLC-QDA analysis.

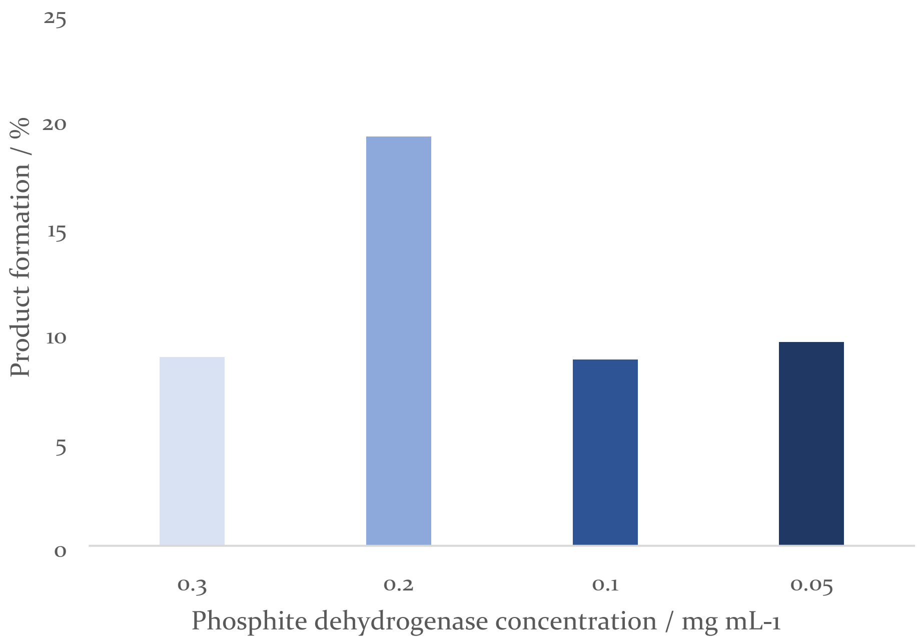

Figure S11: Influence of PtDH concentration on formation of product 3,4-dihydroxypiperidine **1a** from 1-amino-2-deoxy-D-ribitol **1** in the GOase-pRed-14 cascade as determined by RP-UPLC-QDA

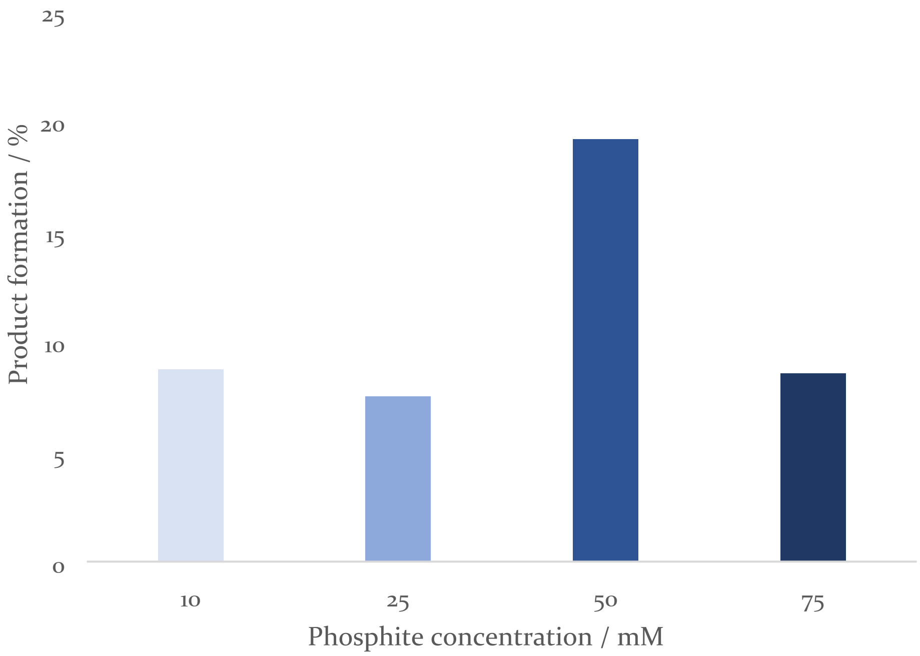

Figure S12: Influence of phosphite concentration on formation of product 3,4-dihydroxypiperidine **1a** from 1-amino-2-deoxy-D-ribitol **1** in the GOase-pRed-14 cascade as determined by RP-UPLC-QDA

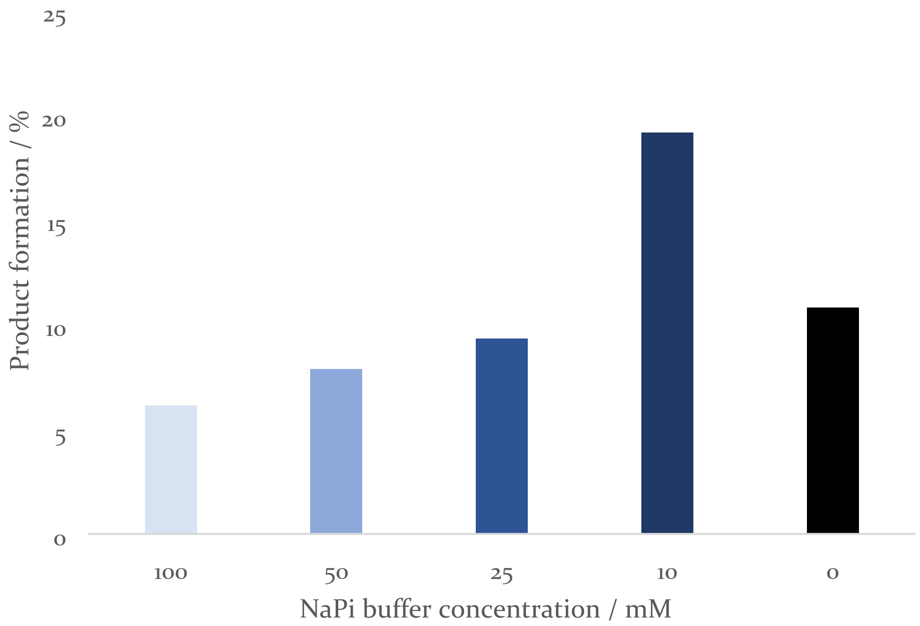

Figure S13: Influence of NaPi buffer concentration on formation of product 3,4-dihydroxypiperidine **1a** from 1-amino-2-deoxy-D-ribitol **1** in the GOase-pRed-14 cascade as determined by RP-UPLC-QDA

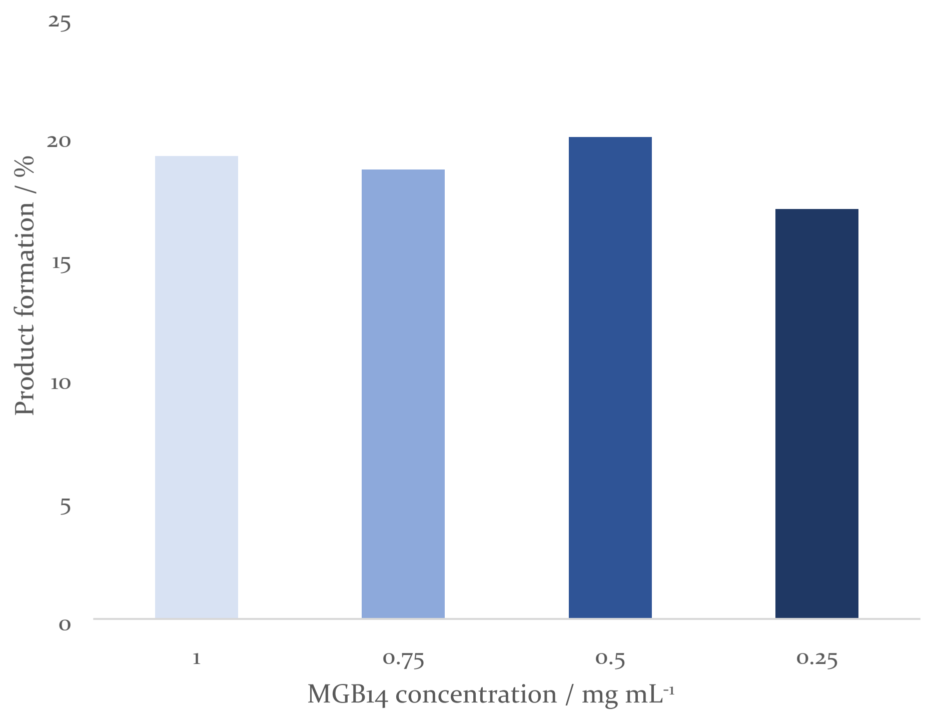

Figure S14: Influence of pRed-14 concentration on formation of product 3,4-dihydroxypiperidine **1a** from 1-amino-2-deoxy-D-ribitol **1** in the GOase-pRed-14 cascade as determined by RP-UPLC-QDa

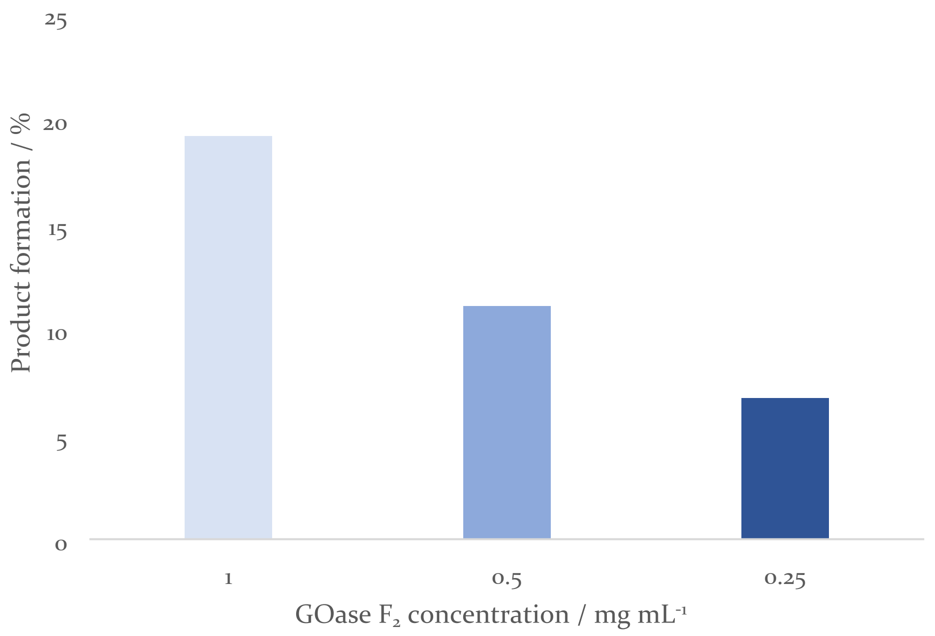

Figure S15: Influence of free GOase F_2 concentration on formation of product 3,4-dihydroxypiperidine **1a** from 1-amino-2-deoxy-D-ribitol **1** in the GOase-MGB14 cascade as determined by RP-UPLC-QDa

Substrate scope of the biocatalytic and chemoenzymatic cascades

General procedure for biotransformations & reaction purification

Biotransformations were performed in one-pot sequential addition mode with the optimised conditions. Reaction mixes were prepared in NaPi buffer (10 mM, pH 8) at a total volume of 250 µL and contained; purified GOase F_2 (1 mg mL^{-1}), HRP (0.1 mg mL^{-1}), catalase (0.1 mg mL^{-1}), and substrate **1–7** (10 mM). The reaction was incubated for 6 hours before addition of purified reductase enzyme (0.5 mg mL^{-1} final), NAD^+ (0.5 mM), PtDH lysate (0.2 mg mL^{-1}) and NaPt (50 mM final from a 1M stock at pH 8.0) and incubation at 25 °C for 16 hours. After incubation, biotransformations were quenched with methanol (250 µL) and centrifuged (13k rpm, 10 min) before derivatisation with the AQC-tag and UPLC-QDa analysis. These reactions were performed with MGB14 and two homologs, YdiB and AroE shikimate dehydrogenases from *E. coli*.

Alternatively, reactions were performed with a chemical reducing agent in place of the reductase components. Reaction mixes were prepared in NaPi buffer (10 mM, pH 8) at a total volume of 250 µL and contained; purified GOase F_2 (1 mg mL^{-1}), HRP (0.1 mg mL^{-1}), catalase (0.1 mg mL^{-1}), and substrate **1–7** (10 mM). The reaction was incubated for 6 hours before addition of $NaCNBH_3$ (50 mM, 5 equiv.) and incubation at 25 °C for 16 hours. After incubation, biotransformations were quenched with methanol (250 µL) and centrifuged (13k rpm, 10 min) before derivatisation with the AQC-tag and UPLC-QDa analysis.

Select reactions were scaled up to 10 mL in order to characterise products by NMR.

Purification procedure:

Crude reaction mixtures with phosphate salts and protein removed were concentrated and resuspended in minimal H_2O then loaded onto a pre-washed column of Dowex 50WX8 H^+ or NH_4^+ form. The loaded column was washed once more with H_2O (3–5 cv.) and then fractions were collected with an increasing concentration of aqueous NH_3 (0–1 M, 50 mM increments, 3 cv. per solution). The fractions were analysed by TLC or UPLC-QDa, concentrated *in vacuo*, resuspended in 1M HCl and concentrated *in vacuo* once more. Using this approach it was possible to separate the linear amino alcohol substrate from the cyclic iminosugar product and obtain pure compound for characterisation. In select cases where fractions were mixed or not completely pure, the above procedure was applied again using a column of Dowex 50WX8 in NH_4^+ form.

Table S5: Substrate scope of the developed cascades with conditions optimised with substrate **1**. Product formation for **1a–6a** determined by UPLC-QDa peak area. Product formation of **7a**determined by ^{1}H-NMR. Blank cells indicate no product formation.

Substrate	Product	Product formation / %			
		pRed-14	YdiB	AroE	$NaCNBH_3$
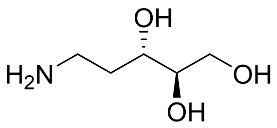 **1**	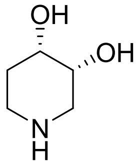 **1a**	88	43	68	85

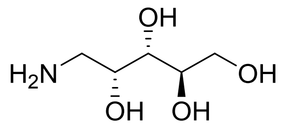 **3**	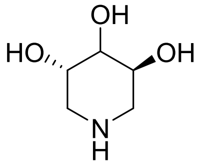 **3a**	80	64	79	53
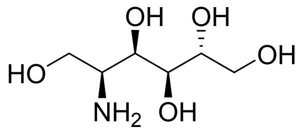 **4**	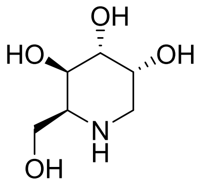 **4a**				95
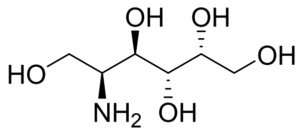 **5**	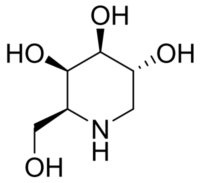 **5a**				32
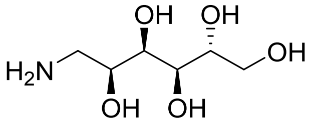 **6**	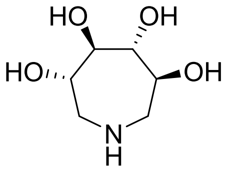 **6a**				98
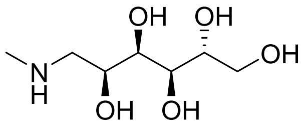 **7**	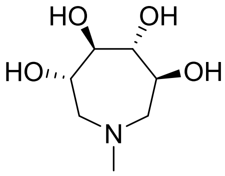 **7a**				99

Mechanistic insight into SDH catalysed reduction

Possible tautomerisation and amadori rearrangement pathways

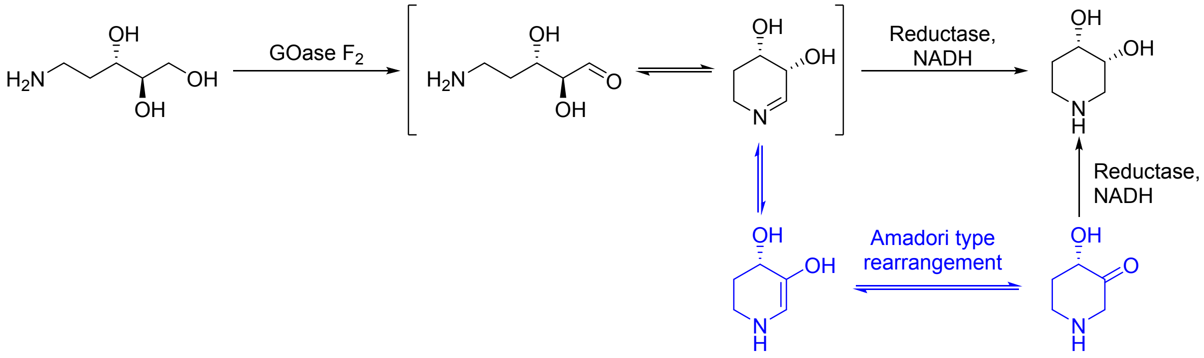

Scheme S4: Possible tautomerisation and amadori rearrangement pathway of the imine intermediate

Deuteration experiments used to investigate reduction mechanism

Biotransformations were performed in one-pot sequential addition mode with a deuterium based co-factor recycling system. Reaction mixes were prepared in H_2O or D_2O at a total volume of 1 mL and contained; GOase F_2 (20 mg, 10% w/w, immobilised on Purolite ECR8285 epoxy),

HRP (0.1 mg mL^{-1}), catalase (0.1 mg mL^{-1}), and substrate **1–7** (10 mM, from a stock in H_2O or D_2O). The reaction was incubated for 6 hours before addition of purified reductase enzyme (0.5 mg mL^{-1} final), NAD$^+$ (0.5 mM), CDX-901 GDH lysate (0.2 mg mL^{-1}) and D-glucose-d1 (50 mM final) and incubation at 25 °C for 16 hours. After incubation, biotransformations were centrifuged (13k rpm, 10 min) before derivatisation with the AQC-tag and UPLC-QDa analysis.

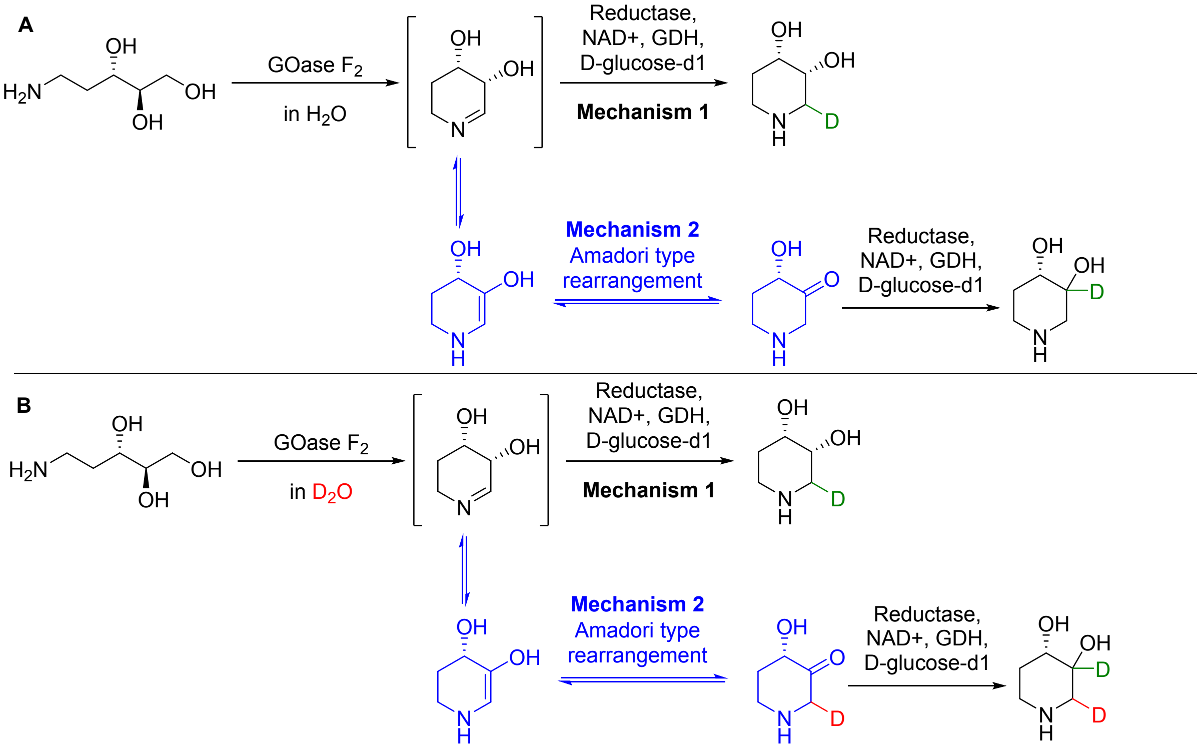

Scheme S5: Proposed major pathways of deuteration experiments to probe enzyme mechanism

UPLC-QDa analysis of biotransformation mixtures showed that shikimate dehydrogenases catalysed the transfer of deuterium from NADD generated *in situ* to the product, though no further mechanistic insight could be gained.

UPLC-QDa chromatograms

Substrates and standards

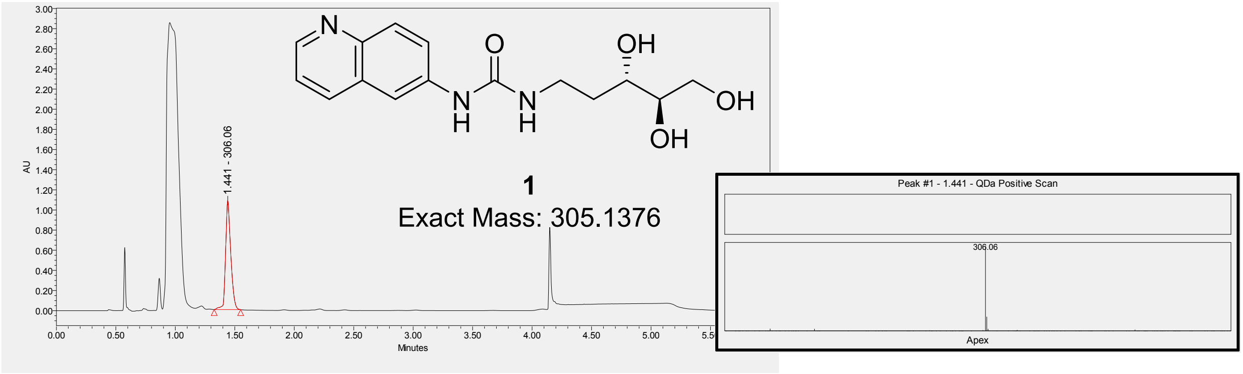

Figure S16: Reverse phase (C18) UPLC-QDa trace of AQC tagged 1-amino-2-deoxy-ribitol substrate standard. The peak at 0.95 minutes is a by-product of the AQC tagging reaction.

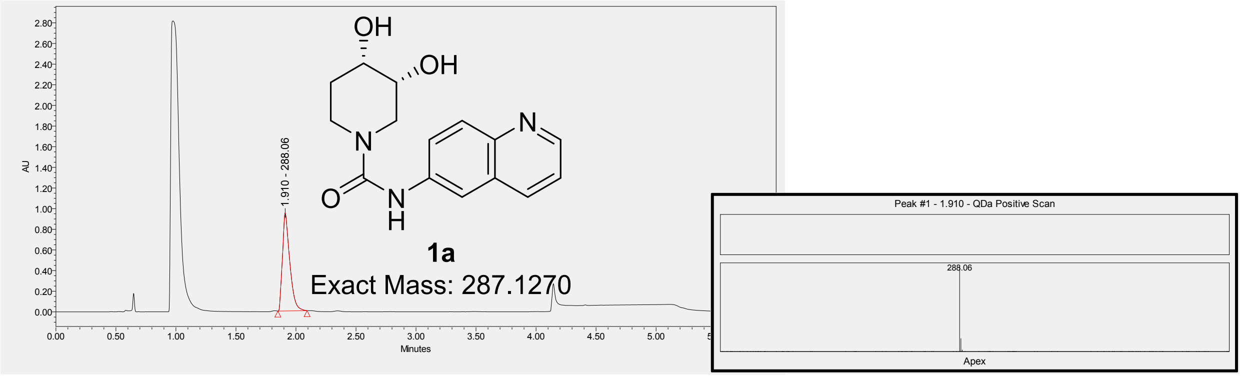

Figure S17: Reverse phase (C18) UPLC-QDa trace of AQC tagged cis-3,4-dihydroxypiperidine standard. The peak at 0.94 minutes is a by-product of the AQC tagging reaction.

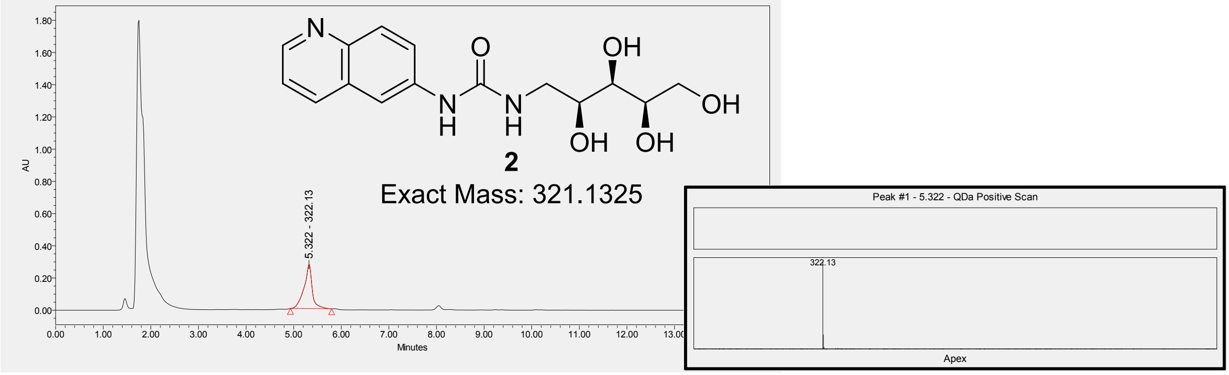

Figure S18: Normal phase HILIC-UPLC-QDa trace of AQC tagged 1-aminoxylitol substrate standard.

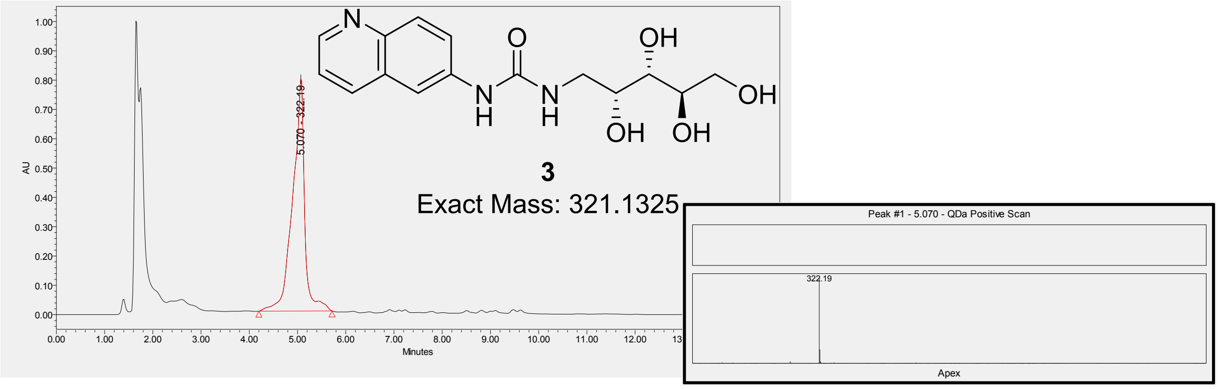

Figure S19: Normal phase HILIC-UPLC-QDa trace of AQC tagged 1-aminoarabinitol substrate standard.

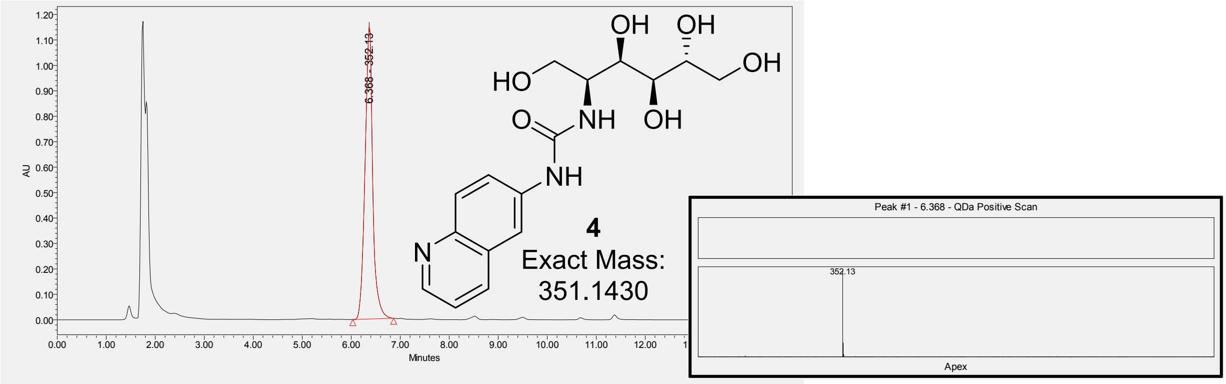

Figure S20: Normal phase HILIC-UPLC-QDa trace of AQC tagged glucosaminitol substrate standard.

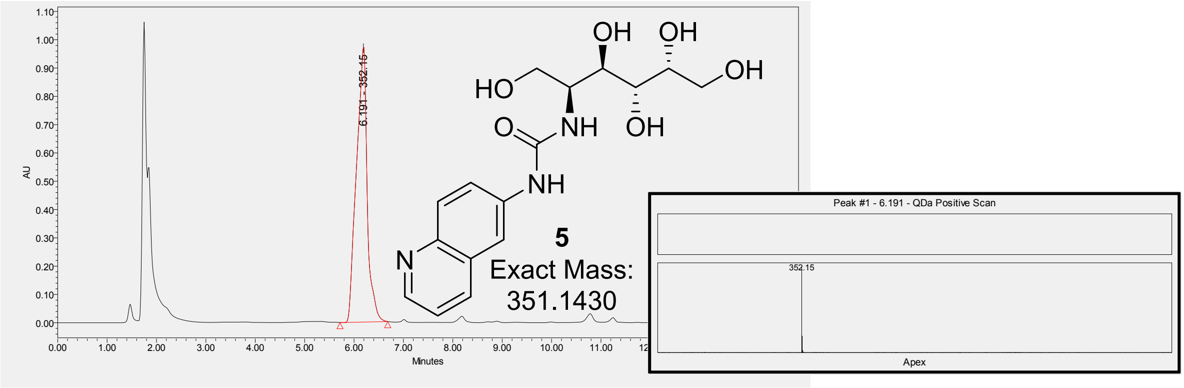

Figure S21: Normal phase HILIC-UPLC-QDa trace of AQC tagged galactosaminitol substrate standard.

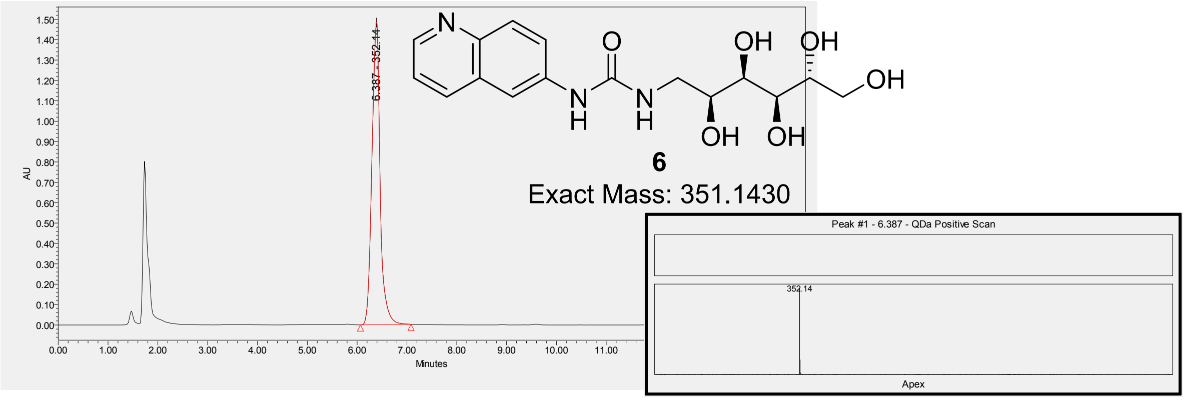

Figure S22: Normal phase HILIC-UPLC-QDa trace of AQC tagged glucamine substrate standard.

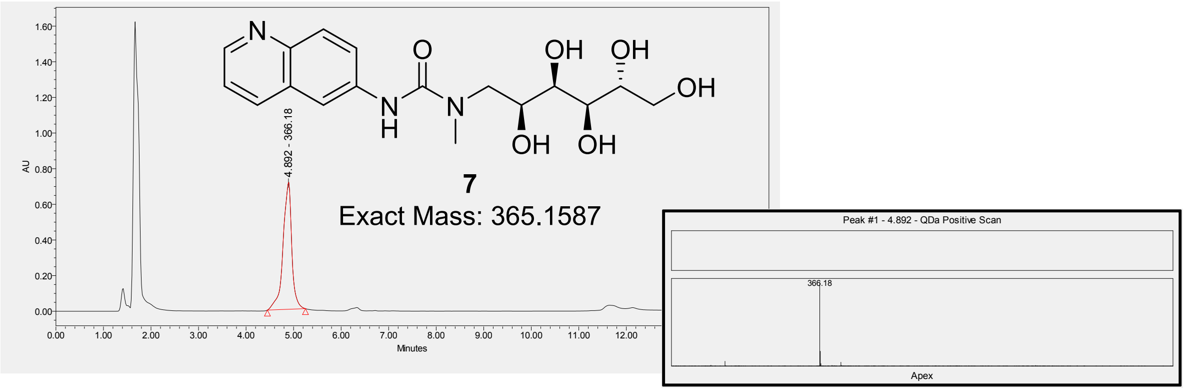

Figure S23: Normal phase HILIC-UPLC-QDa trace of AQC tagged *N*-methylglucamine substrate standard.

Biotransformations

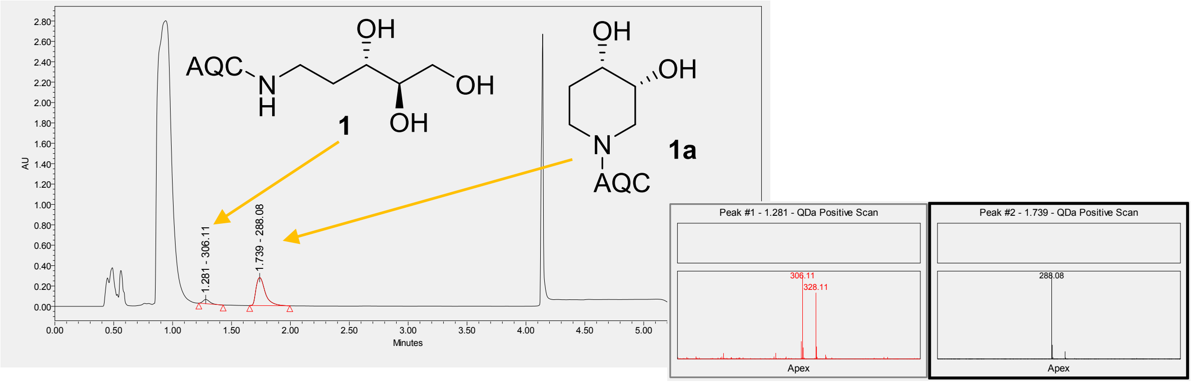

Figure S24: Example reverse phase (C18) UPLC-QDa trace of an AQC-tagged biotransformation with 1-amino-2-deoxy-ribitol, GOase F_2 and MGB14.

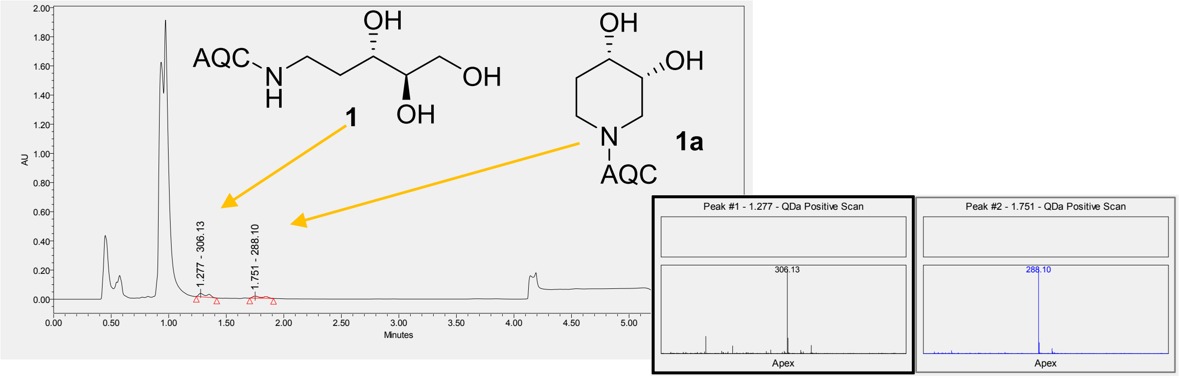

Figure S25: Example reverse phase (C18) UPLC-QDa trace of an AQC-tagged biotransformation with 1-amino-2-deoxy-ribitol **1**, GOase F_2 and YdiB.

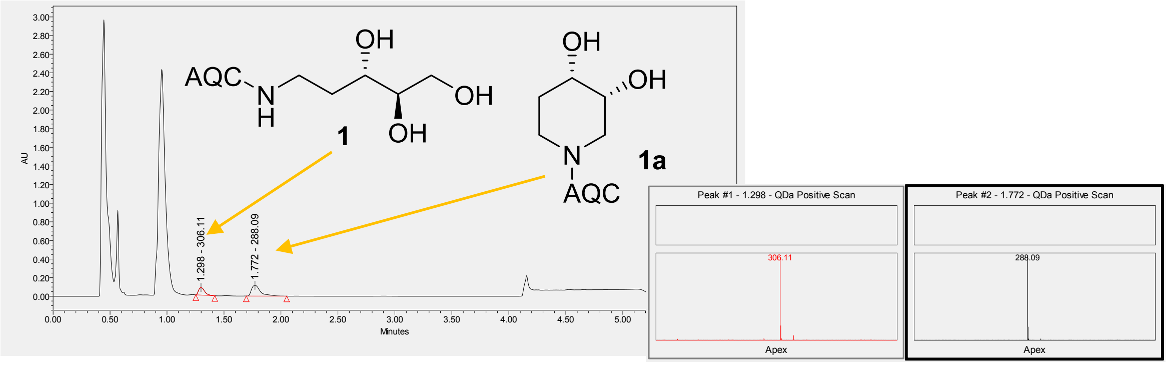

Figure S26: Example reverse phase (C18) UPLC-QDa trace of an AQC-tagged biotransformation with 1-amino-2-deoxy-ribitol **1**, GOase F_2 and AroE.

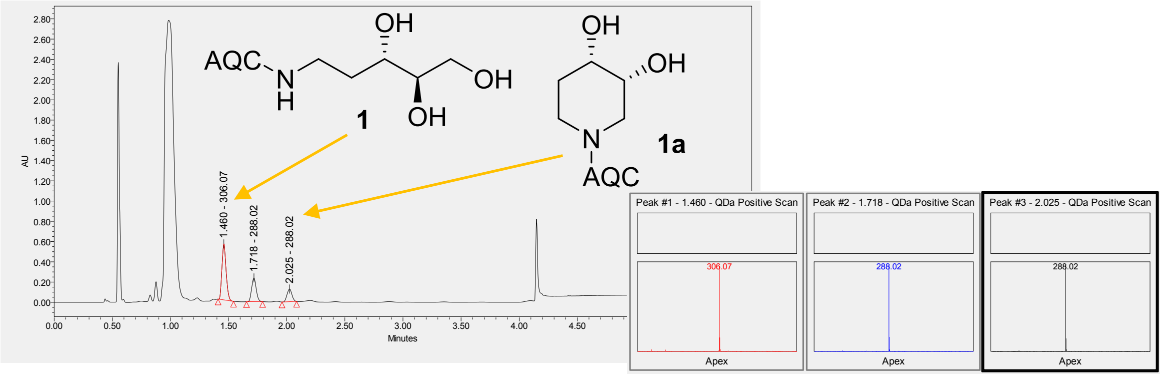

Figure S27: Example reverse phase (C18) UPLC-QDa trace of an AQC-tagged biotransformation with 1-amino-2-deoxy-ribitol **1**, GOase F_2 and $NaCNBH_3$. The by-product peak at 1.718 is assumed to be the 3,4-trans diol diastereomer of the piperidine product, which could be generated by non-selective reduction of a racemised intermediated generated through imine-enamine tautomerisation. Importantly, no such by-product peak is observed in any of the biocatalytic reductase reactions.

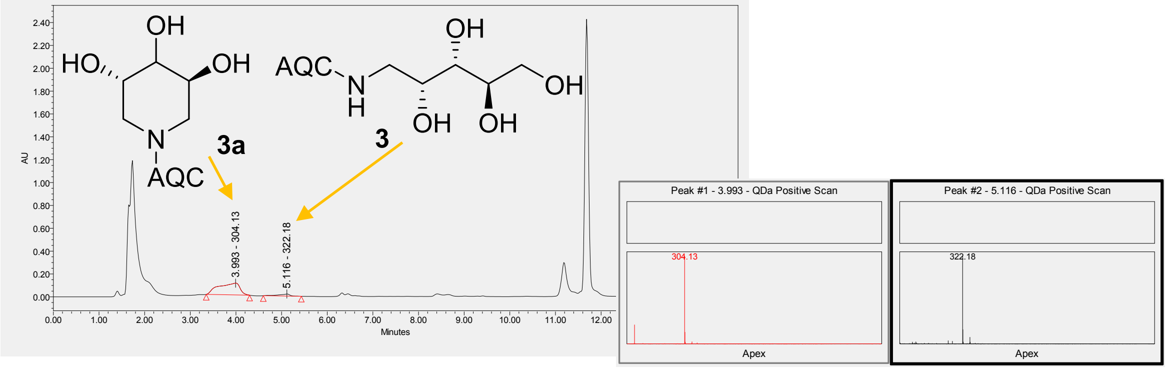

Figure S28: Example normal phase HILIC-UPLC-QDa trace of an AQC-tagged biotransformation with 1-aminoarabinitol **3**, GOase F_2 and MGB14.

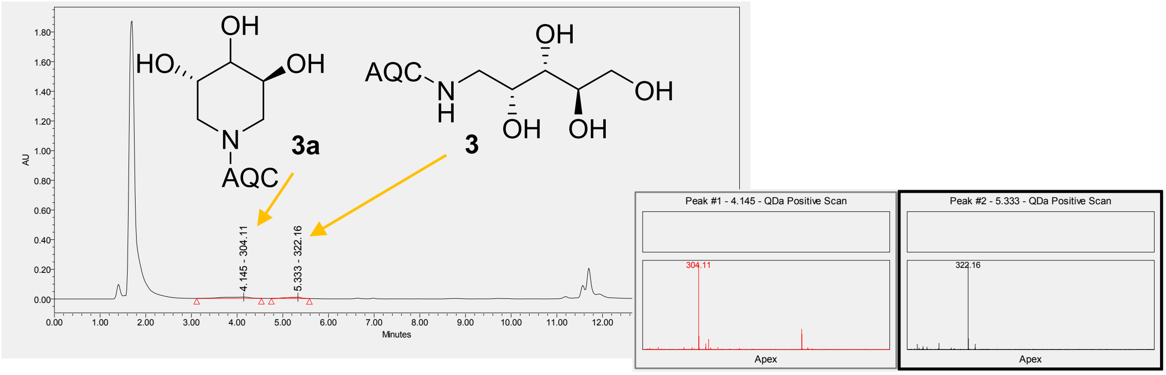

Figure S29: Example normal phase HILIC-UPLC-QDa trace of an AQC-tagged biotransformation with 1-aminoarabinitol **3**, GOase F_2 and YdiB.

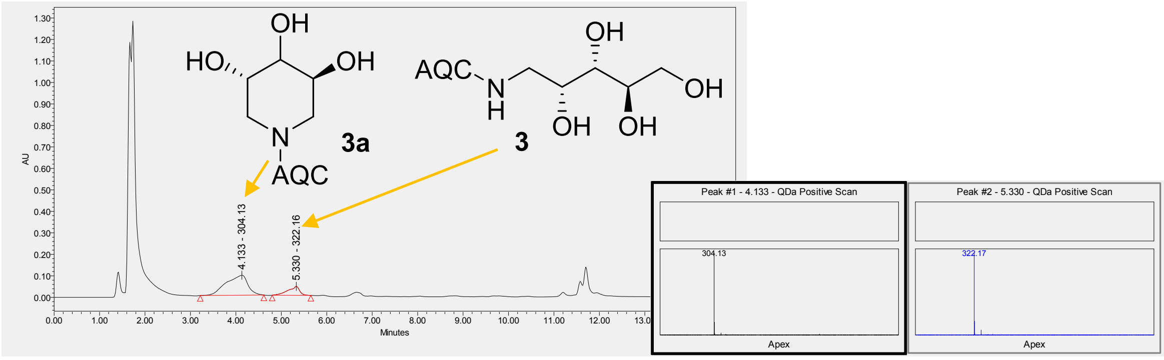

Figure S30: Example normal phase HILIC-UPLC-QDa trace of an AQC-tagged biotransformation with 1- aminoarabinitol **3**, GOase F_2 and AroE.

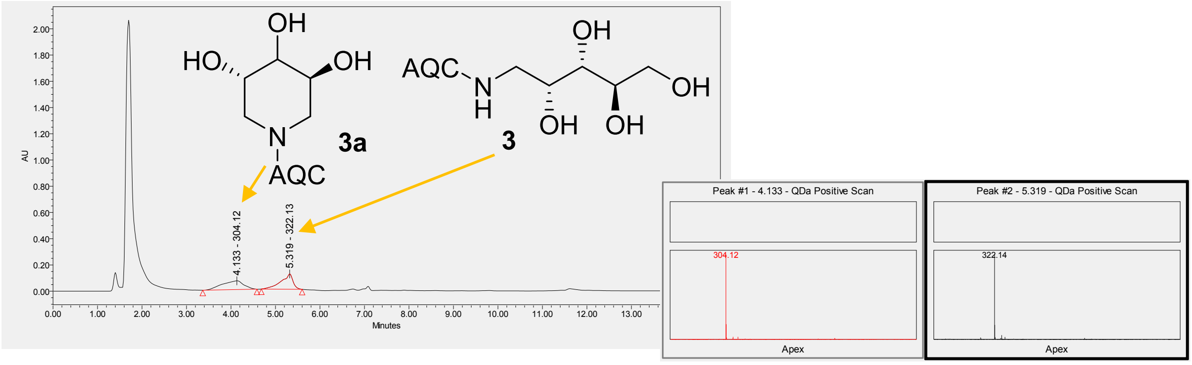

Figure S31: Example normal phase HILIC-UPLC-QDa trace of an AQC-tagged biotransformation with 1- aminoarabinitol **3**, GOase F_2 and $NaCNBH_3$.

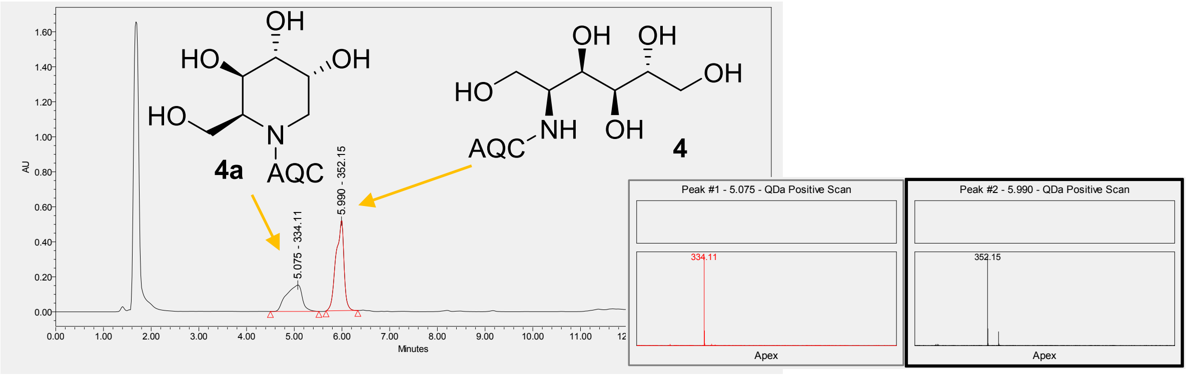

Figure S32: Example normal phase HILIC-UPLC-QDa trace of an AQC-tagged biotransformation with glucosaminitol **4**, GOase F_2 and $NaCNBH_3$.

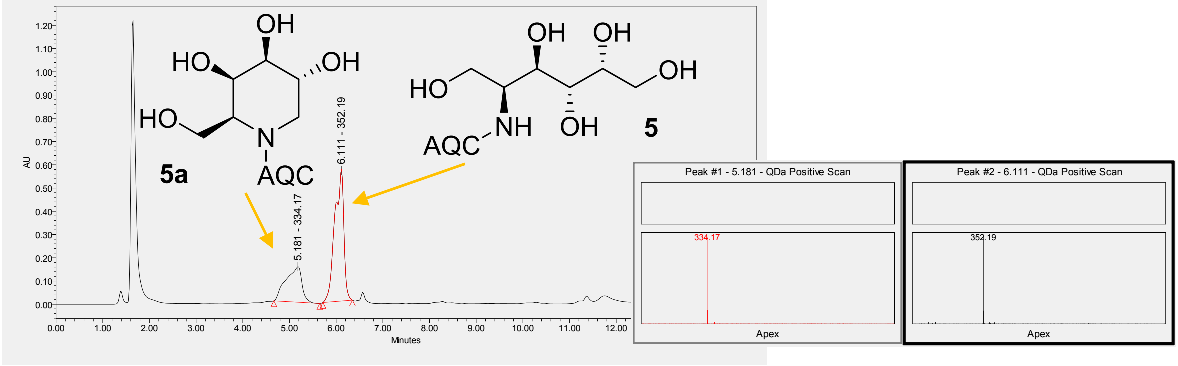

Figure S33: Example normal phase HILIC-UPLC-QDa trace of an AQC-tagged biotransformation with galactosaminitol **5**, GOase F_2 and $NaCNBH_3$.

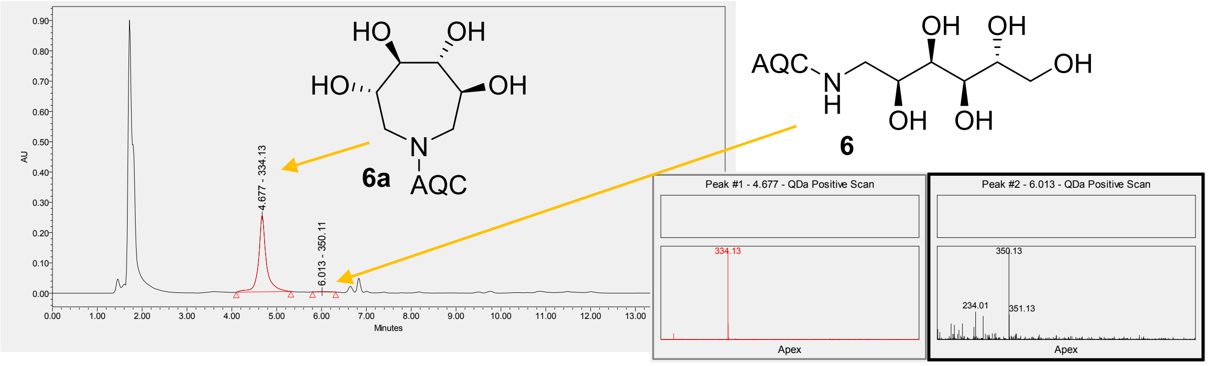

HILIC-UPLC-QDa trace of an AQC-tagged biotransformation with glucamine **6**, GOase F_2 and $NaCNBH_3$.

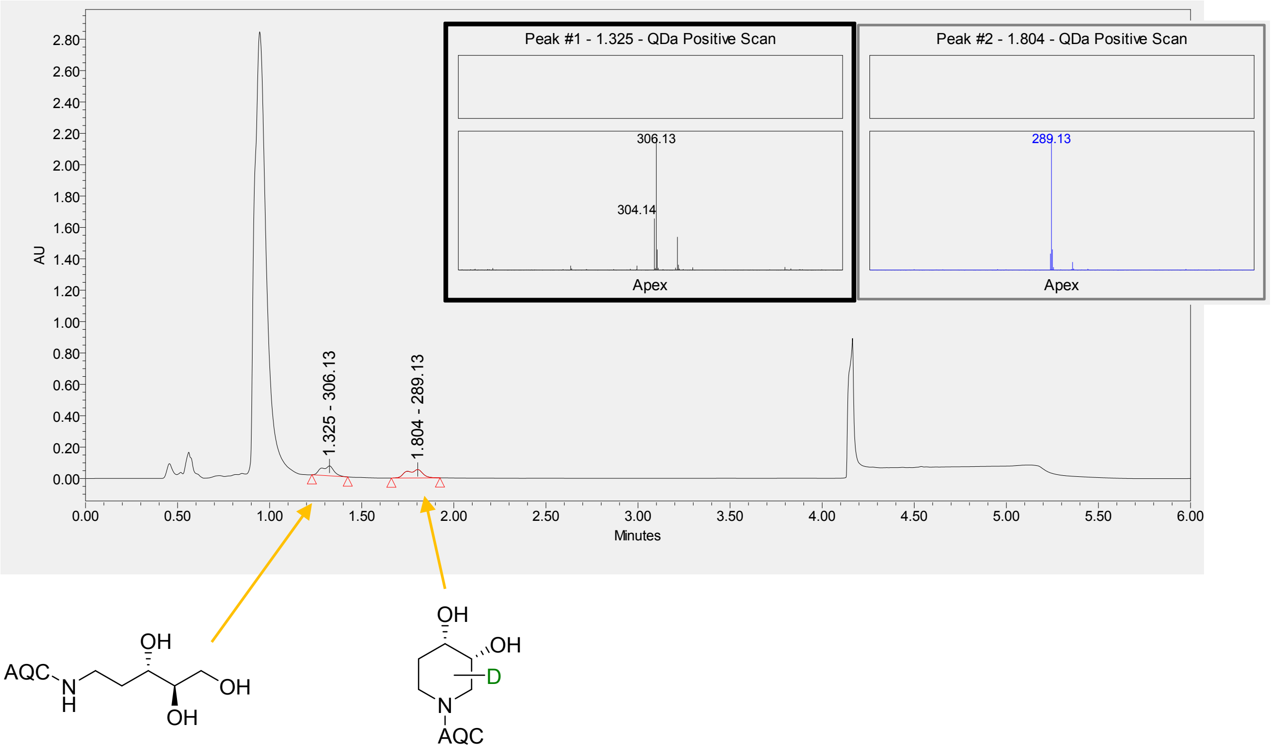

Figure S34: Reverse-phase C18 UPLC-QDa trace of an AQC-tagged biotransformation with substrate **1**, GOase F_2, pRed-14 and the deuterium based recycling system CDX-901 GDH/ D-glucose-1-d_1. Product *m/z* of 289 indicates increase of 1*m/z* from analogous reaction with hydride recycling, corresponding to inclusion of deuterium.

Determination of product formation by NMR

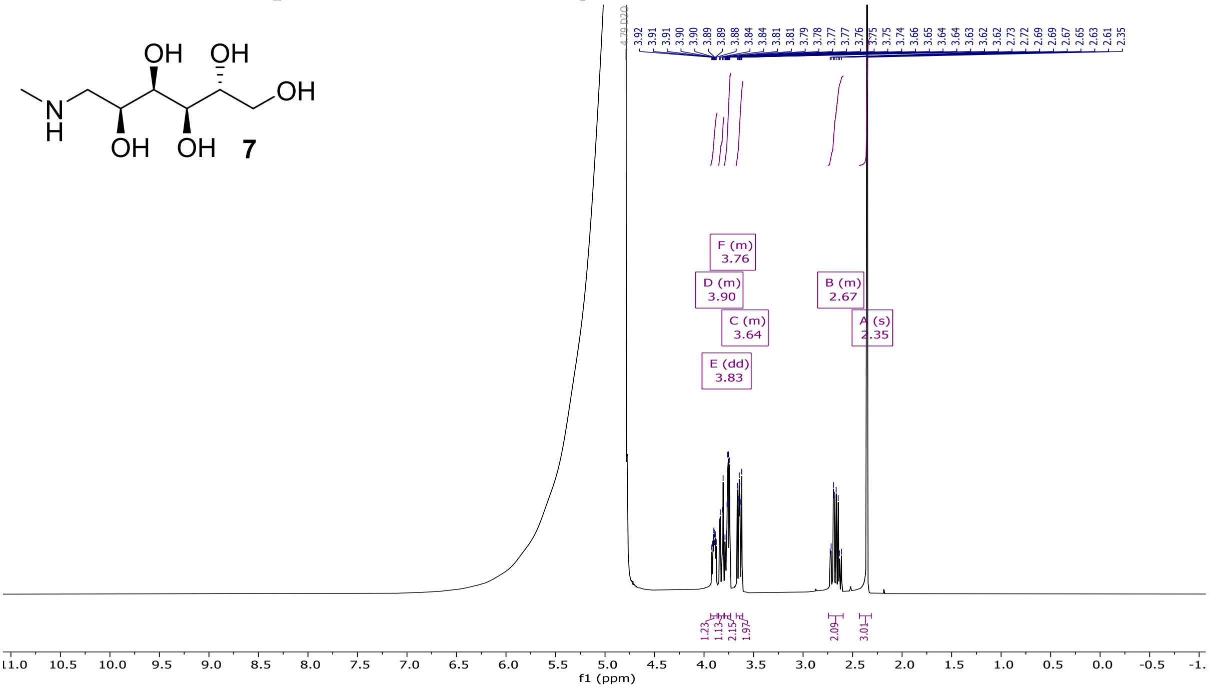

Figure S35: ¹H-NMR spectrum of *N*-methylglucamine standard recorded in water suppression mode in D_2O.

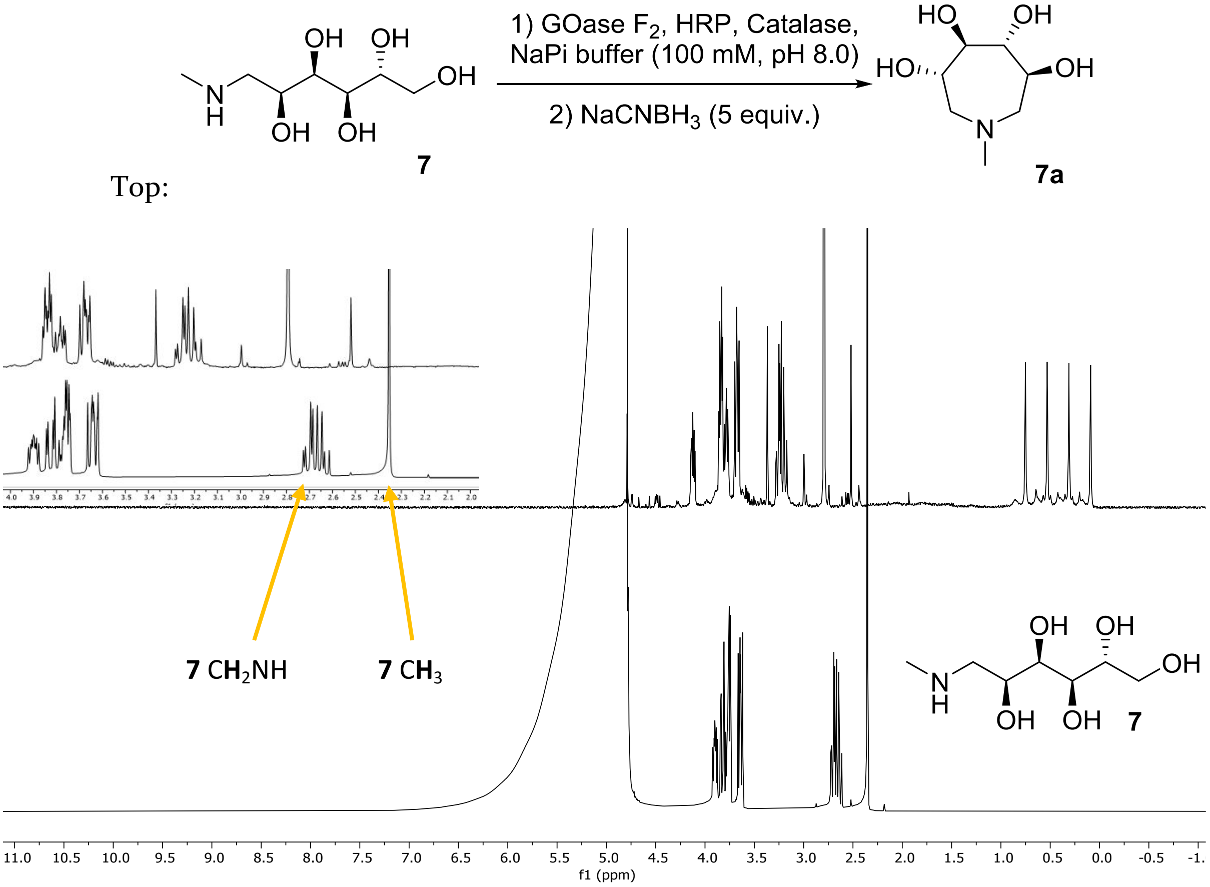

Figure S36: Comparison of crude ¹H-NMR for reaction of *N*-methylglucamine with GOase F_2-$NaCNBH_3$ (top) with *N*-methylglucamine standard (bottom). Expansion pane in top left shows

disappearance of key substrate peaks at 2.35 and 2.65 ppm and appearance of new product peaks.

Characterisation of biocatalytically synthesised products

3,4-dihydroxypiperidine **1a**

A 10 mL biocatalytic cascade reaction with substrate **1** (10 mM), immobilised GOase F_2 (100 mg, 10 w% enzyme loading), HRP (0.1 mg mL^{-1}) and catalase (0.1 mg mL^{-1}) in NaPi buffer (100 mM, pH 8.0) was incubated at 25 °C for 6 hours. The reaction was then supplemented with purified pRed-14 (0.5 mg mL^{-1} final), NAD^+ (0.5 mM), PtDH lysate (0.2 mg mL^{-1}) and NaPt (50 mM final from a 1M stock at pH 8.0) and incubated at 25 °C for a further 16 hours. $CaCl_2$ was added to precipitate phosphate salts and the suspension was filtered (30K MWCO) and lyophilised. The residue was resuspended in minimal water and loaded on to a column of Dowex 50WX8 NH_4^+ form resin. The loaded column was washed with H_2O (3–5 cv.) and then fractions were collected with an increasing concentration of aqueous NH_3 (0–1 M, 50 mM increments, 2 cv. per solution). The fractions were analysed by TLC. Fractions containing product were pooled and concentrated *in vacuo* to afford the title compound as a colourless oil (7.5 mg, 64%). δH (400 MHz, D_2O) 3.95 – 3.89 (m, 2H), 3.12–3.04 (m, 2H), 2.95–2.89 (m, 1H), 2.83–2.76 (m, 1H), 1.91–1.76 (m, 2H); δC (101 MHz, D_2O) 67.5, 66.7, 46.9, 41.2, 27.3; data consistent with the commercial standard. HRMS (ESI) *m/z* calculated for $C_5H_{11}NO_2$ $[M+H]^+$ 118.0790, found 118.0868 $[M+H]^+$

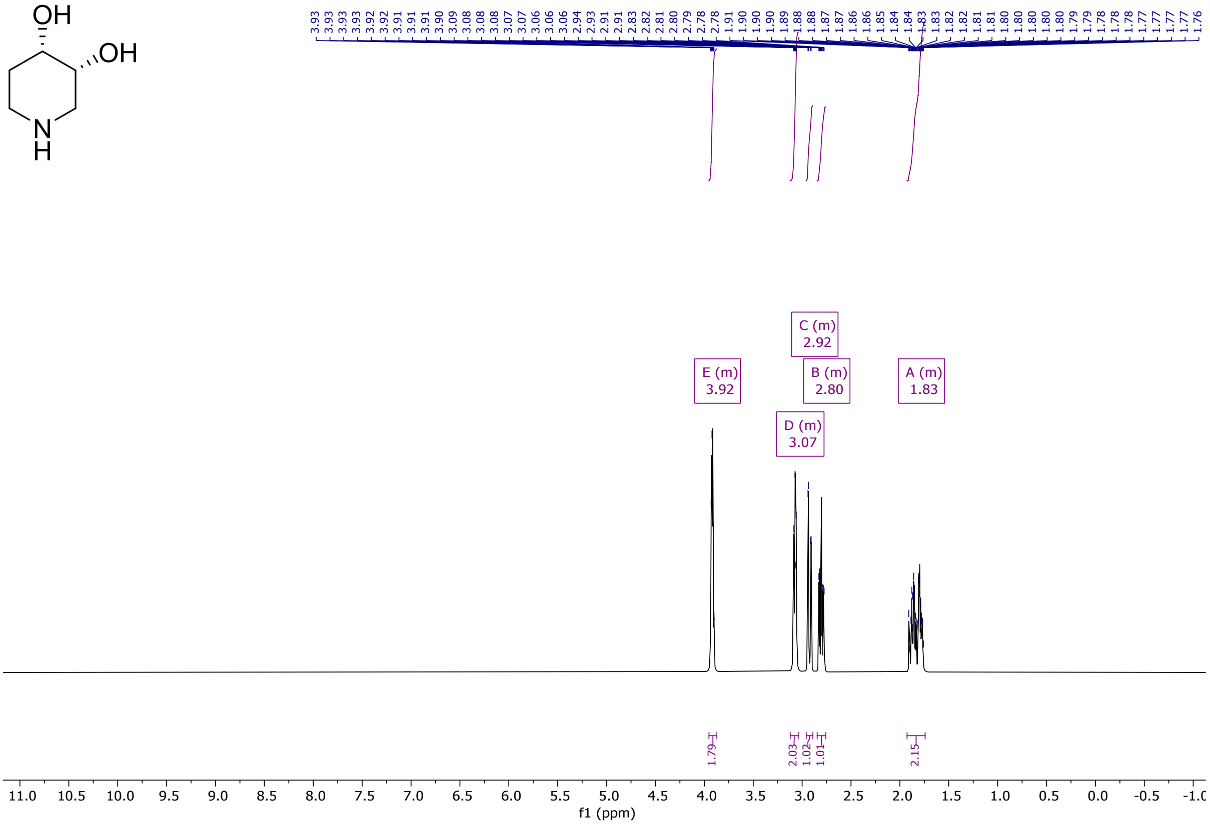

Figure S37: ^{1}H-NMR spectra of (3*R*, 4*S*)-3,4-dihydroxypiperidine **1a** synthesised through the GOase-SDH cascade recorded on a 500 MHz Bruker spectrometer.

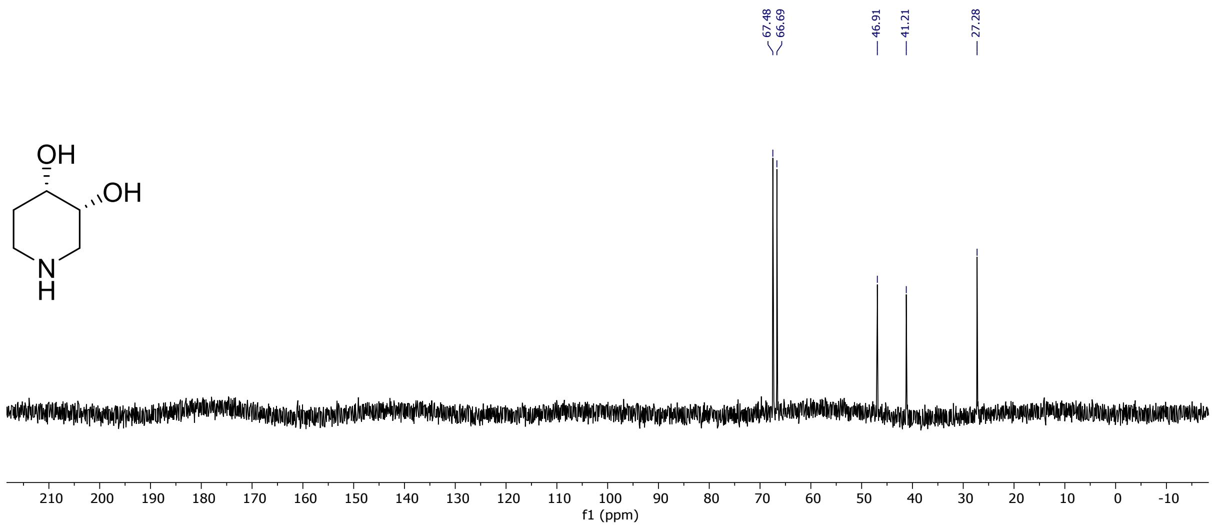

Figure S38: ^{13}C-NMR spectra of (3*R*, 4*S*)-3,4-dihydroxypiperidine **1a** synthesised through the GOase-SDH cascade recorded on a 500 MHz Bruker spectrometer.

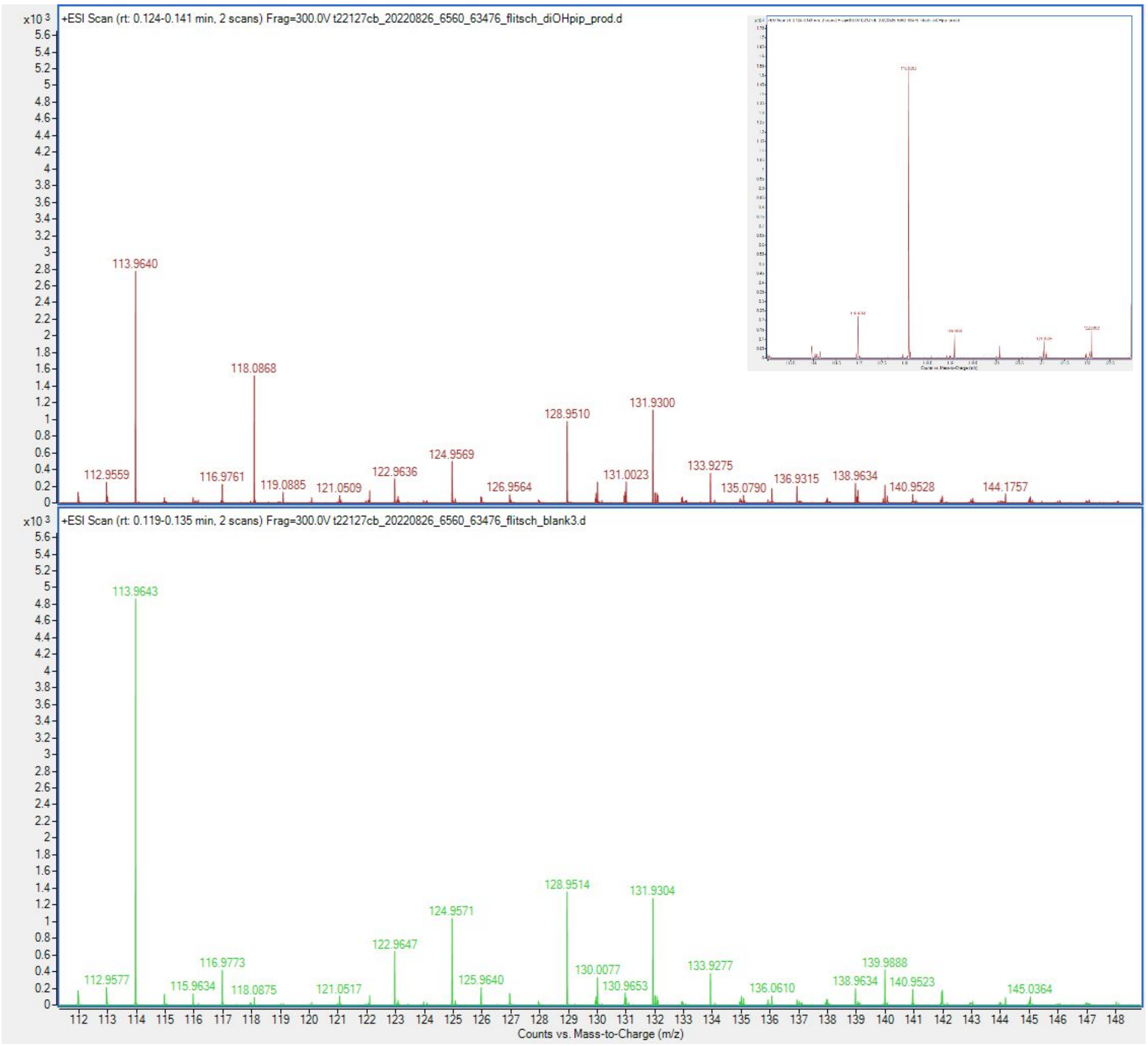

Figure S39: HRMS (ESI) of 3,4-dihydroxypiperidine **1a** synthesised through the GOase-SDH cascade. Background subtracted spectra in the top right expansion pane.

N-methyl-3,4,5,6-tetrahydroxyazepane **7a**

A 10 mL biocatalytic cascade reaction with substrate **7** (10 mM), immobilised GOase F_2 (100 mg, 10 w% enzyme loading), HRP (0.1 mg mL^{-1}) and catalase (0.1 mg mL^{-1}) in NaPi buffer (100 mM, pH 8.0) was incubated at 25 °C for 6 hours. The reaction was then supplemented with $NaCNBH_3$ (50 mM) and incubated at 25 °C for a further 16 hours. $CaCl_2$ was added to precipitate phosphate salts and the suspension was filtered (30K MWCO) and lyophilised. The residue was resuspended in minimal water and loaded on to a column of Dowex 50WX8 H^+ form resin (prewashed with water). The loaded column was washed once more with H_2O (3–5 cv.) and then fractions were collected with an increasing concentration of aqueous NH_3 (0–1 M, 50 mM increments, 3 cv. per solution). Fractions containing product (as determined by UPLC-QDa) were pooled and concentrated *in vacuo* to afford the title compound **7a** as a yellow oil (13 mg, 73%). δH (400 MHz, D_2O) 4.11 (ddd, *J* = 9.0, 5.1, 3.6 Hz, 1H), 3.88–3.80 (m, 2H), 3.80–3.73 (m, 1H), 3.71–3.61 (m, 2H), 3.29–3.14 (m, 2H), 2.78 (NC**H**$_3$, s, 3H); δC (101 MHz, D_2O) 70.9, 70.7, 70.6, 68.1, 62.7, 51.1, 33.0. HRMS (ESI) *m/z* calculated for $C_7H_{15}NO_4$ $[M+H]^+$ 178.1001, found 178.1082 $[M+H]^+$ and 196.1190 $[M+NH_4]^+$.

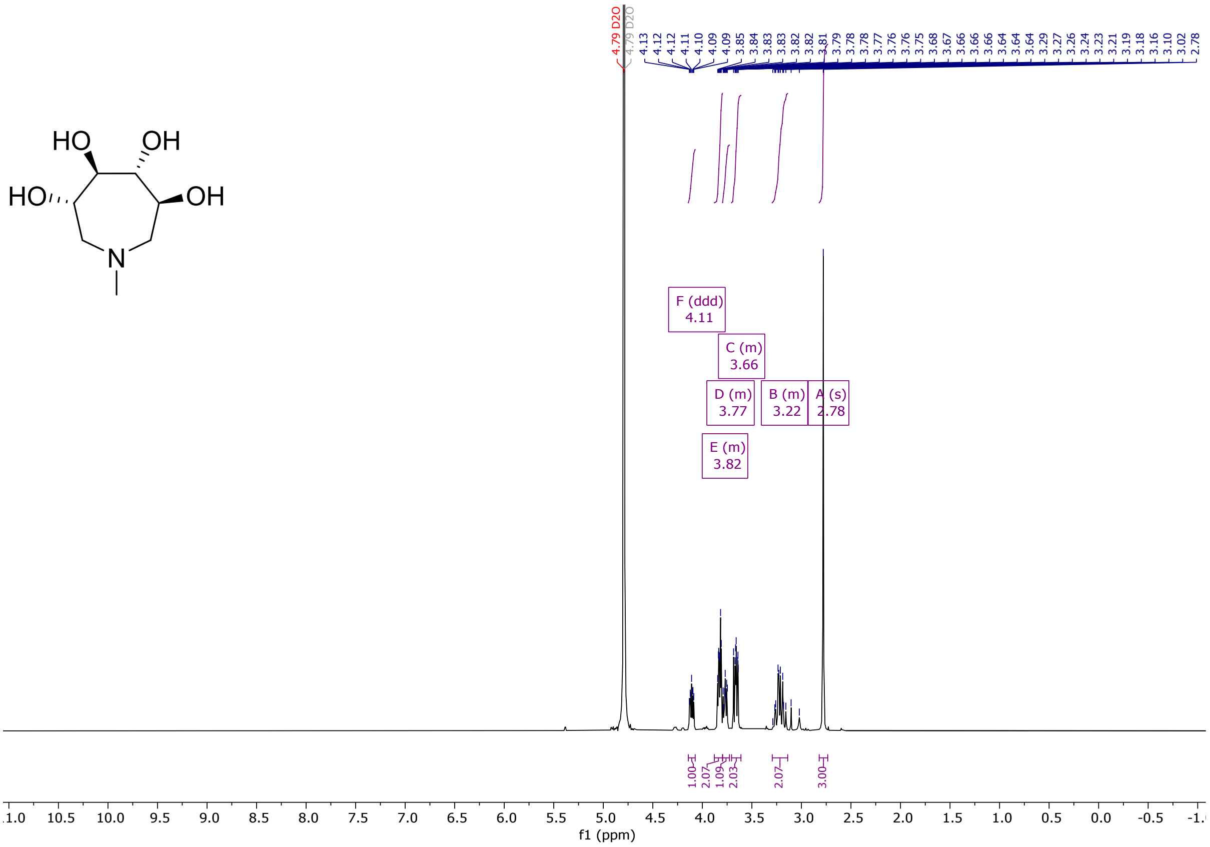

Figure S40: ^{1}H-NMR spectra of *N*-methyl-3,4,5,6-tetrahydroxyazepane **7a** synthesised through the GOase-$NaCNBH_3$ cascade.

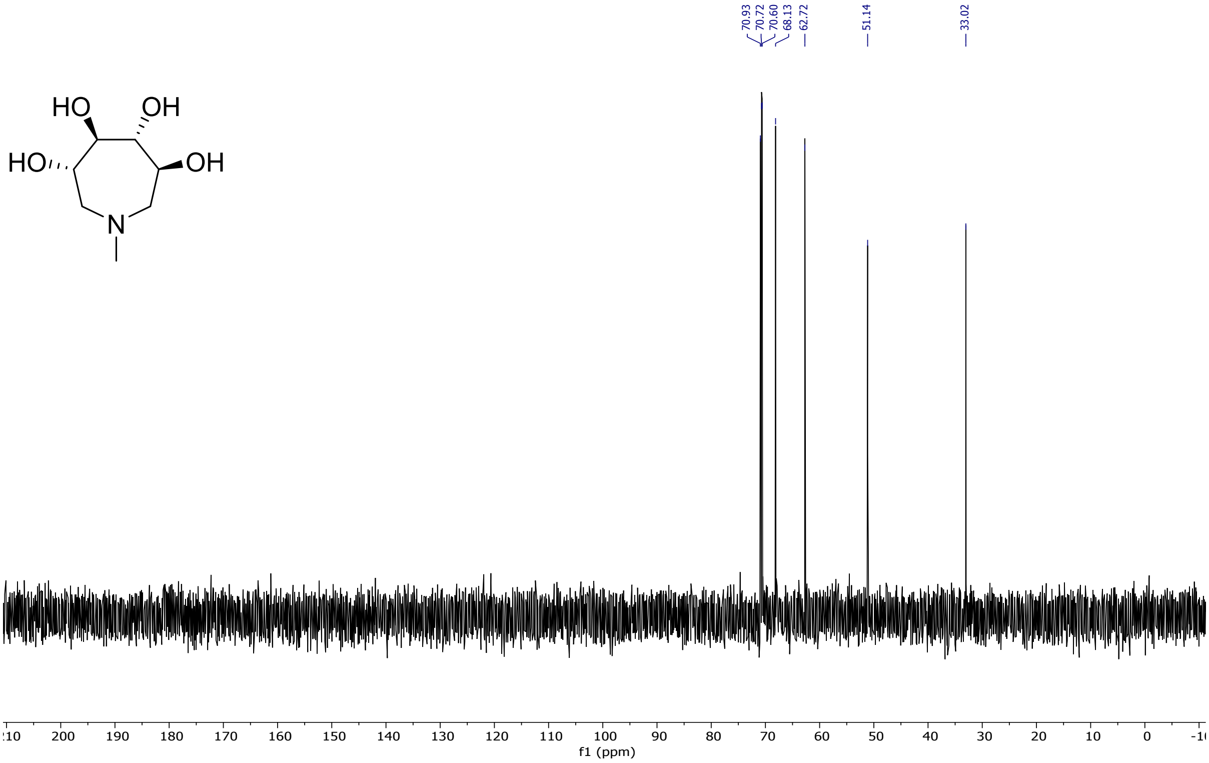

Figure S41: ^{13}C-NMR spectra of *N*-methyl-3,4,5,6-tetrahydroxyazepane **7a** synthesised through the GOase- $NaCNBH_3$ cascade.

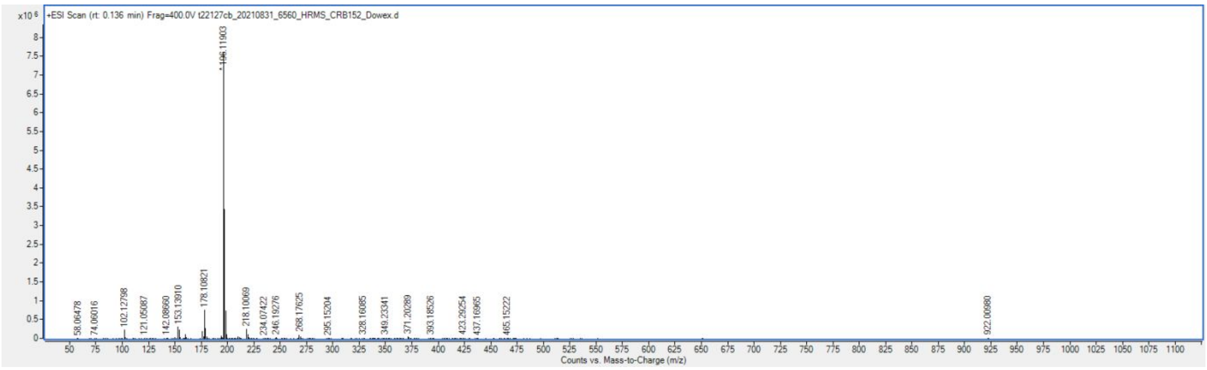

Figure S42: HRMS (ESI) of *N*-methyl-3,4,5,6-tetrahydroxyazepane **7a** synthesised through the GOase- $NaCNBH_3$ cascade.

Chemical synthesis

1-amino-2-deoxy-D-ribose.HCl (1)

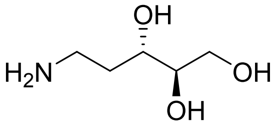

To a solution of 2-deoxyribose (536 mg, 4 mmol) in a saturated solution of NH_4OAc in EtOH (80 mL) were added $NaCNBH_3$ (744 mg, 12 mmol) and 30% aq. NH_3 (32 mL). The mixture was stirred at reflux for 18 h, cooled to room temperature and concentrated under reduced pressure. The residue was redissolved in water (10 mL, loaded on to a column of Dowex ion-exchange resin (prewashed with water), and washed with water (100 mL) to remove excess salt. The amine product was then eluted with 30% aq NH_3 (30 mL). The eluent was concentrated under reduced pressure, resuspended in HCl (1 mL, 1M) and concentrated again to afford the title compound as an off-white oil (399 mg, 73%). δH (400 MHz, D_2O) 3.80–3.70 (m, 2H), 3.66–3.56 (m, 2H), 3.31–3.08 (m, 2H), 2.10–1.96 (m, 1H), 1.90–1.74 (m, 1H); δC (101 MHz, D_2O) 74.3, 69.6, 62.3, 37.2, 29.3; data consistent with those reported in the literature. [9,10]

1-amino-D-xylitol.HCl (2)

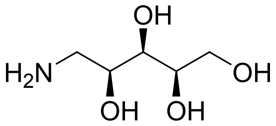

To a solution of D-xylose (150 mg, 1 mmol) in a saturated solution of NH_4OAc in EtOH (20 mL) were added $NaCNBH_3$ (188 mg, 3 mmol) and 30% aq. NH_3 (8 mL). The mixture was stirred at reflux for 18 h, cooled to room temperature and concentrated under reduced pressure. The residue was redissolved in water (5 mL, loaded on to Dowex ion-exchange resin (pre-washed with water), and washed with water (100 mL) to remove excess salt. The amine product was then eluted with 30% aq. NH_3 (30 mL). The eluent was concentrated under reduced pressure, resuspended in HCl (1 mL, 1M) and concentrated again to afford the title compound as an off-white solid (78 mg, 51%). δH (400 MHz, D_2O) 3.86 – 3.77 (m, 1H), 3.64 – 3.57 (m, 1H), 3.52 (dd, J = 11.8, 4.5 Hz, 1H), 3.47–3.40 (m, 3H), 3.13–2.89 (m, 2H); δC (101 MHz, D_2O) 71.52, 71.50, 67.7, 62.2, 41.9; data consistent with those reported in the literature.[9,10]

1-amino-D-arabinitol.HCl (3)

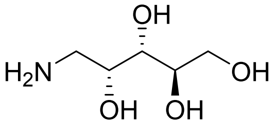

To a solution of D-arabinose (150 mg, 1 mmol) in a saturated solution of NH_4OAc in EtOH (20 mL) were added $NaCNBH_3$ (188 mg, 3 mmol) and 30% aq. NH_3 (8 mL). The mixture was stirred at reflux for 18 h, cooled to room temperature and concentrated under reduced pressure. The residue was redissolved in water (5 mL, loaded on to Dowex ion-exchange resin (pre-washed with water), and washed with water (100 mL) to remove excess salt. The amine product was then eluted with 30% aq. NH_3 (30 mL). The eluent was concentrated under reduced pressure, resuspended in HCl (1 mL, 1M) and concentrated again to afford the title compound as an off-white solid (45 mg, 29%). δH (400 MHz, D_2O) 4.24–4.11 (m, 1H), 3.89–3.80 (m, 1H), 3.79–3.72 (m, 1H), 3.72–3.64 (m, 1H), 3.57–3.50 (m, 1H), 3.25–3.12 (m, 2H); δC (101 MHz, D_2O) 71.2, 70.5, 66.3, 62.7, 42.5; data consistent with those reported in the literature.[9,10]

Glucosaminitol.HCl (4)

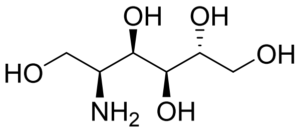

To a stirred solution of glucosamine.HCl (1.0 g, 4.64 mmol) in water (20 mL) was added $NaBH_4$ (263 mg, 6.96 mmol, 1.5 eq.) in water (10 mL). The solution was stirred at room temperature for 18 h, quenched with conc. HCl and concentrated under reduced pressure. The resulting yellow oil was redissolved in water (10 mL), loaded onto a column of Dowex ion-exchange resin (50WX8, prewashed with water) and washed with water (100 mL). The amine product was then eluted with 30% aq. NH_3 (50 mL) and concentrated under reduced pressure to afford the title compound as a yellow solid (900 mg, 89%). δH (400 MHz, D_2O) 4.13–4.08 (m, 1H), 3.93–3.82 (m, 2H), 3.81–3.73 (m, 2H), 3.72–3.63 (m, 2H), 3.58–3.51 (m, 1H); δC (101 MHz, D_2O) 70.8 (**C**H), 70.4 (**C**H), 66.0 (**C**H), 62.7 (**C**H_2), 58.8 (**C**H_2), 55.4 (**C**H); data consistent with those reported in the literature.[11]

Galactosaminitol.HCl (5)

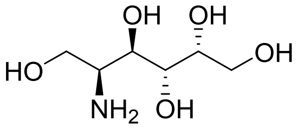

To a stirred solution of galactosamine.HCl (1.0 g, 4.64 mmol) in water (20 mL) was added $NaBH_4$ (263 mg, 6.96 mmol, 1.5 eq.) in water (10 mL). The solution was stirred at room temperature for 18 h, quenched with conc. HCl and concentrated under reduced pressure. The resulting yellow oil was redissolved in water (10 mL), loaded onto a column of Dowex ion-exchange resin (prewashed with water) and washed with water (100 mL). The amine product was then eluted with 30% aq. NH_3 (50 mL), concentrated under reduced pressure, resuspended in HCl (1 mL, 1M) and concentrated again to afford the title compound as a yellow syrup (590 mg, 58%). δH (400 MHz, D_2O) 3.93–3.86 (m, 3H), 3.79 (dd, J = 11.9, 8.4 Hz, 1H), 3.72–3.68 (m, 2H), 3.68–3.64 (m, 2H); δC (101 MHz, D_2O) 70.8 (**C**H), 70.2 (**C**H), 66.7 (**C**H), 62.8 (**C**H_2), 59.9 (**C**H_2), 53.9 (**C**H); data consistent with those reported in the literature.[11]

NMR spectra for chemically synthesised compounds

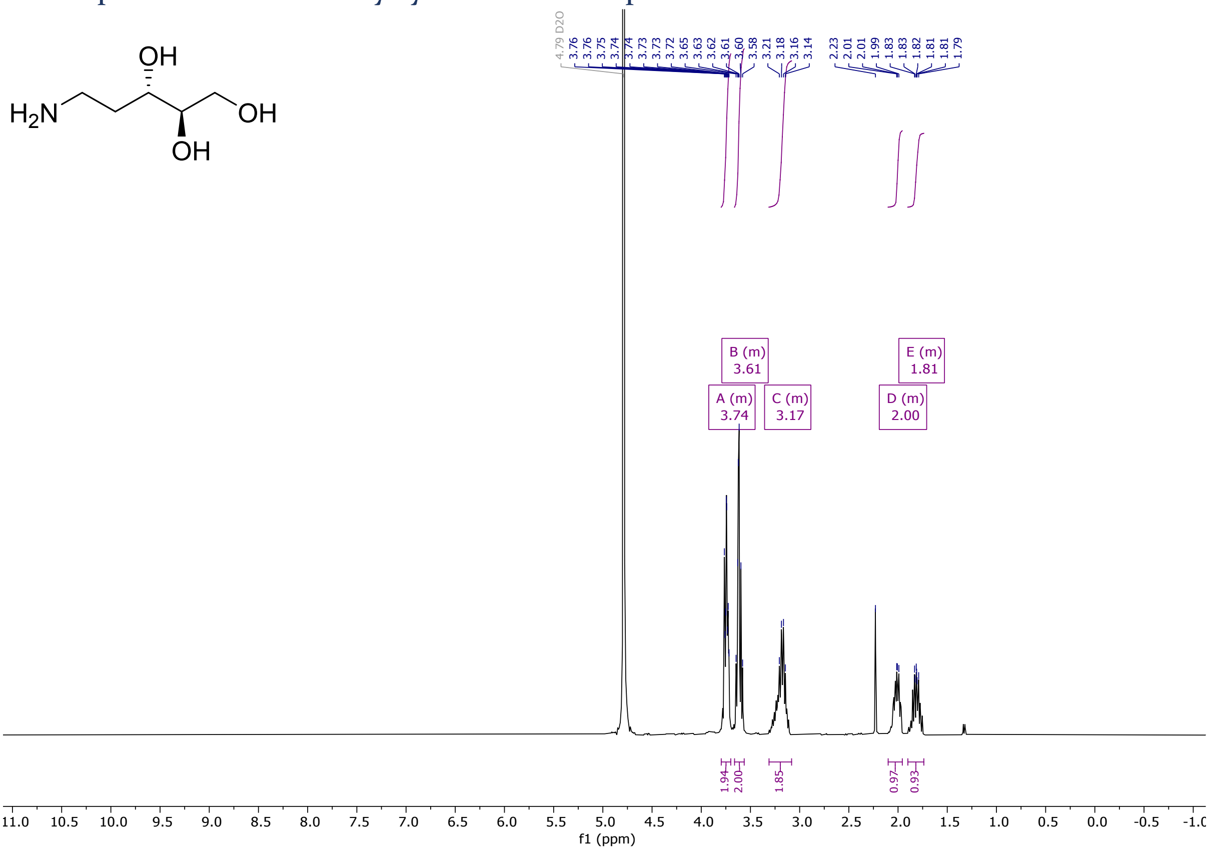

Figure S43: ^{1}H-NMR of chemically synthesised 1-amino-2-deoxy-D-ribose.HCl (**1**)

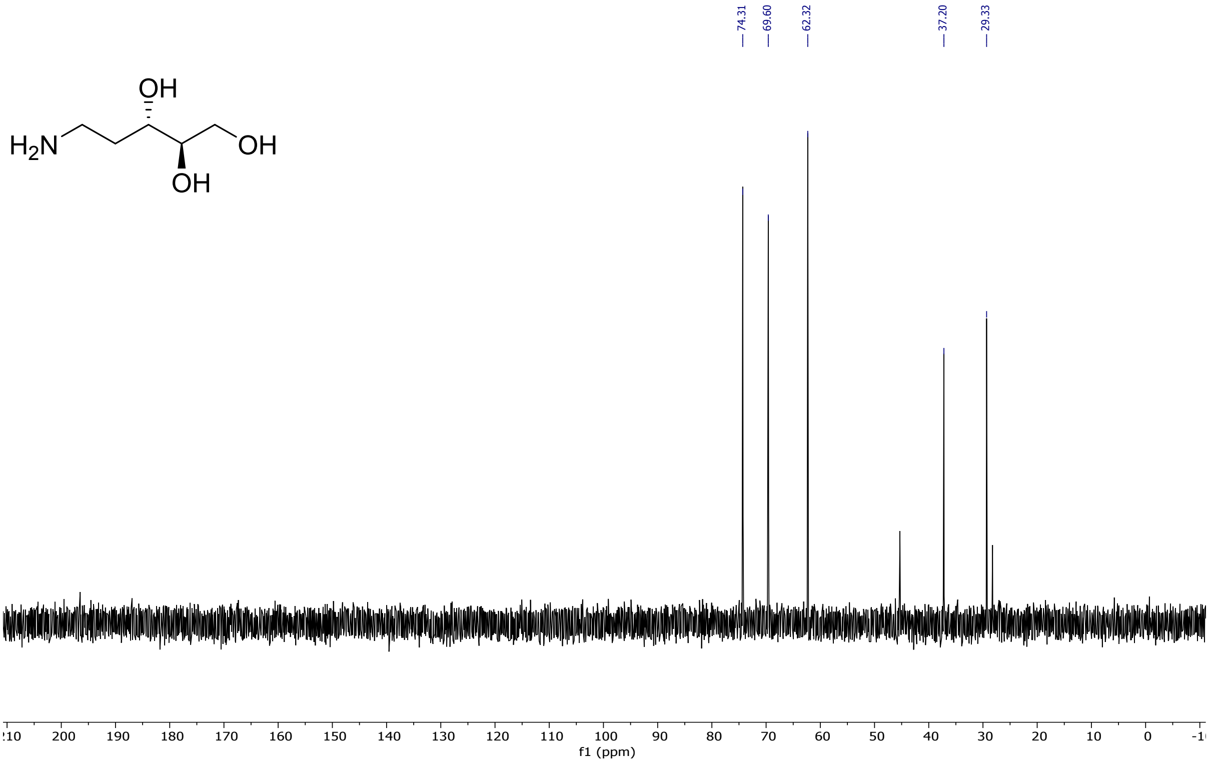

Figure S44: ^{13}C-NMR of chemically synthesised 1-amino-2-deoxy-D-ribose.HCl (**1**)

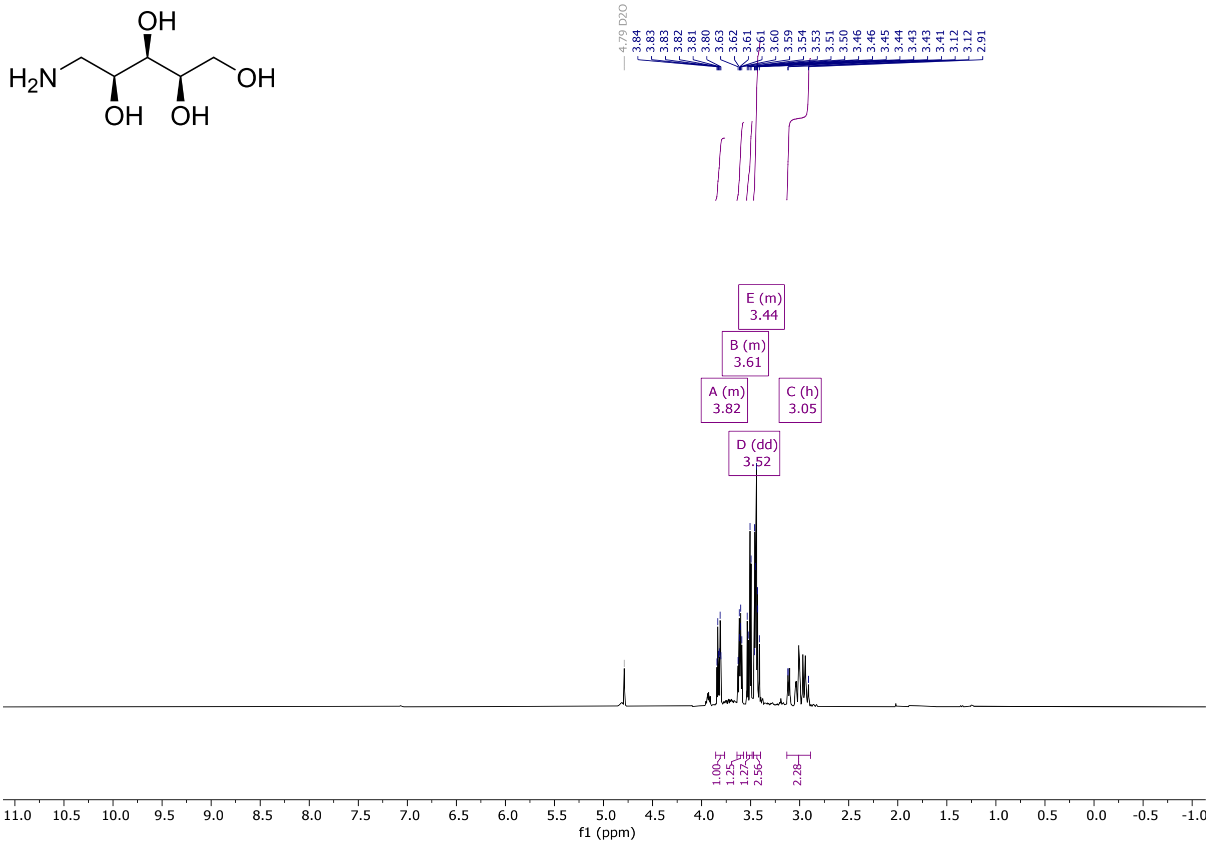

Figure S45: ^{1}H-NMR of chemically synthesised 1-amino-D-xylitol.HCl (**2**)

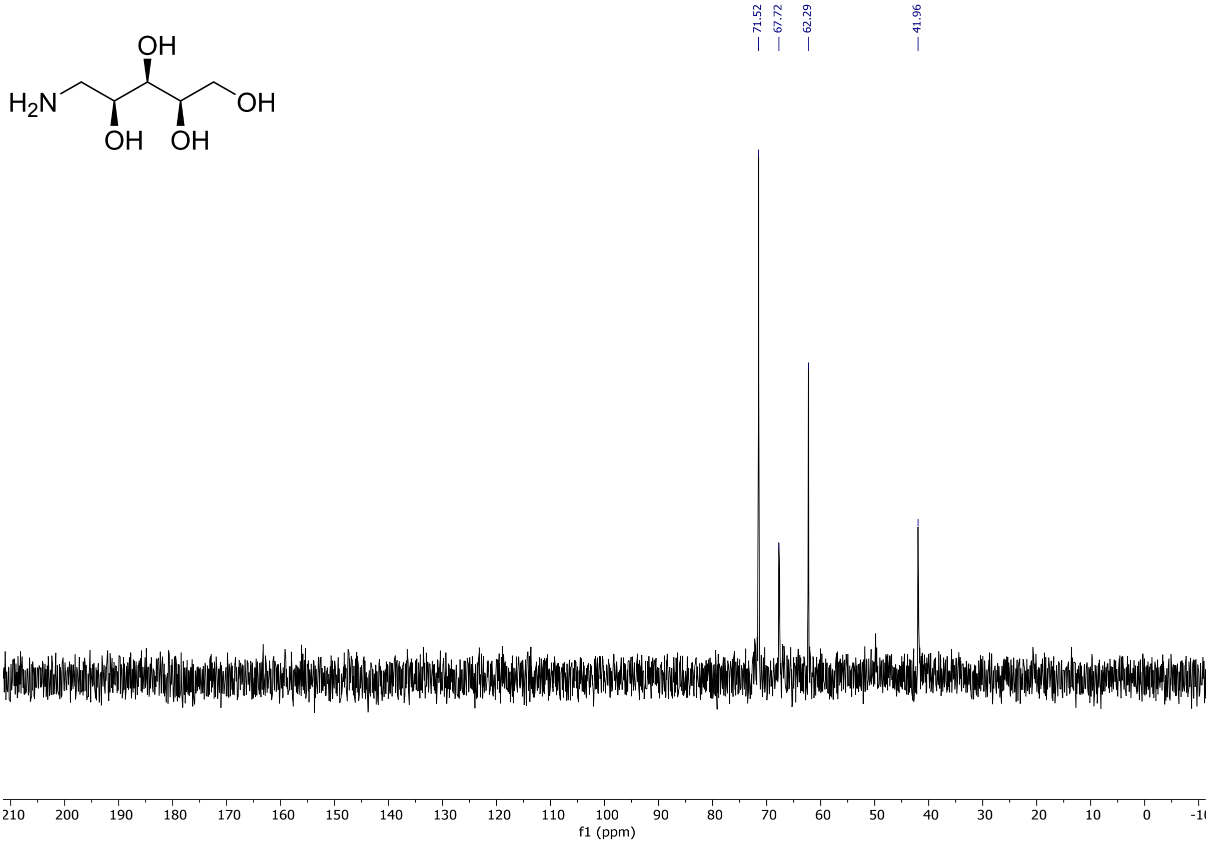

Figure S46: ^{13}C-NMR of chemically synthesised 1-amino-D-xylitol.HCl (**2**)

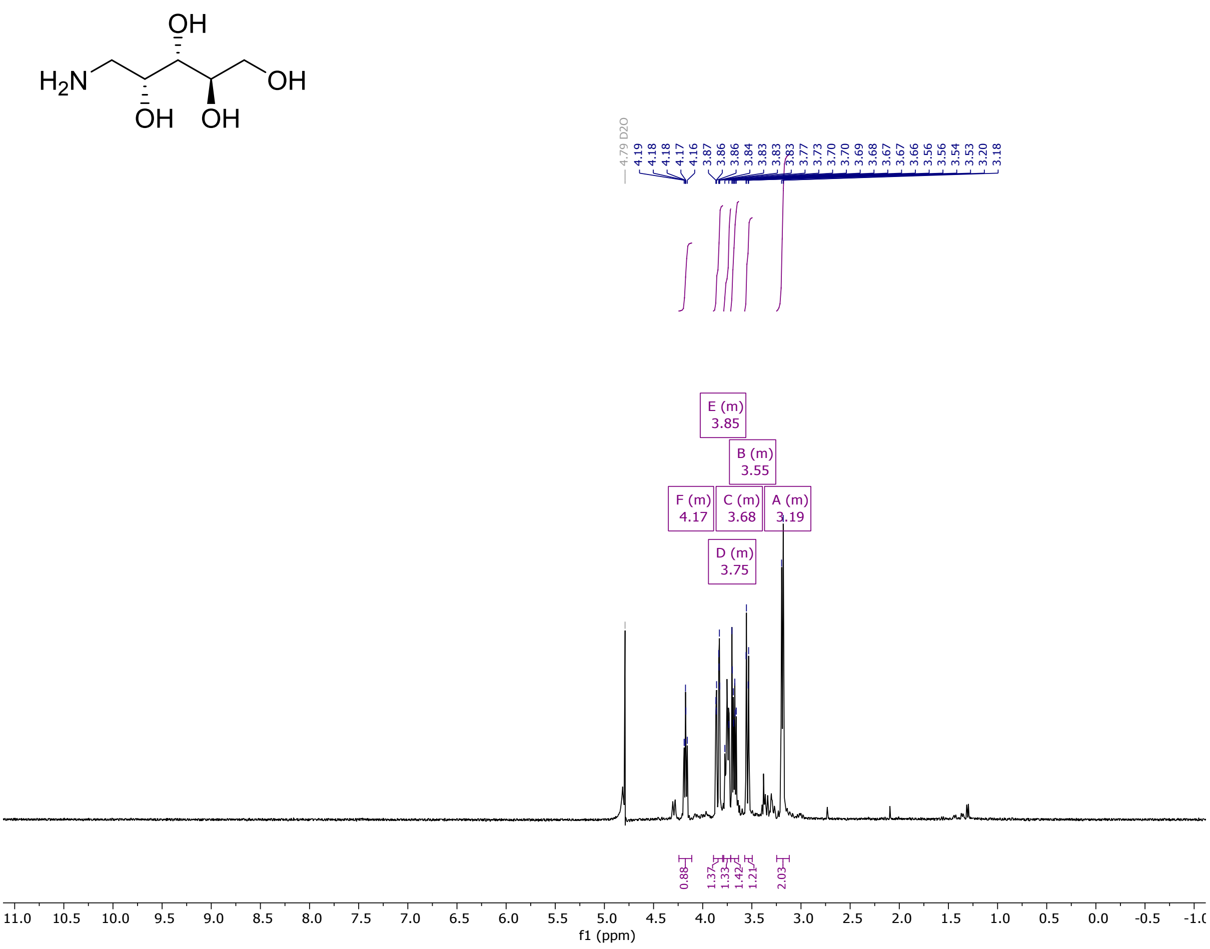

Figure S47: ^{1}H-NMR of chemically synthesised 1-amino-D-arabinitol.HCl (**3**)

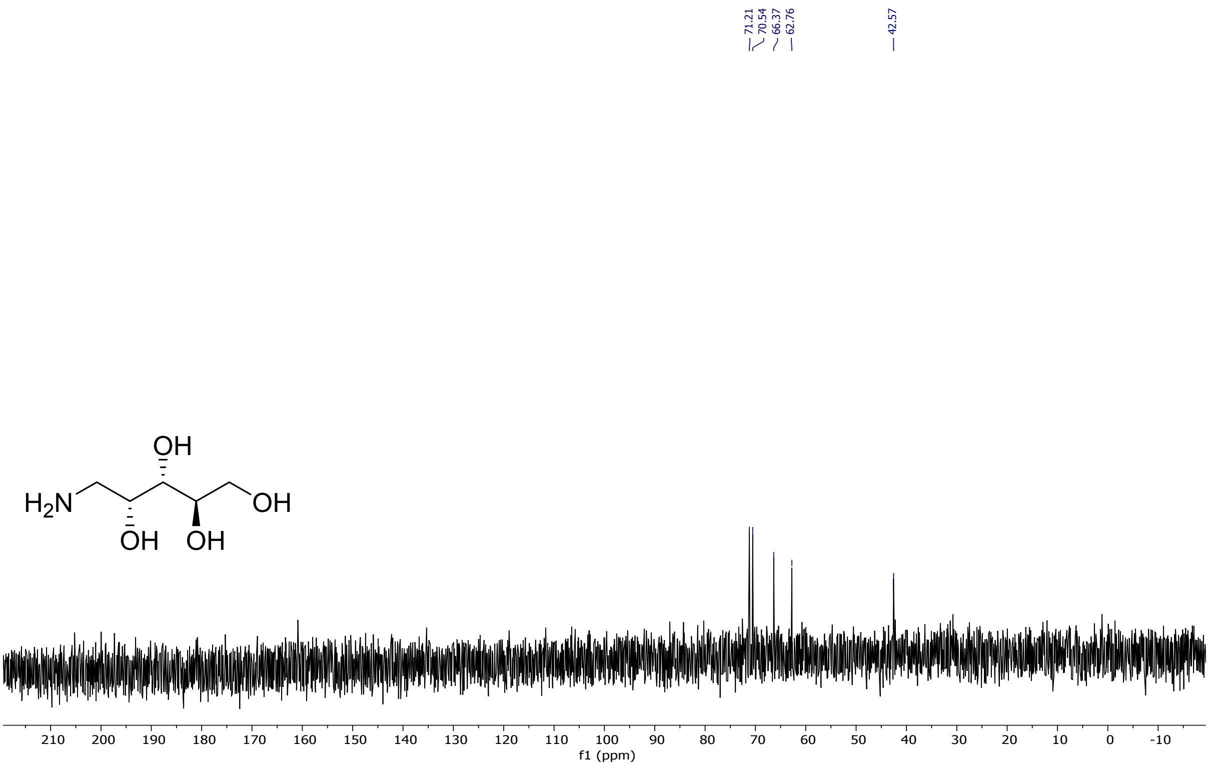

Figure S48: ^{13}C-NMR of chemically synthesised 1-amino-D-arabinitol.HCl (**3**)

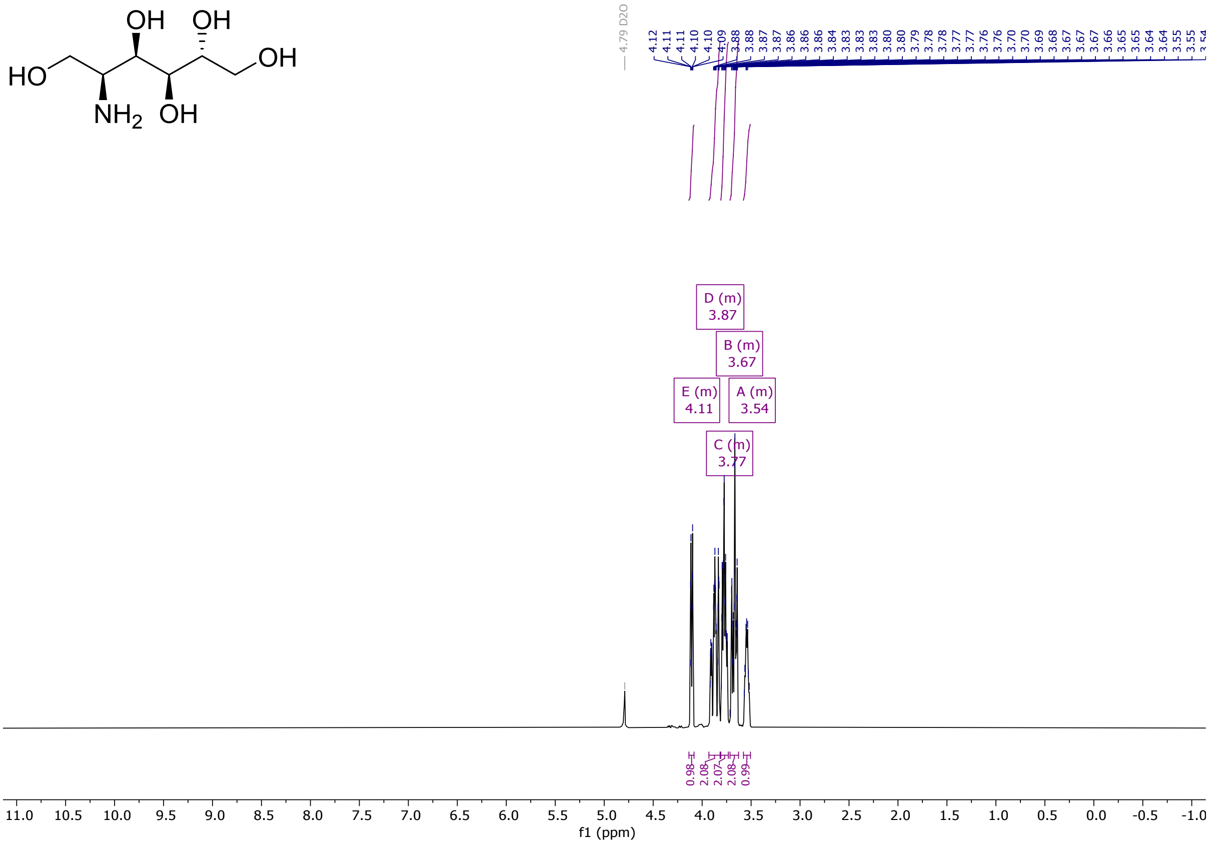

Figure S49: ^{1}H-NMR of chemically synthesised glucosaminitol.HCl (**4**)

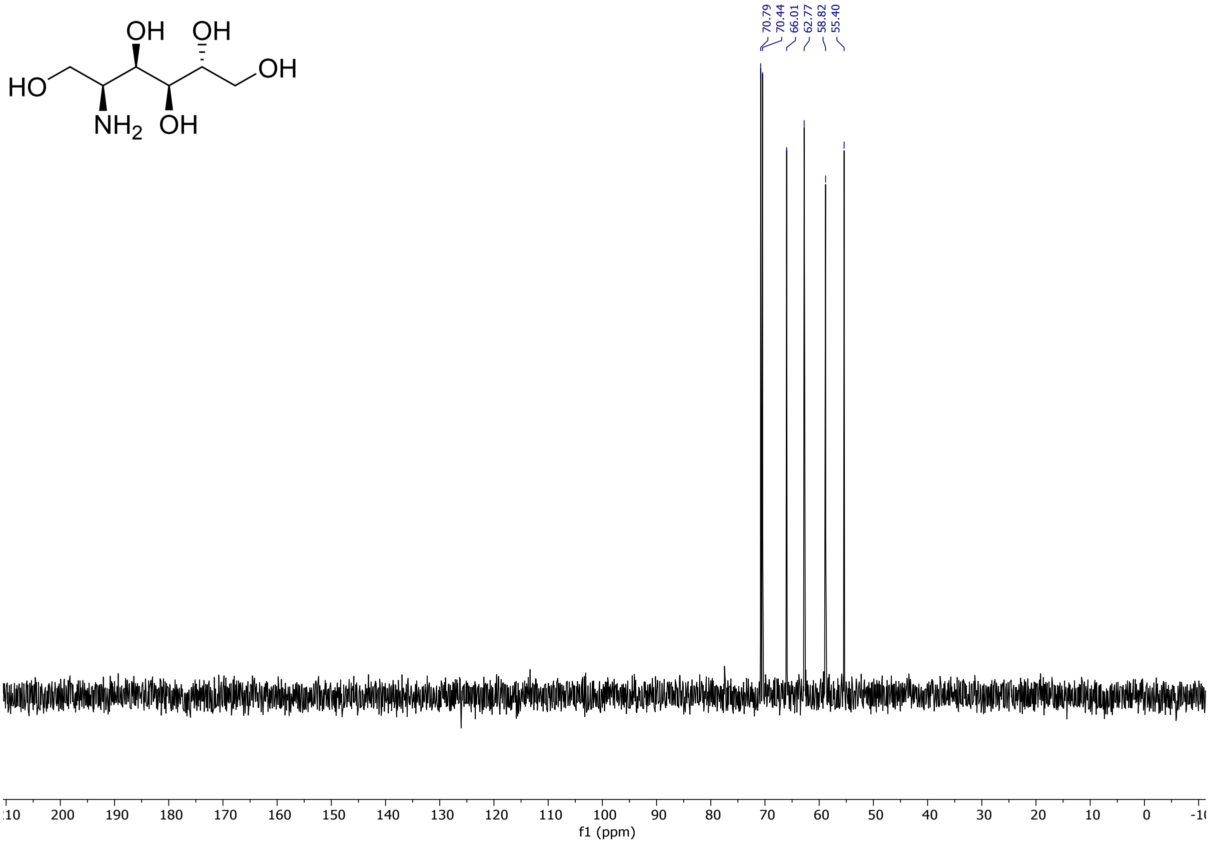

Figure S50: ^{13}C-NMR of chemically synthesised glucosaminitol.HCl (**4**)

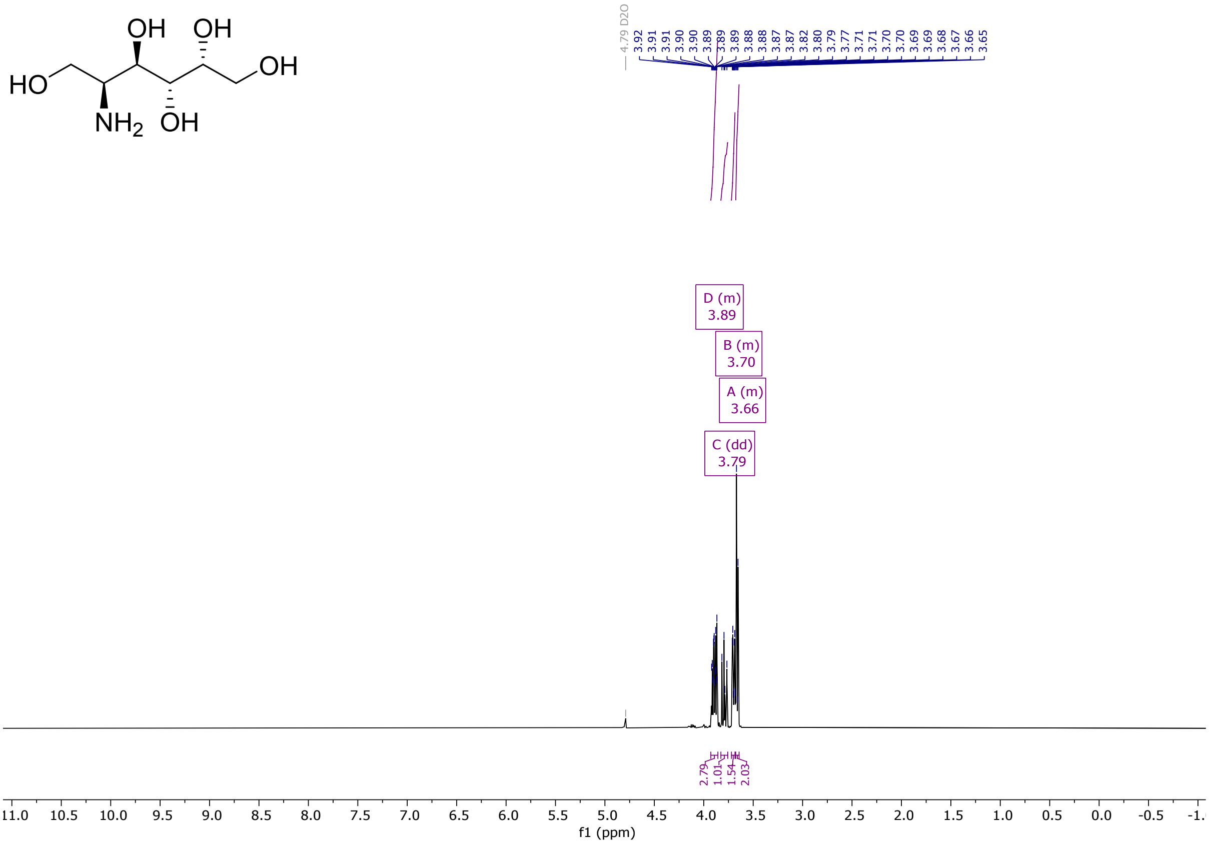

Figure S51: ^{1}H-NMR of chemically synthesised galactosaminitol.HCl (**5**)

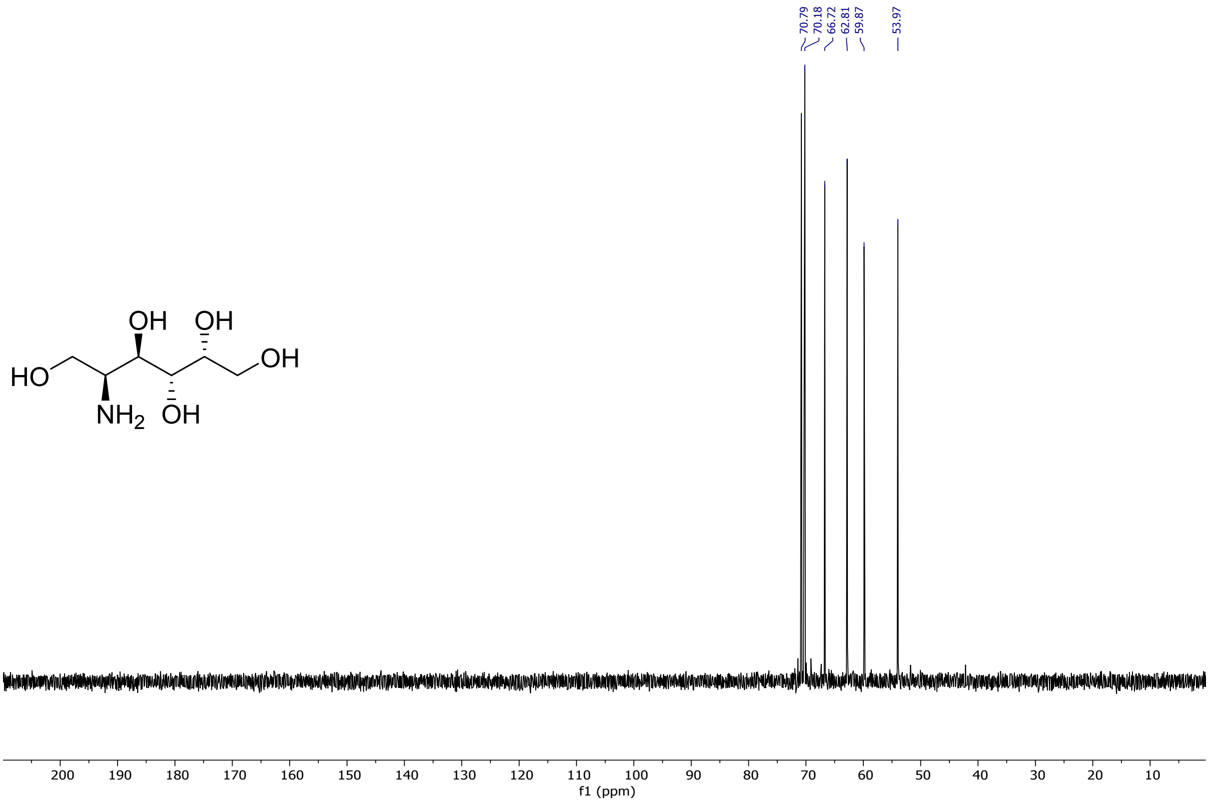

Figure S52: ^{13}C-NMR of chemically synthesised galactosaminitol.HCl (**5**)

References

(1) Mattey, A. P.; Sangster, J. J.; Ramsden, J. I.; Baldwin, C.; Birmingham, W. R.; Heath, R. S.; Angelastro, A.; Turner, N. J.; Cosgrove, S. C.; Flitsch, S. L. Natural Heterogeneous Catalysis with Immobilised Oxidase Biocatalysts. *RSC Adv.* **2020**, *10* (33), 19501–19505. https://doi.org/10.1039/D0RA03618H.

(2) Deacon, S. E.; McPherson, M. J. Enhanced Expression and Purification of Fungal Galactose Oxidase in Escherichia Coli and Use for Analysis of a Saturation Mutagenesis Library. *ChemBioChem* **2011**, *12* (4), 593–601. https://doi.org/10.1002/cbic.201000634.

(3) Medema, M. H.; Takano, E.; Breitling, R. Detecting Sequence Homology at the Gene Cluster Level with MultiGeneBlast. *Mol Biol Evol* **2013**, *30* (5), 1218–1223. https://doi.org/10.1093/molbev/mst025.

(4) Clark, L. F.; Johnson, J. V.; Horenstein, N. A. Identification of a Gene Cluster That Initiates Azasugar Biosynthesis in Bacillus Amyloliquefaciens. *Chembiochem* **2011**, *12* (14), 2147–2150. https://doi.org/10.1002/cbic.201100347.

(5) Nuñez, C.; Horenstein, N. A. Functional Analysis of a Gene Cluster from Chitinophaga Pinensis Involved in Biosynthesis of the Pyrrolidine Azasugar DAB-1. *J Nat Prod* **2019**, *82* (12), 3401–3409. https://doi.org/10.1021/acs.jnatprod.9b00758.

(6) Marshall, J. R.; Yao, P.; Montgomery, S. L.; Finnigan, J. D.; Thorpe, T. W.; Palmer, R. B.; Mangas-Sanchez, J.; Duncan, R. A. M.; Heath, R. S.; Graham, K. M.; Cook, D. J.; Charnock, S. J.; Turner, N. J. Screening and Characterization of a Diverse Panel of Metagenomic Imine Reductases for Biocatalytic Reductive Amination. *Nat. Chem.* **2021**, *13* (2), 140–148. https://doi.org/10.1038/s41557-020-00606-w.

(7) *Deciphering Piperidine Formation in Polyketide-Derived Indolizidines Reveals a Thioester Reduction, Transamination, and Unusual Imine Reduction Process | ACS Chemical Biology*. https://pubs.acs.org/doi/abs/10.1021/acschembio.6b00875 (accessed 2022-11-15).

(8) Toftgaard Pedersen, A.; Birmingham, W. R.; Rehn, G.; Charnock, S. J.; Turner, N. J.; Woodley, J. M. Process Requirements of Galactose Oxidase Catalyzed Oxidation of Alcohols. *Org. Process Res. Dev.* **2015**, *19* (11), 1580–1589. https://doi.org/10.1021/acs.oprd.5b00278.

(9) Dangerfield, E. M.; Plunkett, C. H.; Win-Mason, A. L.; Stocker, B. L.; Timmer, M. S. M. Protecting-Group-Free Synthesis of Amines: Synthesis of Primary Amines from Aldehydes via Reductive Amination. *J. Org. Chem.* **2010**, *75* (16), 5470–5477. https://doi.org/10.1021/jo100004c.

(10) Braganza, C. D.; Shibata, K.; Fujiwara, A.; Motozono, C.; Sonoda, K.-H.; Yamasaki, S.; Stocker, B. L.; Timmer, M. S. M. The Effect of MR1 Ligand Glyco-Analogues on Mucosal-Associated Invariant T (MAIT) Cell Activation. *Org. Biomol. Chem.* **2019**, *17* (40), 8992–9000. https://doi.org/10.1039/C9OB01436E.

(11) Subrizi, F.; Benhamou, L.; Ward, J. M.; Sheppard, T. D.; Hailes, H. C. Aminopolyols from Carbohydrates: Amination of Sugars and Sugar-Derived Tetrahydrofurans with Transaminases. *Angewandte Chemie International Edition* **2019**, *58* (12), 3854–3858. https://doi.org/10.1002/anie.201813712.
