# Supplementary material for: Biocatalytic Cascades toward Iminosugar Scaffolds Reveal Promiscuous Activity of Shikimate Dehydrogenases
Source: ACS Cent Sci. 2023 Jan 11;9(1):103–8. doi: 10.1021/acscentsci.2c01169 (PMC9881201; doi:10.1021/acscentsci.2c01169)
Supplement: Supplementary file 2 — oc2c01169_si_002.pdf [file oc2c01169_si_002.pdf]

oc-2022-011698.R1

Name: Peer Review Information for "Biocatalytic cascades towards iminosugar scaffolds reveal promiscuous activity of shikimate dehydrogenases"

First Round of Reviewer Comments

Reviewer: 1

Comments to the Author

Aminosugars are pharmaceutically interesting molecules. In the manuscript, the authors designed two-step catalytic cascades for producing aminosugars from sugar-derived aminopolyols using RetroBioCat. They made great effort to the catalyst identification for the individual reactions including aminopolyol oxidation and imine reduction. Galactose oxidase (GO) variant F2 was found to be suited for aminopolyol oxidation. For the reduction of imines, bacterial shikimate dehydrogenases (SDHs) possessing promiscuous iminosugar reductase activity were discovered by genome mining. Due the narrow substrate scope of SDHs, chemical reduction of other iminosugars was performed by using NaCNBH3. Except for 5a (32%), other target products were obtained with >80% conversions. The experiments were designed rationally and performed well. Also, the manuscript was written well. Therefore, the reviewer believes that this manuscript may merit its publication in ACS Central Chemistry. Besides, other comments were listed as follows.

1. Page 1, right column, line 34: why was the ref. 18 cited after “reduction (Figure 1. a)”?

2. In the manuscript, some results were presented in Supporting Information, but the ambiguous information was provided in the text. For example, page 3, left column, lines 45-48: in the sentence “Interestingly, the sequential addition…… than the telescopic reactions (Supporting Information)”. Please provide the defined information (which Table/Figure).

3. In Figure 2: The results were presented by substrate conversions. However, the cascade was composed of two reactions (aminopolyol oxidation and imine reduction). The substrates aminopolyols may be converted into both the intermediate iminosugars and the target aminosugars. In addition, the formation of byproduct was observed by the authors. So, the substrate conversion is not a good index for the cascades. Likely, the product yield may be better.

4. Figure S6: The title is incomplete.

Reviewer: 2

Comments to the Author

This is a very interesting manuscript by Flitch et al describing the biocatalytic synthesis of iminosugars from sugar-derived aminopolyols, without requiring the use of protecting groups. Importantly, bacterial shikimate dehydrogenases were identified and acted as iminosugar reductases. The two-step strategy was applied to produce several iminosugars. The approach is novel and the manuscript well written. Acceptance is recommended after the following minor revisions have been made.

- In table/figure captions the use of purified enzymes/immobilised enzymes or clarified lysates should be indicated and highlighted further in the text (and concentrations used).

- Why were enzyme lysates not used more widely (which are often preferred in industry).

- Protein SDS page gels should be added to the SI and levels of enzyme production.

- In the SI the Figure 6 caption is obscured.

- There are several Figures in the SI, with data very briefly being mentioned in the main text. These SI Figures should be mentioned in the text to help the reader understand where this data is.

Author's Response to Peer Review Comments:

Professor Sabine L Flitsch
School of Chemistry & MIB
131 Princess Street
Manchester M1 7DN
Tel: +44 (0) 161 306 5172/275 1312
sabine.flitsch@manchester.ac.uk

November 22, 2022

Journal: ACS Central Science
Manuscript ID: oc-2022-011698
Original Submission Date: 05-Oct-2022 – manuscript revision request
Title: "Biocatalytic cascades towards iminosugar scaffolds reveal promiscuous activity of shikimate dehydrogenases"
Author(s): Swanson, Christopher; Ford, Grayson; Matthey, Ashley; Gourbeyre, Lea; Flitsch, Sabine Dear

Professor Editor,

Thank you very much for your positive consideration of our manuscript. We are very grateful to yourself and the referees for your helpful comments and have been able to make all the requested changes requested. A list of detailed corrections is attached below.

We look forward to hearing from you in due course.

Yours sincerely,

Response to reviewer comments for 'Biocatalytic cascades towards iminosugar scaffolds reveal promiscuous activity of shikimate dehydrogenases.' Manuscript ID: oc-2022-011698

Formatting Needs:

- AU NAMES: Please correct this discrepancy in the author list: Ashley Mattey (MS/SI) vs Ashley Matthey (P+)
- AU EMAIL: Please include the email address of the corresponding author on the first page of the manuscript, and the Supporting Information if submitted, with an asterisk next to their name in the author list. Please be sure to label "email."
- SI PG#S: The supporting information pages must be numbered consecutively, starting with page S1.

These formatting changes have now been made, thank you for highlighting them.

Reviewer(s)' Comments to Author:

Reviewer: 1

1. Page 1, right column, line 34: why was the ref. 18 cited after "reduction (Figure 1. a)"?

We thank the reviewer for this comment, and have removed the erroneous in text citation of reference 18.

2. In the manuscript, some results were presented in Supporting Information, but the ambiguous information was provided in the text. For example, page 3, left column, lines 45-48: in the sentence "Interestingly, the sequential addition...... than the telescopic reactions (Supporting Information)". Please provide the defined information (which Table/Figure).

The reviewer makes a very good point with which we agree. The main text has been reworked to include references to figures in the SI throughout. In particular on pages 3 and 4 as the reviewer highlights.

3. In Figure 2: The results were presented by substrate conversions. However, the cascade was composed of two reactions (aminopolyol oxidation and imine reduction). The substrates aminopolyols may be converted into both the intermediate iminosugars and the target aminosugars. In addition, the formation of byproduct was observed by the authors. So, the substrate conversion is not a good index for the cascades. Likely, the product yield may be better.

We thank the reviewer for this comment and are happy to make changes as suggested. We monitored reactions by formation of product relative to starting aminopolyol due to the transient nature of reaction intermediates and have changed mention of 'conversion' to 'product formation'. We feel this term more accurately reflects the UPLC assay without

causing confusion between reactions analysed by UPLC and those where isolated yields are reported.

4. Figure S6: The title is incomplete.

We thank the reviewer for this comment, and have adjusted the table in Figure S6 (now Figure S7 after addition of SDS-PAGE) to ensure the whole figure caption is visible.

Reviewer: 2

5. In table/figure captions the use of purified enzymes/immobilised enzymes or clarified lysates should be indicated and highlighted further in the text (and concentrations used).

We thank the reviewer for this comment and have reworked the figure/table captions, main text and parts of the SI to improve clarity around what enzyme preparation was used in each case (see SI pages S9–S12)

6. Why were enzyme lysates not used more widely (which are often preferred in industry).

The reviewer makes a very good point about merits of different enzyme preparations. All recycling enzymes were applied as lysates (HRP, catalase, PtDH), however the GOase and reductases could not be efficiently used as lysate. Putative reductases were initially screened as lysates and consistently gave low conversions or multiple byproduct peaks. We believe that due to the sugar-like nature of the target compounds and substrates there is a variety of endogenous enzyme activity which can interfere with the proposed cascade (indeed, the activity of title shikimate dehydrogenases for the desired reduction would imply this). As such, we elected to investigate purified protein to improve reaction efficiency, avoid these issues and simplify downstream processing to enable single-step purification of products in good yield and selectivity (no byproducts were observed in reactions using purified reductase). We anticipate that enzyme engineering to improve desired target activity relative to endogenous background and/ or implementation of from-lysate immobilisation could improve the efficacy of enzyme lysates in the designed cascades. This is perhaps exemplified by the tolerance of recycling enzyme lysate (e.g. HRP, catalase, PtDH) which were present in low amounts and did not cause observable byproduct formation.

7. Protein SDS page gels should be added to the SI and levels of enzyme production.

We thank the reviewer for this comment, and have added copies of the SDS-PAGE analysis in the new Figure S1 (see SI page S4–5). In addition, levels of enzyme production as mg purified enzyme per litre of culture broth are now reported in the relevant sections of the SI (pages S4 and S5). These were between 200 and 250 mg/L culture broth for GOase variants, and 100 to 150 mg/L culture broth for reductase proteins.

8. In the SI the Figure 6 caption is obscured.

We thank the reviewer for this comment, and have adjusted the table in Figure S6 (now Figure S7 after addition of SDS-PAGE) to ensure the whole figure caption is visible.

9. There are several Figures in the SI, with data very briefly being mentioned in the main text. These SI Figures should be mentioned in the text to help the reader understand where this data is.

The reviewer makes a very good point with which we agree. The main text has been reworked to include references to figures in the SI throughout in order to better signpost the relevant data (see main text page 3 & 4 in particular).

oc-2022-011698.R2

Name: Peer Review Information for "Biocatalytic cascades towards iminosugar scaffolds reveal promiscuous activity of shikimate dehydrogenases"

Second Round of Reviewer Comments

Reviewer: 2

Comments to the Author

The authors have addressed all queries and acceptance of the manuscript is recommended.

Reviewer: 1

Comments to the Author

The authors have addressed the comments well. Now it may be accepted.

Author's Response to Peer Review Comments:

Thank you very much for your kind letter. We have addressed all formatting issues as raised and hope that the manuscript is now acceptable.
